# The swine IsoLoop model of the gut host-microbiota interface enables intra-animal treatment comparisons to advance 3R principles

**DOI:** 10.1080/19490976.2025.2568706

**Published:** 2025-10-25

**Authors:** Jenna Bayne, Chandrashekhar Charavaryamath, Yoonsung Hu, Farnaz Yousefi, Morgan Murphy, Andy Law, Alyona Michael, Muhammed Shafeekh Muyyarikkandy, Britt Nibbering, Wiep Klaas Smits, Ed Kuijper, Tanja Opriessnig, Mary Sauer, Joy Scaria, Brett Sponseller, Alejandro Ramirez, Shankumar Mooyottu

**Affiliations:** a Department of Clinical Sciences, College of Veterinary Medicine, Auburn University, Auburn, AL, USA; b Department of Veterinary Diagnostic and Production Animal Medicine, College of Veterinary Medicine, Iowa State University, Ames, IA, USA; c Department of Biomedical Sciences, College of Veterinary Medicine, Iowa State University, Ames, IA, USA; d Department of Veterinary Biomedical Sciences, Shreiber School of Veterinary Medicine, Rowan University, Glassboro, NJ, USA; e Department of Pathobiology, College of Veterinary Medicine, Auburn University, Auburn, AL, USA; f Department of Veterinary Clinical Sciences, College of Veterinary Medicine, Iowa State University, Ames, IA, USA; g Department of Veterinary Medicine and Surgery, College of Veterinary Medicine, University of Missouri, Columbia, MO, UK; h Department of Animal and Food Sciences, College of Agriculture & Natural Resources, University of Delaware, Newark, DE, USA; i Leiden University Center for Infectious Diseases, Leiden University Medical Center, Leiden, the Netherlands; j Department of Vaccines and Diagnostics, the Moredun Research Institute, Edinburgh, UK; k Laboratory Animal Resources, Iowa State University, Ames, IA, USA; l Department of Veterinary Pathobiology, College of Veterinary Medicine, Oklahoma State University, Stillwater, OK, USA; m Department of Veterinary and Biomedical Sciences, College of Agriculture, Food and Environmental Sciences, South Dakota State University, Brookings, SD, USA; n Department of Veterinary Microbiology and Preventive Medicine, College of Veterinary Medicine, Iowa State University, Ames, IA, USA; o Department of Veterinary Science, Martin-Gatton College of Agriculture, Food and Environment, University of Kentucky, Lexington, KY, USA; p College of Veterinary Medicine, University of Arizon, Tucsona, AZ, USA; q Department of Veterinary Pathology, College of Veterinary Medicine, Iowa State University, Ames, IA, USA

**Keywords:** Animal model, gut host-microbiota interface, 3R principles, human fecal microbiota, *P. hiranonis*, *C. difficile*

## Abstract

Understanding gut–host microbiota interactions requires models that replicate human physiology while providing region-specific resolution, translational precision, and minimal animal use. To this end, we developed the IsoLoop model, a swine gut loop platform enabling intra-animal, multi-treatment comparisons. Microbiota-depleted ileal loops were surgically created in pigs, maintaining neurovascular integrity while isolating them from the anastomosed digestive tract. In Experiment 1, loops were inoculated with human fecal microbiota (HFM) or HFM combined with *Peptacetobacter hiranonis*. In Experiment 2, they were inoculated with *Clostridioides difficile*. Host–microbiota interactions were compared with respective controls in each experiment. The IsoLoop model reduced animal use by 75% compared to conventional whole-animal designs. Following antibiotic-induced depletion, loops re-established microbial diversity by day 5, despite reduced richness and loss of taxa, including *Lactobacillus*. HFM transplantation in microbiota-depleted loops induced robust transcriptomic recovery, enriched *Akkermansia* and *Bifidobacterium*, and restored specific metabolic pathways, although taxonomic and metabolic restoration remained incomplete and divergent. *P. hiranonis* promoted normal ileum-like metagenomic functional convergence, activated epithelial repair pathways, and increased specific secondary bile acids. *C. difficile* challenge recapitulated early infection pathology in IsoLoops. The IsoLoop model offers an ethical and precise platform for investigating host–microbiota crosstalk, localized enteric pathologies, and therapeutic interventions.

## Introduction

The gastrointestinal (GI) tract is a dynamic system shaped by complex interactions among the host, microbiota, and metabolic environment. Disruption of this interface contributes to diverse gut pathologies and infectious diseases.^1^^,^^2^ Understanding these conditions and developing targeted therapies require models that accurately replicate the complexity of the human gut, particularly region-specific microbial colonization, immune responses, and metabolic environments. Several *in vitro* systems, including intestinal organoids and gut-on-a-chip devices, offer high experimental control and throughput for epithelial-intrinsic questions. Nevertheless, they typically lack intact vascular, lymphatic, and neural inputs and include limited immune-cell complexity; even when flow or reproducing regional luminal chemistry, oxygen gradients, and multi-day colonization under native perfusion remains challenging.^3^^,^^4^ *In vivo* animal models address some of these gaps, but widely used rodent models often fall short of humans in anatomical, physiological, and microbiome similarity.^5–8^ Moreover, efforts to perform studies involving the oral introduction of exogenous microbial consortia (such as human gut microbiota) in these models face several challenges, including inconsistent microbial engraftment, systemic effects, prolonged timelines, and high cost.^9-11^ These approaches also limit region-specific investigations. Swine models also exhibit similar limitations as rodent approaches.^8^^,^^12^ Both rodent and swine whole-animal strategies require large numbers of animals and offer limited reproducibility when studying localized host-microbiota interactions. *Ex vivo* ligated intestinal loop models in swine and rodent species provide controlled, region-specific platforms, but are limited by short tissue viability, lack of systemic circulation, and neurovascular regulation. *In vivo* ligated loop models enable localized inoculation under physiological conditions.^13-21^ Collectively, these models have yielded foundational insights into mucosal immunity and pathogenesis, yet they are generally short‑term (hours to days) and often optimized for single‑question readouts rather than multi‑treatment, intra‑animal comparisons over several days. They may also involve prolonged anesthesia with exteriorized intestines (typically up to 12 h), which limits microbiota‑focused studies and raises ethical and logistical concerns.^14^^,^^22^

To overcome these limitations, we hypothesized that multiple intestinal loops derived from a surgically isolated, microbiota-depleted ileal segment would provide a controlled platform for intra-animal multi-treatment comparisons while addressing the 3R principles (Reduction, Refinement, and Replacement).^23^ Similar surgical designs have previously been employed in pigs to model amebiasis using jejunal loops.^24^ Moreover, comparable ileal loop designs have been utilized in lambs and calves for immunological studies and investigations of other protozoal infections.^25-27^Swine provide high translational relevance due to their close anatomical and microbial similarity to humans, with intestinal dimensions comparable to humans, enabling convenient experimental procedures and precise manipulations.^7^^,^^12^^,^^28^^,^^29^ Critically, these isolated loops, herein referred to as “the IsoLoops,” maintain intact mesenteric attachment with native vascular and neural supply while remaining surgically disconnected from the anastomosed ileum. This design allows animals to resume normal feeding and intestinal transit within 24 h post-surgery while isolating experimental interventions (inoculations/infections) from the normal digestive tract, minimizing distress and maintaining physiological stability.

The rationale behind developing the IsoLoop model is to address key limitations of existing animal models, including the need for large cohorts and the inability to localize treatments. Each ileal loop functions as an independent experimental unit, enabling direct delivery of microbial consortia, therapeutics, or metabolites. This design supports multi-treatment studies within a single host—for example, reducing a 24-animal cohort (four treatments × six replicates) to six pigs carrying four loops each while minimizing inter-animal variability. Unlike conventional strategies requiring oral gavage or gnotobiotic facilities, our model allows precise inoculation into antibiotic-depleted loops, bypassing engraftment unpredictability.^9^^,^^10^^,^^30^ Another unique advantage is the disconnection from host bile flow, enabling independent manipulation of the luminal bile acid environment. Bile acid profiles critically regulate microbial ecology, epithelial function, and immunity in health and disease, including in *C. difficile* infection (CDI) and inflammatory bowel disease (IBD) pathogenesis.^31-34^ Bile acid conversion in the gut involves bile salt hydrolase (Bsh) for deconjugation and the bile acid-inducible (bai) operon with 7-alpha-hydroxylase activity for transforming primary to secondary bile acids; *P. hiranonis* possesses both, making it key for gut homeostasis, including protection against CDI and IBD.^35-40^ Since rodent and swine bile acid composition differs significantly from humans, the IsoLoop model’s ability to administer select or custom bile acids offers translational fidelity for studying bile acid–microbiota–host interactions.^41-45^

Collectively, the IsoLoop model addresses three key needs: (1) precise spatial control over inoculations and treatments, (2) physiological relevance of host-microbe interactions, and (3) ethical reduction of animal use via intra-animal comparisons. We present the development of this novel swine ileal loop model and its utility is demonstrated through (1) reconstitution with human fecal microbiota (HFM), (2) introduction of the non-pathogenic gut bacterium *P. hiranonis* into a humanized microbiota environment, and (3) establishment of a simplified infection model with *C. difficile*, assessing microbial dynamics, host transcriptional responses, and metabolomic shifts through integrative multi-omics analyzes.

## Materials and methods

### Human fecal microbiota preparation and bacterial cultures

The collection and processing of human fecal samples were conducted under protocols approved by the South Dakota State University Institutional Review Board (IRB #2008008-EXP) in accordance with the published protocol.^46^ Fresh fecal samples were obtained from six healthy adult human donors residing in Brookings, SD, USA, none of whom had received antibiotic treatment within the preceding 12 months. Immediately after defecation, samples were transferred into an anaerobic chamber within 10 min to preserve the viability of obligate anaerobic microorganisms. Fecal material was diluted 1:10 (w/v) in pre-reduced phosphate-buffered saline (PBS) and centrifuged at 800 × *g* to separate the bacterial fraction from fecal debris. The resulting bacterial suspension was pooled and mixed with sterile glycerol to a final concentration of 15% (v/v) as a cryoprotectant. Aliquots were stored at −80 °C until use in transplantation experiments. Taxonomic profiling of the HFM mixture was performed by whole-genome shotgun metagenome sequencing using the method described later in this section, and the taxonomic composition is outlined in Supplementary Table 1.

For Experiment 2, *C. difficile* (human origin) and *P. hiranonis* (canine origin) were cultured in a pre-reduced Brain Heart Infusion Supplemented (BHIS) medium, enriched with 5 g/L yeast extract, 0.1% sodium tautocholic acid (for *P. hiranonis*) in an anaerobic AS−500 workstation (Anaerobe Systems, Morgan Hill, CA) at 37 °C for 12 h under an atmosphere of 5% CO₂, 5% H₂, and 80% N₂. Inocula containing 10⁶ colony-forming units (CFU)/mL were used for loop inoculations.

### Animals, anesthesia, and surgical procedure

Twenty-two female pigs (*Sus scrofa*, mixed breed, 50–60 kg, ~3 months old) were selected to provide mature ileal anatomy suitable for surgical procedures while minimizing risks associated with neonates or old adults. Female pigs were chosen to reduce behavioral variability and aggression in group housing. Mixed-breed animals (commercial crosses, e.g., Yorkshire × Landrace) were sourced from a single certified herd with traceable genetic and health records, including pathogen-free status (negative for PRRS and Mycoplasma), under USDA pain category D, per Institutional Animal Care and Use Committee (IACUC) protocol IACUC−22−066. Pigs were acclimatized for at least three days before the procedure. Pre-operative preparations included fasting for 12–24 h with *ad libitum* access to water. General anesthesia was induced using a combination of dexmedetomidine (2–15 µg/kg), midazolam (0.1–0.5 mg/kg), and morphine (0.1–0.5 mg/kg) administered intramuscularly for sedation, muscle relaxation, and analgesia. After 15–20 min, intravenous (IV) access was secured, and anesthesia was induced with propofol (2–3 mg/kg) and ketamine (2–3 mg/kg). The pigs were intubated, and the maintenance of anesthesia was achieved with 0.7%−1% isoflurane in oxygen using an adult circle rebreathing system.

**Figure 1.**
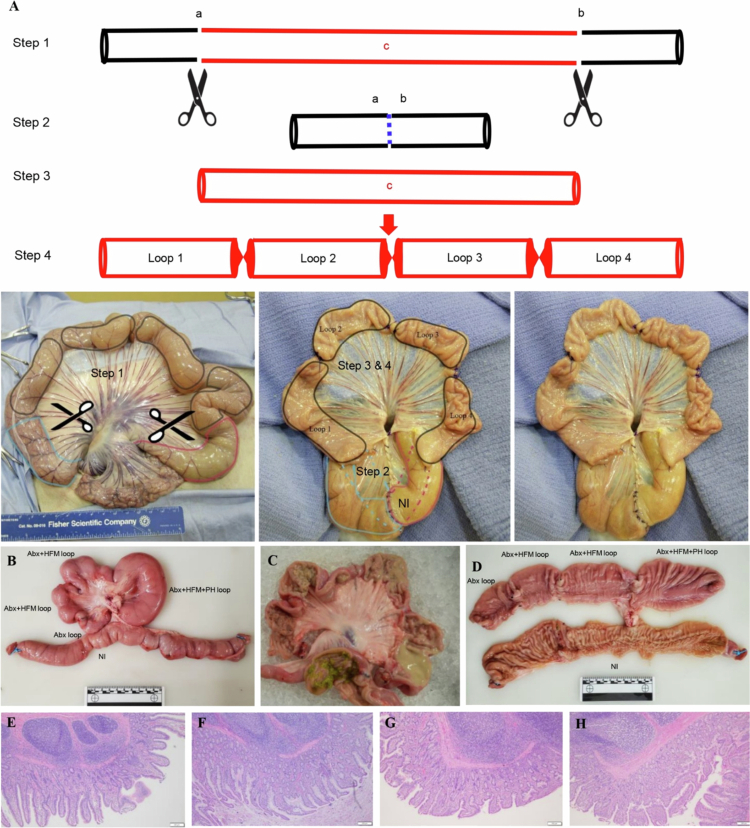
Surgical steps and gross and microscopic appearance of IsoLoops and anastomosed normal ileum: (A) Following celiotomy, the ileum is exteriorized and resected at two places (Step 1). The cut ends (a and b) are anastomosed (Step 2). The middle segment (c) is rinsed with warm PBS, incubated with antibiotic cocktails for swine ileal microbiota depletion, and ligated/stapled at three sites to create four blind-ended loops (Loops 1−4) for inoculations and interventions (Steps 3−4). (B) Gross appearance shows unopened loops and anastomosed normal ileum (NI). (C) Loops are opened with luminal content. (D) The mucosal surface of the loops is shown after removal of the contents. (E) H&E-stained histological sections of the anastomosed normal ileum (NI) showing intact villous epithelium, submucosa, and Peyer's patches. (H) Microbiota-depleted loop (Abx) is shown. (I) Microbiota-depleted loop with human fecal microbiota (Abx + HFM) is shown. (J) Microbiota-depleted loop with human fecal microbiota and *P. hiranonis* (Abx + HFM + PH) is shown.

The pig was placed in dorsal recumbency under general anesthesia. The ventrum was clipped from the sternum to the pubis and laterally to the flank fold on both sides. The site was aseptically prepared using chlorhexidine scrub and 70% isopropyl alcohol and routinely draped. The skin, subcutaneous tissue, linea alba, and peritoneum were sharply incised using a #10 scalpel blade. The cecum was exteriorized, the ileocecal ligament identified, and an approximate 35 cm length of ileum was isolated. The isolated intestine was packed off from the rest of the abdomen using sterile towels. Two sets of Doyen intestinal forceps were placed on each end of the isolated segment of the small intestine. A full-thickness, sharp incision was performed between each set of clamps orally and aborally. The edges of tissues were trimmed, and the respective oral and aboral ends were anastomosed together, leaving an intervening 24 cm length of intestine with intact mesenteric vasculature and lymphatics. Hereafter, the anastomosed, reconnected ileum is referred to as the normal ileum (NI), which serves as the intra-animal physiological control. (Figure 1A).

Anastomosis of the respective oral and aboral ends was performed using 3−0 monocryl in a simple interrupted pattern. The mesentery was closed using 2−0 Monocryl in a continuous horizontal pattern. The anastomosis site was pressure-checked, lavaged with sterile saline, and returned to the abdomen. The lumen of the isolated length of the intestine was rinsed with sterile saline three times until clear. A cocktail of antibiotic solution (enrofloxacin and metronidazole, 5 mg each, kanamycin 40 mg, and vancomycin 4 mg [Experiment 1]; or norfloxcin 1.5 mg, and moxalactam 3.5 mg [Experiment 2] dissolved in 20 ml distilled water) was then infused into the lumen, the ends sealed with Doyen forceps, and incubated for 30 min. The serosal surface of the isolated segment was moistened using sterile saline. After removal of the cocktail, the lumen was rinsed four times with sterile saline. The oral and aboral ends were trimmed and closed using a TA Premium autosuture gun with 30−3.5 mm staples and secured with 3−0 Monocryl in a continuous Cushing's pattern. A TA Premium autosuture gun with 30−3.5 mm staples was placed every 10cm to form four separate loops. Each independent loop (Figure 1A) was then inoculated with the respective treatment mixtures using a tuberculin needle inserted full-thickness into the lumen.

The needle puncture site was oversewn using 3−0 monocryl with an inverting cruciate suture. The isolated intestinal segment was copiously lavaged with saline and returned to the abdomen. The linea alba and subcutaneous tissue were closed using #2 PDS in a simple continuous pattern. The skin was closed in a simple continuous pattern using #1 nylon. No complications were encountered during the procedure, and the pig recovered from anesthesia uneventfully. See the anesthetic record for medications administered, as well as perioperative antibiotics and analgesics.

Perioperative pain management included flunixin meglumine (2.2 mg/kg, IM) and post-operative (24 h post-surgery) oral meloxicam (1 mg/kg, PO, q24 h). Pigs were monitored for recovery, with supplemental oxygen provided until swallowing reflexes returned. Animals were housed under standard husbandry conditions with regular veterinary assessments to ensure welfare.

### Experimental groups and inoculations

#### Experiment 1: microbiota consortium model

In this experiment (group 1- ten pigs), ileal loops (four loops per pig) were assigned to the following groups: one control loop exposed to an antibiotic cocktail alone (Abx loop), two antibiotic-exposed loops inoculated with 2 ml of standardized HFM mixture^46^^,^^47^ (Abx + HFM), and one antibiotic-exposed loop inoculated with both the HFM mixture and 10⁶ CFU of canine origin *P. hiranonis* (Abx + HFM + PH). Animals were euthanized on day 5 post-surgery/inoculation, and both the loops and anastomosed ileum were collected for microbiomic, metabolomic, transcriptomic, and histological analyzes. In this experiment, we compared the individual and integrated omics features of the IsoLoops across treatments and with those of the anastomosed normal ileum to evaluate the host-microbiota responses to microbial manipulations and to assess the extent to which these IsoLoops differ from or resemble the normal ileum of the host pig, thereby determining the translational fidelity of the model.

#### Experiment 2: infection model

Three pigs (group 2a) with IsoLoops received four loops each, assigned as follows: antibiotic-exposed control loops (Abx loops X 2) and antibiotic-exposed loops inoculated with 10⁶ CFU human origin (Ribotype−078) *C. difficile* spores (C. diff loops X 2). On day 5 post-surgery and inoculation, the animals were euthanized, and *C. difficile* infection in the IsoLoops was confirmed via toxin A&B-targeting ELISA using an in-house protocol.^48^^,^^49^ The ileal loops were processed for histopathological and transcriptomic analysis. This experiment was repeated using a non-toxigenic human *C. difficile* strain (group 2b). In a separate group of six pigs (group 2c), each animal received four loops assigned to the following treatments: antibiotic-exposed control loop (Abx loop), antibiotic-exposed loop inoculated with 10⁶ CFU *C. difficile* spores (Abx + C. diff loop), antibiotic-exposed loop inoculated with 10⁶ CFU *P. hiranonis* (Abx + PH loop), and antibiotic-exposed loop co-inoculated with 10⁶ CFU *P. hiranonis* and 10⁶ CFU *C. difficile* spores (Abx + PH + C. diff loop). Animals were euthanized on day 5, and the loops were examined for structural integrity to assess surgical success and complication rates. The data from groups 2b and 2c were used solely to evaluate surgical outcomes in this manuscript. Separate manuscripts will present detailed molecular and pathological results for groups 2b and 2c.

### Shotgun metagenome sequencing of loop contents, marker, and functional analysis

Whole-genome shotgun (WGS) metagenomic sequencing was performed to profile microbial taxonomic and functional composition in ileal loop samples from Experiment 1 (three samples per treatment group). DNA was extracted using the PowerSoil Pro DNA Isolation Kit (Qiagen, Hilden, Germany) with high-throughput automation on the QIAcube HT platform (Qiagen, Hilden, Germany). Mechanical lysis was achieved via bead beating using PowerBead Pro plates (Qiagen, Hilden, Germany), which contain a mixture of 0.5 and 0.1 mm ceramic beads to ensure efficient disruption of microbial cells. Extracted genomic DNA was quantified using the Quant-IT™ PicoGreen™ dsDNA Assay Kit (Invitrogen, Waltham, MA). Library preparation was carried out using a proprietary protocol adapted from the Nextera XT kit (Illumina, San Diego, CA). Sequencing was performed at Diversigen (New Brighton, MN, USA) on an Illumina NovaSeq 6000 platform, generating single-end 1 × 100 bp reads. Raw sequence reads underwent quality control using Cutadapt v2.10, which removed adapter sequences and performed quality trimming based on a Q-score threshold of 30. Reads shorter than 50 base pairs after trimming were discarded. Host-derived sequences were filtered out using Bowtie2 v2.3.0 by aligning reads to a custom host genomic DNA database.

Taxonomic and functional annotations were performed using Diversigen’s proprietary pipeline based on BURST and Venti. Sequences were aligned to a curated reference database containing all representative bacterial genomes from RefSeq, supplemented with manually curated strains. Fully-gapped alignment was conducted with a 97% identity threshold using BURST. For taxonomic assignment, reads were classified to the lowest common ancestor shared by at least 80% of the best-matching references. Functional profiling was achieved by aligning reads to a gene database linked to the Diversigen taxonomy set, identifying Kyoto Encyclopedia of Genes and Genomes (KEGG) Orthology groups (KOs) using the same 97% identity threshold. Ambiguously mapped reads were excluded from the final taxonomic and functional feature tables to ensure accuracy.

The sequencing data were processed using a customized workflow of the Divisive Amplicon Denoising Algorithm (DADA2 v1.10). This process yielded a table of amplicon sequence variants (ASVs) through denoising, incorporating quality control, primer removal, and taxonomy assignments. Cutadapt trimmed forward and reverse primers, and reads were merged to generate paired-end sequences. Quality checks ensured the adequacy of read lengths and quality. The output was a BIOM table detailing sequences, ASV abundances, and taxonomy. MicrobiomeAnalyst was utilized for all analyzes (marker data analysis and shotgun metagenome functional analysis), serving as a comprehensive tool for statistical, visual, and meta-analysis of microbiome data.^50^ It employed the MicrobiomeAnalyst R package for statistical analysis and graphical outputs. Features present in less than 20% of samples or with minimum counts below four were filtered out, and features with low variance were removed based on interquartile range criteria. Samples were rarefied to equal sequencing depth, normalized using total sum scaling (TSS), and analyzed for alpha and beta diversity, utilizing various indices and statistical methods, including principal coordinates analysis (PCoA) ordination with the Bray-Curtis index and permutational multivariate analysis of variance (PERMANOVA).^51^ Core microbiome and biomarker analyzes were performed, identifying significant features and their effect sizes. General linear models were utilized through MaAsLin2 in MicrobiomeAnalyst to identify associations between microbial or functional features and experimental metadata.^52^

### Untargeted metabolomic analysis

Untargeted metabolomic analysis of ileal contents from Experiments 1 and 2a was performed at the Mayo Metabolomic Core facility (Rochester, MN). Three samples from each treatment group were deproteinized with a six-fold volume of cold acetonitrile (1:1 ratio), subjected to intermittent vortexing on ice for 30 min at 4 °C, and then centrifuged at 18,000 × *g*. ¹³C₆-phenylalanine (3 µL at 250 ng/µL) was added as an internal standard to each sample prior to deproteinization. The supernatants were split into two aliquots and dried down for analysis using an Agilent Technologies 6550 Q-TOF Mass Spectrometer coupled with a 1290 Infinity UHPLC. Data were acquired under both positive and negative electrospray ionization conditions across a mass range of 100–1200 m/z at a resolution of 10,000–35,000 (separate runs). Metabolite separation was achieved with a hydrophilic interaction column (HILIC, ethylene-bridged hybrid 2.1 × 150 mm, 1.7 µm; Waters) and a reversed-phase C18 column (high-strength silica 2.1 × 150 mm, 1.8 µm; Waters), each with a 20-min run time at a flow rate of 400 µL/min. Each sample underwent four runs to maximize metabolite coverage. Samples were injected in duplicate or triplicate, with a quality control sample comprising a subset from the study, injected multiple times during each run.

Raw data files were converted to.cef format using MassHunter DA Reprocessor software (Agilent, Santa Clara, CA). Data alignment and peak matrix conversion were performed with Mass Profiler Professional (Agilent, Santa Clara, CA). Unsupervised principal component analysis (PCA), analysis of variance (ANOVA), 3D plots, heatmaps, and Partial Least Squares Discriminant Analysis (PLS-DA) were used for analysis. This process yielded a list of accurate mass molecular weights for differentially expressed components, which were then matched against the Metlin database for putative identification. Identified components were verified against reference standards. The Q-TOF method’s mass accuracy was <5 ppm, with retention time precision <0.2%. A 1.2 × fold change was detectable with 4% precision.

A minimum threshold of at least 5000 peak intensities in 75% of samples was set for eligibility in downstream analyzes. Data normalization, differential expression analysis, pathway analysis, and visualization were performed using the MetaboAnalystR package in R (https://www.metaboanalyst.ca). Metabolites were normalized (SumNorm), log-transformed, and mean-centered (MeanCenter). PCoA highlighted data heterogeneity, trends, and outliers. Hierarchical Clustering Analysis (HCA) elucidated clustering patterns among sample injections, group replicates, and metabolite clusters associated with clinical variables. Univariate analysis, including Student’s unpaired t-test between treatment groups, was conducted with multiple testing corrections to identify differentially expressed metabolites, requiring a false discovery rate (FDR)-adjusted *p*-value ≤ 0.05 and |fold change| ≥ 1.5.

### RNA-Seq analysis of ileal loop segments

Tissue processing and RNA-seq analysis were performed by Innomics Inc. (Sunnyvale, CA).^53^ Three samples from each treatment group in Experiments 1 and 2a were used for further processing. Total RNA from each sample was extracted and purified using the TRIzol method. RNA sequencing was then performed on a BGISEQ500 platform (Innomics Inc., Sunnyvale, CA). Twelve samples from the microbiota consortium experiment (Experiment 1) and nine from the infection experiment (group 2a) underwent multiplexing, sequencing, differential gene expression analysis, and transcriptomic expression analysis. Quality control of raw data was conducted using SOAPnuke software, and clean reads were obtained after removing rRNA and other contaminants.

The clean reads were mapped to the *S. scrofa* genome sequence using HISAT for mRNA and long non-coding RNA quantification and Bowtie2 for gene sequence alignment. For circRNA quantification, clean reads were aligned to known *S. scrofa* circRNAs. Small RNAs were identified by mapping clean tags to a miRNA database and the *S. scrofa* genome sequence. Expression levels for each gene were calculated based on the mapped reads. Differential expression analysis was performed using the DEGseq package,^54^ which models RNA-seq as a random sampling process. Under this framework, the number of reads from a gene follows a binomial distribution, which can be approximated by a Poisson distribution. Based on this statistical model, differentially expressed gene (DEG)seq applies Fisher's exact test and likelihood ratio test to identify differentially expressed genes.^55^ DEGs were identified with *p*-values less than 0.05 and log2 fold changes (FC) in miRNA expression above or below 1. DEG mRNA functional classifications and pathways were assessed using Gene Ontology (GO) Elite and DAVID Bioinformatics Resources for KEGG pathway analysis. All data were extracted, analyzed, and the results were visualized using Dr. Tom transcriptome analysis interface provided by Innomics Inc. (https://www.bgi.com/global/service/dr-tom).

### Integrative multi-omics analyzes

To investigate the combined metabolic and transcriptomic differences by surgical loop process in Experiment 1, a joint pathway analysis was conducted using MetaboAnalyst 4.0^56^ (https://www.metaboanalyst.ca). Metabolomic data were processed by normalizing metabolite intensities using the SumNorm method, followed by log transformation and mean centering through the R package MetaboAnalystR. Similarly, transcriptomic data were normalized and log2-transformed for subsequent analysis. Metabolomic and transcriptomic datasets were uploaded to the Joint Pathway Analysis module in MetaboAnalyst, where metabolites and genes were mapped to corresponding pathways using the KEGG database. Fisher's method was applied to combine *p*-values from individual metabolomic and transcriptomic analyzes to identify significantly affected pathways, yielding a joint *p*-value. Pathways with a joint *p*-value below 0.05 were deemed significantly enriched.

In addition, a microbiome-metabolome correlation analysis was performed using MicrobiomeAnalyst 2.0 (https://www.microbiomeanalyst.ca).^50^ Matched differentially and significantly abundant metabolome and species abundance tables for each comparison in Experiment 1 were uploaded into the integrative microbiome-metabolomics pipeline. Statistical correlation analysis was conducted using a distance-based correlation method, which detected both linear and non-linear associations. The findings were visualized as an interactive heatmap. To minimize false positives in pairwise correlations, a model-based correlation analysis leveraging over 5000 high-quality genome-scale metabolic models (GEMs) was applied. This approach generated a probability heatmap depicting relationships between microbial taxa and metabolites. Finally, the statistical and model-based correlation results were integrated by overlaying the respective heatmaps to provide combined data-driven and knowledge-based evidence.

### Histopathology, immunohistochemistry, and image analysis

The small intestinal tissues (ileum) from Experiments 1 and 2a were fixed in 10% formalin and subsequently embedded in paraffin. Tissue sections were cut to a thickness of 5 µm and stained with hematoxylin and eosin (H&E). Microscopic analysis of histopathologic features in IsoLoops across various treatment groups, including CDI-associated lesions, was conducted by a board-certified pathologist, blinded to sample identities, using an Olympus BX53 microscope (Olympus Optical Company, Tokyo, Japan).

The following primary antibodies were used for evaluating small intestinal restitution and immune cells in Experiment 1: monoclonal mouse anti-E-cadherin (Thermo Fisher Scientific, Carlsbad, CA, USA); polyclonal rabbit anti-lysozyme EC 3.2.1.17 (Dako, Glostrup, Denmark); monoclonal mouse anti-cytokeratin peptide 18 (Sigma-Aldrich, St. Louis, MO, USA); polyclonal rabbit anti-pIgR (Sigma-Aldrich, St. Louis, MO, USA); monoclonal mouse anti-Ki−67 (Dako, Carpinteria, CA, USA); and monoclonal mouse anti-*β*-catenin (Sigma-Aldrich, St. Louis, MO, USA). The specificity of these antibodies for swine tissue has been validated. Immunohistochemistry (IHC) on sections of the ileum was performed at the Iowa State University Veterinary Diagnostic Laboratory (Ames, IA, USA) as described in previous studies.^57^^,^^58^ Image quantitative analysis of regions positive for the antibodies above was conducted using HALO image analysis software (Indica Labs, Albuquerque, NM). Positive regions were manually annotated during the image analysis process. After analysis, immune-positive areas were calculated as one value per µm² of the mucosal epithelium or Peyer’s patches.^57^^,^^58^

### Statistical analysis

Each ileal loop was considered the experimental unit for all analyzes. Comparisons between the two groups were evaluated using independent samples t-tests (unpaired Student's t-test) to assess differences in means. For comparisons involving more than two groups, one-way ANOVA was performed, followed by Tukey's post hoc multiple comparison test to identify group-specific differences. For IHC image analysis in Experiment 1, four representative histological images were acquired per loop, with one loop assigned to each treatment condition in three biological replicate pigs (*n* = 3 per treatment). Each pig received all treatment conditions via anatomically isolated ileal loops, enabling within-subject comparisons. A linear mixed-effects model (LMM) was employed, with “treatment” specified as a fixed effect and “pig” as a random effect, and individual image measurements nested within loops. Post hoc pairwise comparisons between treatment groups were conducted using Tukey's multiple comparison test to adjust for multiple testing. Statistical significance was defined as a *p*-value ≤ 0.05.

## Results

### The IsoLoop model enables stable and controlled intra-animal treatment comparisons

To establish a robust platform for intra-animal treatment comparisons while advancing the 3 R principles, we surgically created multiple ileal loops from microbiota-depleted, parallel-resected ileal segments in live pigs, as detailed in the methods. Following celiotomy, a 35 cm segment of the ileum was exteriorized and resected at two points separated by 6−8 inches, with the cut ends anastomosed to maintain intestinal continuity. The isolated middle segment (24 cm) was thoroughly rinsed with warm PBS, treated with a 4-antibiotic cocktail to deplete the swine ileal microbiota, and subsequently rinsed four times with sterile saline and ligated at three sites to create four blind-ended compartments (Loop 1-Loop 4) (Figure 1A). The final wash after antibiotic exposure was cultured and found negative for aerobic and anaerobic bacteria. These compartments were designated for distinct treatments: antibiotic-treated (Abx), Abx with HFM (Abx + HFM), and Abx with HFM and *P. hiranonis* (Abx + HFM + PH) in Experiment 1, and various combinations involving *C. difficile* and *P. hiranonis* in Experiment 2. The loops maintained viability and functionality until scheduled euthanasia at 5 d post-surgery, confirming the model's short-term stability for experimental interventions. Animals resumed liquid feeding within 24 h post-surgery, demonstrating rapid recovery and minimal distress, aligning with the Refinement principle by reducing postoperative suffering.

Out of 22 pigs that underwent surgical experiments, four were euthanized earlier due to complications during the initial stage of the project—three due to septic peritonitis and one due to jejunal infarction—all associated with HFM inoculation in Experiment 1. The rest of the surgeries were uneventful with no complications, further validating the model's reliability when microbial introductions are carefully managed. This low overall complication rate underscores the model's alignment with the Reduction principle, as it enables multiple treatment comparisons within a single animal, significantly decreasing the total number of animals required compared to traditional whole-animal models. For instance, a conventional study requiring four treatments with six replicates each would necessitate 24 animals, whereas our model achieves the same with just six pigs, each carrying four loops.

Gross examination revealed that the surgically created loops exhibited a pale, whitened mucosal surface, consistent with their isolation from enterohepatic bile flow, in contrast to the bile-tinged epithelium of the anastomosed normal ileum (NI) (Figure 1B–D). Despite thorough washing with PBS during surgery to remove luminal contents, the closed loop lumens contained pasty material at necropsy, which histological analysis identified as sloughed epithelial debris. This accumulation mimics endogenous nutrient substrates, providing a beneficial matrix for microbial recolonization studies by offering a nutrient-rich environment for inoculated microbiota. Notably, loops inoculated with *P. hiranonis* (Abx + HFM + PH) in Experiment 1 showed visibly increased luminal content volume and loop diameter compared to adjacent non-inoculated loops (Abx + HFM), which appeared slightly contracted relative to both PH-treated loops and NI (Figure 1B–D). This observation suggests that *P. hiranonis* may enhance microbial activity or biomass accumulation, potentially through its metabolic interactions with the humanized microbiota, a hypothesis further supported by subsequent microbial and metabolomic analyzes.

Histopathological analysis at 5 d post-surgery, using H&E-stained sections, confirmed that the mucosal architecture was preserved across all loop segments (Abx, Abx + HFM, Abx + HFM + PH) in Experiment 1, with no significant abnormalities in the muscularis or serosal layers compared to NI. The presence of denuded epithelial cells in the superficial luminal layers was noted, consistent with localized epithelial turnover in the absence of luminal flow, but there was no evidence of inflammation or tissue damage, indicating that surgical isolation did not compromise tissue integrity. Compared to NI, ileal loops showed mild mucosal proliferation with sloughed epithelial debris in the superficial mucosa, reflecting a natural response to the lack of normal intestinal transit while maintaining overall structural integrity (Figure 1E–H).

**Figure 2.**
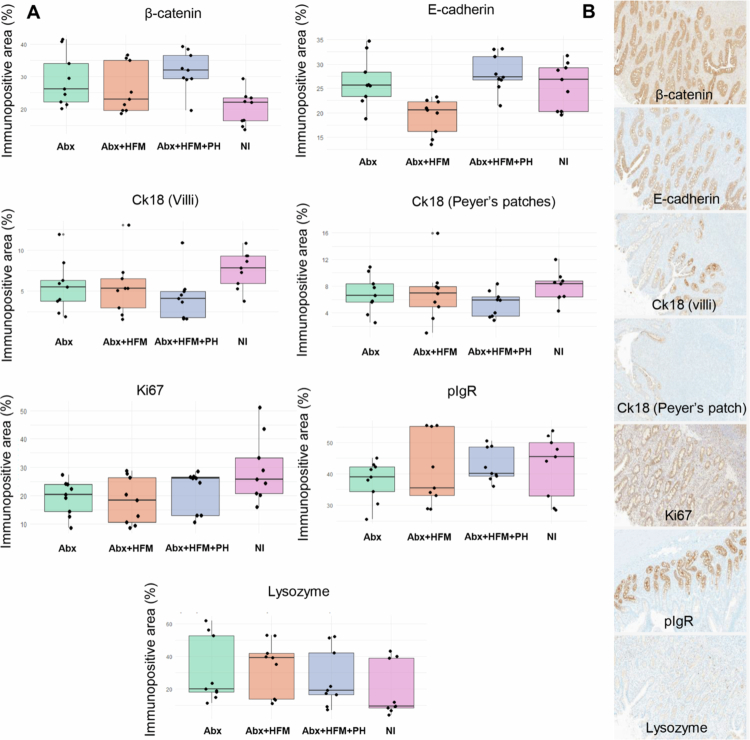
Quantitative immunohistochemical analysis of IsoLoops and anastomosed normal ileum: (A) Immunohistochemical staining is performed on ileal sections from anastomosed normal ileum (NI), microbiota-depleted loop (Abx), microbiota-depleted loop with human fecal microbiota (Abx + HFM), and microbiota-depleted loop with human fecal microbiota and *P. hiranonis* (Abx + HFM + PH) groups using validated antibodies for *β*-catenin, E-cadherin, cytokeratin peptide 18 (CK18), polymeric immunoglobulin receptor (pIgR), Ki-67, and lysozyme, with HALO software image analysis quantifying the immunopositive area relative to the selected epithelial basement area (bar plots). (B) Representative immunohistochemical images illustrate the localization of epithelial and immune markers in the anastomosed normal ileum (NI) (right panels) (*p *> 0.05 in all comparisons).

IHC analysis in Experiment 1 further validated the model's ability to preserve intestinal tissue architecture and function (Figure 2). Using validated antibodies against epithelial and immune cell markers—E-cadherin and *β*-catenin (for epithelial junctional integrity), cytokeratin 18 (CK18, an M cell marker in both mucosa and Peyer's patches), Ki-67 (proliferation), lysozyme (a Paneth cell marker in Peyer's patches), and polymeric immunoglobulin receptor (pIgR, facilitating mucosal immune defense via IgA transport)—quantitative image analysis was performed with HALO image analysis software (Indica Labs, Albuquerque, NM) (Supplementary Figure 1). The analysis measured percentage staining intensity per tissue area, normalized to the epithelial basement membrane length in the ileum, across treatment groups (NI, Abx, Abx + HFM, Abx + HFM + PH). Across all markers assessed, no statistically significant differences (*p *< 0.05) were observed between the surgically created loops and NI for most markers, confirming the preservation of intestinal epithelial structural and functional integrity following surgical isolation. Although not statistically significant, we observed numerical differences in several epithelial markers across groups. E-cadherin expression was lower in Abx + HFM compared to NI (*p* = 0.13), with Abx + HFM + PH values closer to NI (*p* = 0.70). Ki−67 staining, a marker of epithelial proliferation, was reduced in Abx (*p* = 0.36) and Abx + HFM (*p* = 0.27) relative to NI, while Abx + HFM + PH showed levels comparable to NI (*p* = 0.51). *β*-catenin staining was numerically higher in Abx (*p* = 0.15), Abx + HFM (*p* = 0.38), and Abx + HFM + PH (*p* = 0.06) compared to NI. While these observations did not reach statistical significance, they suggest patterns consistent with transcriptomic findings (described below) that indicate modulation of focal adhesion, ECM-receptor interaction, and cytoskeletal regulation pathways in microbiota-depleted loops. These exploratory findings are interpreted cautiously given the limited sample size (*n* = 3 biological replicates per treatment). In contrast, pIgR, lysozyme, and CK18 (in both villus epithelium and Peyer's patches) showed no appreciable differences among the groups (*p *> 0.05), indicating that baseline mucosal immunity, including Paneth and M cell populations critical for innate defense and antigen sampling, remained stable across loop conditions (Figure 2).

Collectively, these findings demonstrate that surgically generated ileal loops retain mucosal structural and functional integrity comparable to the native ileum while also responding to microbial interventions in a manner consistent with epithelial and immune modulation. The IsoLoop model’s ability to maintain host survivability, preserve tissue architecture, and enable multiple treatment comparisons within a single animal validates its suitability as a controlled platform for studying host-microbiota interactions, particularly in the context of the 3R principles.

### Microbial community restructuring across treatment groups reflects treatment-specific ecological and functional shifts

**Figure 3.**
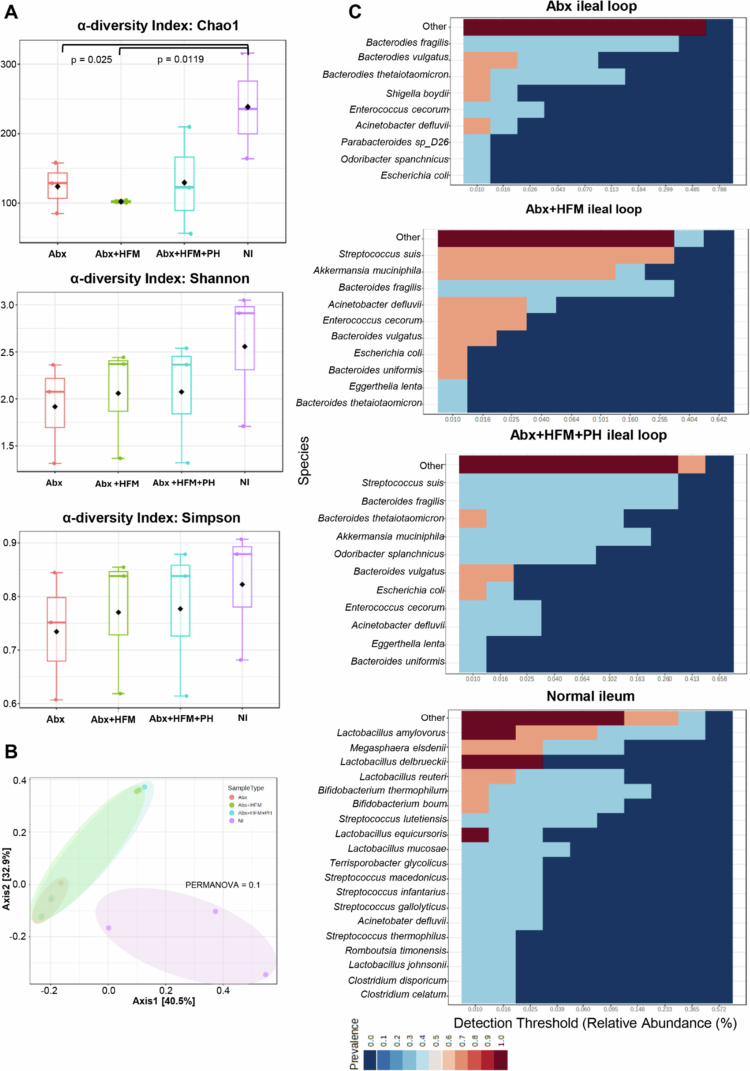
Alpha and beta diversity across treatment groups and core microbiome at the species level: (A) Boxplot illustrates the Chao1, Shannon, and Simpson indices. (B) Principal component analysis (PCoA) scatter plot demonstrates beta diversity among all groups. (C) Heatmap illustrates the core bacterial species of anastomosed normal ileum (NI), microbiota-depleted loop (Abx), microbiota-depleted loop with human fecal microbiota (Abx + HFM), and microbiota-depleted loop with human fecal microbiota and *P. hiranonis* (Abx + HFM + PH), where the core microbiome was identified based on a sample prevalence of >20% and a relative abundance frequency of >0.01% at the species level.

**Figure 4.**
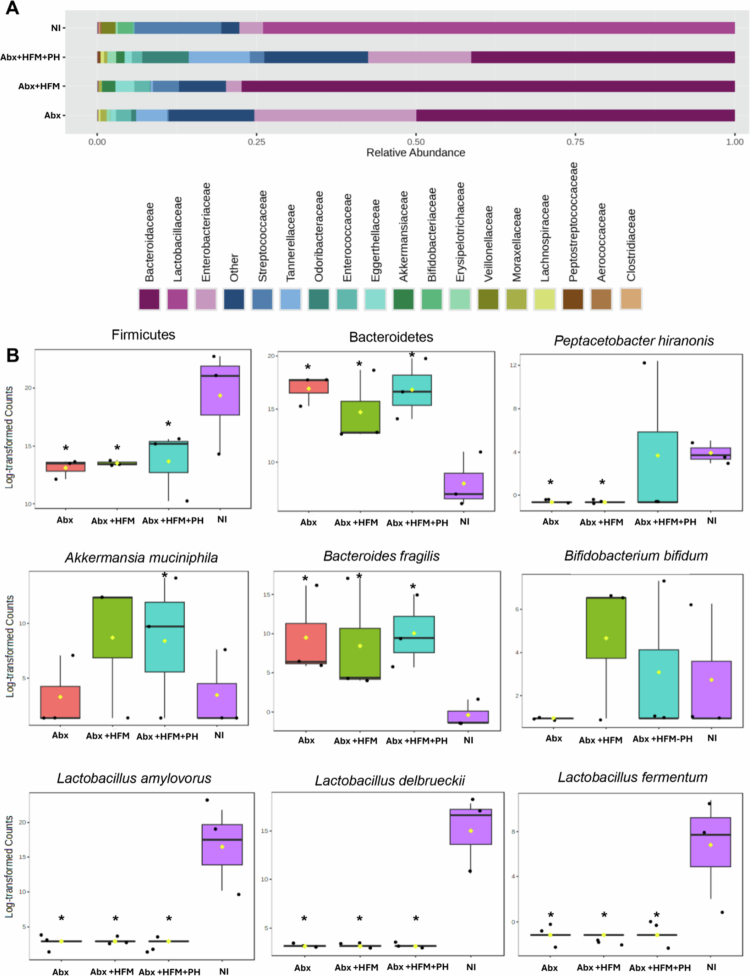
Major bacterial taxa differentially abundant across IsoLoops and anastomosed normal ileum: (A) Stacked bar graph illustrates the relative abundance of bacterial taxa at the family level across all groups. (B) Boxplot illustrates the log-transformed read counts of selected bacterial phyla and species across the treatment groups, including anastomosed normal ileum (NI), microbiota-depleted loop (Abx), microbiota-depleted loop with human fecal microbiota (Abx + HFM), and microbiota-depleted loop with human fecal microbiota and *P. hiranonis* (Abx + HFM + PH), with asterisks indicating statistically significant differences compared to the normal ileum (*p *< 0.05).

**Figure 5.**
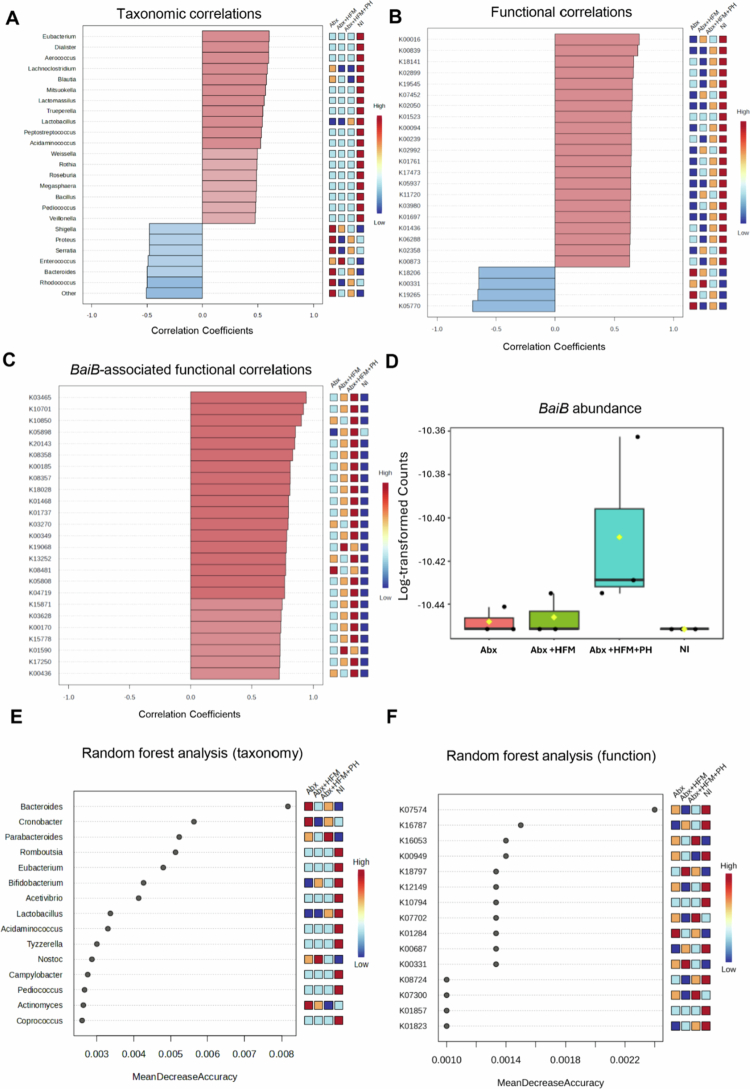
Microbial taxonomic and functional patterns and determinants across treatment groups: (A) pattern search analysis of taxonomic marker data at the genus level reveals distinct bacterial genera and correlation patterns differentiating all groups. (B) Pattern search analysis depicts the top 25 KEGG orthologs correlated with the overall functional variation among all groups. (C) Pattern search analysis displays the top 25 KEGG orthologs correlated with *baiB* (K15868) among all groups. (D) Boxplot illustrates the log-transformed read counts of *baiB* (K15868) across all groups. (E) Random forest analysis separates each group by identifying key predictive bacterial genera, ranked by their Mean Decrease Accuracy (MDA) scores. (F) Random Forest classification of functional profiles reveals KEGG orthologs that are most predictive of group separation among anastomosed normal ileum (NI), microbiota-depleted loop (Abx), microbiota-depleted loop with human fecal microbiota (Abx + HFM), and microbiota-depleted loop with human fecal microbiota and *P. hiranonis* (Abx + HFM + PH).

To evaluate the IsoLoop model's capability to study microbial reconstitution, we performed comprehensive taxonomic, diversity, and functional profiling using shotgun metagenomic sequencing and taxonomic marker profiling on ileal loop samples from Experiment 1 at 5 d post-treatment. Samples were compared across four groups: normal ileum (NI), antibiotic-treated/microbiota-depleted (Abx), Abx with HFM (Abx + HFM), and Abx with HFM and bile acid-converting bacteria *P. hiranonis* (Abx + HFM + PH). These analyzes revealed that all surgically isolated ileal loops exhibited distinct microbial compositions compared to NI, reflecting the impact of structural isolation and treatment interventions on the host-microbiome interface (Figures 3–5, Table 1). Despite lower taxonomic diversity in Abx + HFM + PH loops compared to NI, their functional profiles showed greater similarity to NI, particularly in metabolic pathways, suggesting functional restoration, although partial, despite taxonomic divergence (Figure 5).

Alpha diversity analysis, using Chao1, Shannon, and Simpson indices, revealed a progressive trend in microbial diversity across treatment groups (Figure 3A). The Abx loops showed significantly reduced species richness (Chao1, *p* = 0.025) compared to NI, though community evenness (Shannon/Simpson indices) remained stable (*p *> 0.05), indicating persistent dysbiosis with a balanced abundance distribution among remaining taxa. Interestingly, despite rigorous antibiotic treatment, thorough luminal washings before and after antibiotic exposure (with the final post-antibiotic wash negative for culturable aerobic and anaerobic bacteria), and a closed-loop design preventing incoming ingesta or microbiota, microbial diversity was re-established in the Abx loops by day 5. The Shannon and Simpson indices were not significantly different from those in normal ileum (NI) (*p *> 0.05), though the Chao1 index remained significantly lower (*p *< 0.05). This observation suggests that restored diversity and evenness may be driven by the proliferation of mucosa-associated microbiota, which are likely protected by the mucus layer and either partially shielded from antibiotics or exhibit resistance mechanisms. HFM transplantation (Abx + HFM) partially restored microbial diversity, with no significant differences in species richness or evenness compared to Abx loops (Chao1: *p* = 0.08; Shannon: *p* = 0.400; Simpson: *p* = 0.626). In the Abx + HFM + PH versus NI comparison, the Chao1 index remained significantly lower (*p *< 0.05), while Shannon and Simpson indices showed a downward trend but were not statistically significant, indicating that *P. hiranonis* further reduces richness without significantly affecting community evenness (Figure 3A). Beta diversity analysis confirmed ecological divergence, with NI clustering separately (Axis 1: 40.5% variance, Axis 2: 32.9%) from all loop groups. Abx loops formed a tight, homogenized group, while HFM- and PH-treated loops exhibited partial overlap but remained distinct from NI (PERMANOVA *p* = 0.1) (Figure 3B). The Abx + HFM versus NI comparison showed separation along Axis 1 (47.2%) and Axis 2 (25.7%), while Abx + HFM + PH versus NI showed similar separation (Axis 1: 46.9%, Axis 2: 24.2%), underscoring the enduring impact of surgical isolation on microbial ecology across all treatment conditions.

Core microbiome analysis (defined as >20% sample prevalence and >0.01% relative abundance at the species level) provided deeper insights into treatment-specific dynamics (Figure 3C). NI retained a porcine-associated core dominated by *Lactobacillus* species (*L. mucosae*, *L. reuteri*, *L. delbrueckii*, *L. amylovorus*, *L. equicursoris*, *L. johnsonii*, *L. agilis*, *L. aviari*), *Bifidobacterium* species (*B. boum*, *B. thermophilum*), *Megasphaera elsdenii*, and *Streptococcus* spp., which are critical for ileal fermentation, short-chain fatty acid (SCFA) production, and mucosal health.^59^ In contrast, Abx loops lost these commensal taxa, replaced by dysbiotic *Bacteroides* species (*B. fragilis*, *B. vulgatus*, *B. thetaiotaomicron*, *B. ovatus*, *B. xylanisolvens*) and opportunistic pathogens (*Shigella boydii*, *Enterococcus*, *Escherichia coli*, *Proteus*), reflecting a profound disruption of the host-microbiome equilibrium. HFM transplantation (Abx + HFM) introduced human-colonic taxa (*B. vulgatus*, *B. ovatus*, *B. dorei*, *B. caccae*, *Bacteroides* sp_9_1_42FAA, *A. muciniphila*, *Parabacteroides distasonis*, *Eggerthella lenta*, *P. merdae*, *B. bifidum*, *Enterococcus* sp_HMSC067C01), alongside residual porcine commensals (*E. faecalis*, *S. sanguinis*), creating a hybrid community. The near-absence of *Akkermansia* spp. in Abx loops and their resurgence in Abx + HFM loops suggest a role in epithelial restoration, given their known functions in mucin degradation and gut barrier protection. Similarly, the recovery of *B. bifidum*, a probiotic pivotal for carbohydrate metabolism and homeostasis, indicates a functional shift toward a more balanced microbial ecosystem. *P. hiranonis* inoculation (Abx + HFM + PH) further narrowed diversity, favoring *Bacteroides* dominance (*B. caccae*, *B. sartorii*, *B. finegoldii*, *B. uniformis*, *B. barnesia*) and mucin-degraders (*A. muciniphila*), while suppressing native *Lactobacillus*, *Bifidobacterium*, *Senegalimassilia anaerobia*, and *A. glycaniphila*. This selective suppression likely results from *P. hiranonis*’s competitive pressure (Figure 3C).

The phylum-level analysis highlighted substantial compositional shifts. NI was dominated by Firmicutes (*p* = 8.4389 × 10⁻⁴) and Actinobacteria, reflecting a healthy porcine gut microbiota. In contrast, Abx loops showed a significant increase in Bacteroidetes (*p* = 2.0244 × 10⁻⁴), indicative of dysbiosis following antibiotic treatment. Abx + HFM loops exhibited a reduction in Firmicutes (*p* = 7.31416 × 10⁻⁴), a modest increase in Proteobacteria (*p* = 0.34248), and a slight decrease in Actinobacteria (*p* = 0.32016), suggesting partial restoration of a humanized microbiota (Figure 4B, Supplementary Figure 2B). The Abx + HFM + PH group showed a more pronounced shift, with further depletion of Firmicutes and Actinobacteria and increased Bacteroidetes and Verrucomicrobia, reflecting targeted restructuring influenced by *P. hiranonis*. At the family level, NI was characterized by *Lactobacillaceae* (*p* = 3.168 × 10⁻⁶) and *Bifidobacteriaceae* (*p* = 9.2804 × 10⁻⁵), while Abx loops were dominated by *Bacteroidaceae* (*p* = 0.0013965) (Supplementary Figure 2A). HFM treatment induced restoration of *Akkermansiaceae* and *Bifidobacteriaceae*, suggesting beneficial shifts toward a more functionally competent community (Figure 4A).

Notably, *P. hiranonis* was detected in the NI and Abx + HFM + PH groups but was absent in the Abx loops (Figure 4B). Detection of this bacterium in the NI indicates the native *P. hiranonis* in the swine ileum. Despite lower taxonomic diversity compared to NI, functional profiles of Abx + HFM + PH loops showed greater similarity to NI, potentially driven by *P. hiranonis*-induced metabolic modulation (Described later). As observed in other treatment groups, the absence of several bile acids, including both primary and secondary forms, reflects the detachment from hepatic biliary input. Interestingly, unlike other loops, two secondary bile acids—lithocholate 3-O-glucuronide and ursodeoxycholic acid 3-sulfate were elevated in the Abx + HFM + PH loops, potentially suggesting that *P. hiranonis* drives bile acid transformation through precursors derived from other sources (Figure 14B).

Pattern search and correlation analyzes further characterized these community differences (Figure 5A, B and C). NI showed strong co-occurrence of *Lactobacillus* (*L. agilis*, *L. aviari*, *L. equicursoris*), *Turicibacter*, *Bifidobacterium*, *Dialister*, *Weissella*, *Aerococcus*, *Lachnoclostridium*, *Lactomassilus*, and *Trueperella*, reflecting a fermentative, health-associated community contributing to gut barrier function and immune homeostasis. Abx loops exhibited dominance of *Bacteroides* correlations, with *Weissella* and *Lactobacillus* showing stronger co-occurrence in NI. Abx + HFM loops were associated with *Parabacteroides*, *Enterococcus*, *Akkermansia*, *Streptococcus*, and *Bifidobacterium*, while *Shigella* and *Enterobacter* were negatively correlated, indicating suppression of opportunistic pathogens. Abx + HFM + PH loops showed positive associations with *B. barnesia*, *Streptobacillus moniliformis*, *Enterococcus* sp._HMS, *P. merdae*, *Streptobacillus*, *Cronobacter*, and *Lactococcus*, but negative correlations with *B. vulgatus*, *P. merdae*, *Enterococcus*, *E. coli*, *Actinomyces*, and *Bifidobacterium*, highlighting a community structure influenced by post-antibiotic colonizers and selective exclusion by *P. hiranonis* (Figure 5A, Supplementary Figure 3). Single-factor analysis confirmed these shifts: Abx loops showed increased Bacteroidetes and reduced Firmicutes and Actinobacteria, with enriched *B. ovatus* (*p* = 7.73 × 10⁻⁵) and *B. xylanisolvens* (*p* = 5.6 × 10⁻⁵), and depleted *L. delbrueckii* (*p* = 1.01 × 10⁻⁴), *Lactobacillus* sp. HMSC08B12 (*p* = 2.32 × 10⁻⁴), and *B. dorei* (*p* = 3.15 × 10⁻⁴). Abx + HFM + PH loops showed significant alterations in Bacteroidetes taxa, with modest shifts in Firmicutes and Proteobacteria (Supplementary Figure 4).

Random Forest classification validated these patterns. NI was characterized by *Lactobacillus*, *Coprococcus*, *Romboutsia*, and *Pediococcus*, while Abx loops were enriched in *Actinomyces*, *Cronobacter,* and *Bacteroides*. Abx + HFM loops were distinguished by *Escherichia*, *Streptococcus*, *Bifidobacterium*, *Akkermansia*, *Nostoc*, and *E. lenta*, while Abx + HFM + PH loops featured *Butyricimonas*, *Parabacteroides*, *Cronobacter*, *Streptobacillus*, *E. lenta*, *B. caccae*, and *B. sartorii*, reflecting a transition to biofilm-capable, facultatively anaerobic, or stress-adapted species (Figure 5E, Supplementary Figure 5).

Functional profiling via shotgun metagenomics and pattern search analysis revealed distinct metabolic capabilities across groups. NI was enriched in core metabolic functions, including K02970 (small subunit ribosomal protein L14), K03892 (ArsR family transcriptional regulator), K15554, K03307, K03312 (ABC-type transporters), K02532 (lactose permease), K00989 (ribonuclease), K01749 (hydroxymethylbilane synthase), K15540 (ecpD), K11082 (phnV), and K10112 (multiple sugar transport system ATP-binding protein), reflecting maintained nutrient processing capacity. Abx loops showed significant enrichment of stress-response pathways (e.g., K18206, K18574, K05770 [translocator protein], K12297, K01580 [glutathione metabolism], K10715 [oxidative stress response]) and genes related to carbohydrate metabolism (K01625, K01181), energy/redox metabolism (K00347, K00275), and motility (K02557), indicating microbial adaptation to antibiotic pressure. Abx + HFM loops were associated with KEGG orthologs involved in antibiotic resistance (K07694, vancomycin resistance-associated response regulator), carbohydrate metabolism (K16785, K16787, phosphotransferase system components), protein synthesis (K02876, ribosomal protein L15), nutrient transport (K03429, ABC transporter), and mucolytic capabilities (K02426 [cysteine desulfurase], K02120 [V/A-type H⁺/Na⁺-transporting ATPase subunit D], K01186 [sialidase], K10536 [agmatine deiminase], K15521 [D-inositol−3-phosphate glycosyltransferase], K16212 [4-O-beta-D-mannosyl-D-glucose phosphorylase], K18574 [Xaa-Xaa-Pro tripeptidyl-peptidase], K15855 [exo−1,4-beta-D-glucosaminidase]). Abx + HFM + PH loops exhibited upregulation of biofilm formation (K13654, mcbR), nucleotide salvage (K10974, codB), nitrogen metabolism (K02590), ribosomal function (K02909), stress adaptation (K01494 [dcd], K01524 [*ppx-gppA*]), and biosynthetic pathways (K03557 [*mgtE*, magnesium transporter], K03474 [fabG, fatty acid biosynthesis], K03417 [ispH, isoprenoid synthesis], K02835 [*ribA*, riboflavin biosynthesis]), reflecting enhanced microbial adaptability and resilience. Notably, bile acid metabolism genes, including *baiB* (K15868), *baiF* (K15871), and CBH/*BSH* (K01442), were most enriched in Abx + HFM + PH loops, highlighting *P. hiranonis*'s inherent role in restoring bile acid deconjugation and transformation (Figure 5B and C, Supplementary Figure 6).

**Figure 6.**
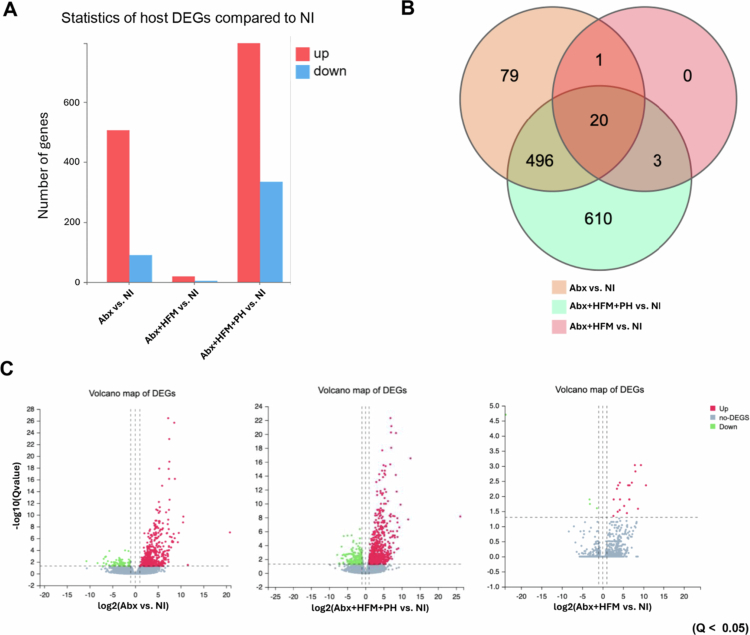
Differential gene expression patterns of IsoLoops associated with microbial reconstitution in relation to anastomosed normal ileum:(A) Differentially expressed genes (DEGs; Q < 0.05) in loop tissues compared to normal ileum (NI) are shown in a bar graph displaying the number of significantly upregulated (red) and downregulated (blue) host genes identified in microbiota-depleted loop (Abx), microbiota-depleted loop with human fecal microbiota (Abx+HFM), and microbiota-depleted loop with human fecal microbiota and *P. hiranonis* (Abx+HFM+PH) groups relative to NI. (B) Venn diagram of shared and unique DEGs across comparisons to NI, where the overlap and distribution of significantly altered genes (Q < 0.05) are shown for Abx vs. NI (orange), Abx+HFM vs. NI (pink), and Abx+HFM+PH vs. NI (green). (C) Volcano plots illustrate transcriptomic profiles of each group compared to NI, with each plot showing the log2 fold change vs. −log10 (Q-value) for all genes in Abx vs. NI (left), Abx+HFM+PH vs. NI (middle), and Abx+HFM vs. NI (right), where red dots indicate significantly upregulated genes, green dots indicate significantly downregulated genes (Q < 0.05), and gray dots represent non-significant changes.

Overall, the IsoLoop model supports modular reconstitution of the gut microbiome, with progressive but partial taxonomic and functional recovery from Abx to Abx + HFM to Abx + HFM + PH, mirroring partial structural and functional restoration of a humanized microbial ecosystem. The ability to track these changes within a single animal underscores the model's translational relevance for studying niche-specific microbial interventions (Figure 6).

### Host responses to microbiota manipulation highlight antibiotic-induced dysregulation and recovery

To investigate host responses to microbiota depletion and reconstitution, we performed comprehensive transcriptomic, metabolomic, and integrated multi-omics analyzes on samples from Experiment 1 at 5 d post-treatment, comparing treatment groups (Abx, Abx + HFM, Abx + HFM + PH) to NI. These analyzes focused on the host-microbiota interface, emphasizing bile acid influx-independent effects.

#### Antibiotic-induced dysregulation of the host-microbiota interface

**Figure 7.**
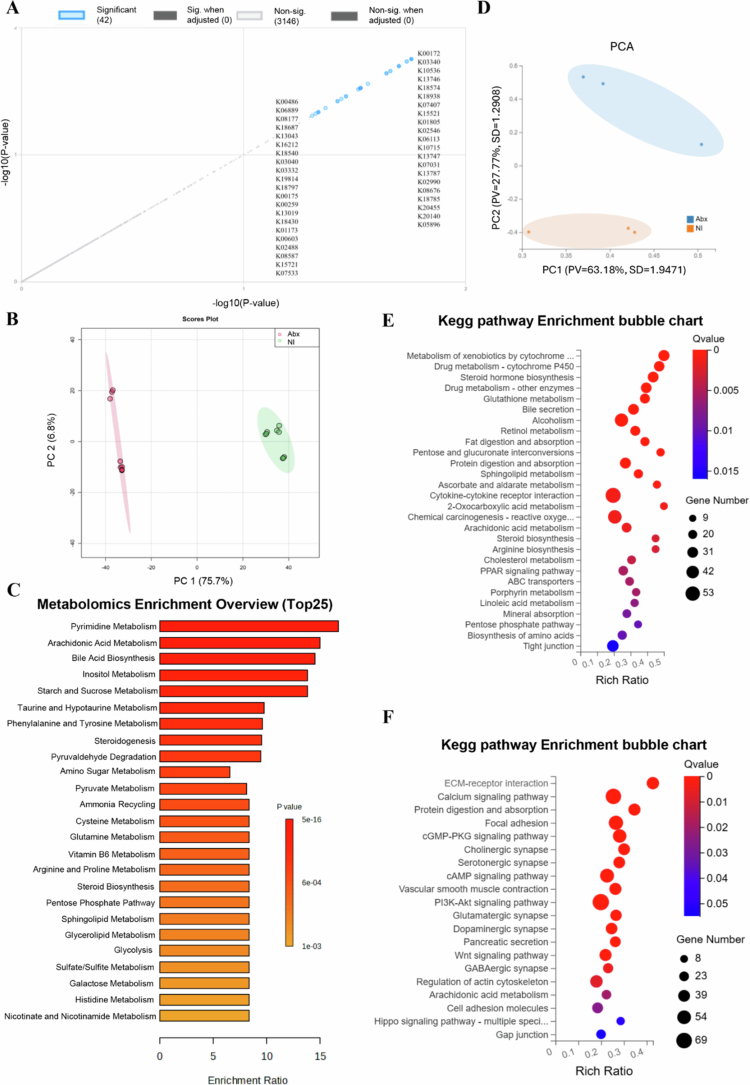
Transcriptomic and metabolomic profiles reveal functional alterations in IsoLoops following antibiotic-induced microbiota depletion: (A) Multiple regression analysis of bacterial KEGG orthologs associated with microbiota-depleted loop (Abx) and anastomosed normal ileum (NI), where each point represents one KO with −log₁₀(*p*) and blue circles denote the 42 statistically significant KOs (*p *< 0.05). (B) Principal Component Analysis (PCA) shows distinct clustering of global metabolomic profiles between Abx and NI. (C) Metabolomic pathway enrichment analysis identifies significantly enriched pathways comparing Abx vs. NI. (D) Principal component analysis (PCA) plot illustrates distinct global transcriptomic profiles of Abx and NI, with PC1 and PC2 indicating the significant variance in gene expression. (E) KEGG pathway enrichment analysis of downregulated genes in Abx compared to NI. (F) KEGG pathway enrichment of upregulated genes in Abx compared to NI.

Shotgun metagenomic functional profiling of Abx loops versus NI revealed 42 KEGG orthologs significantly associated with group differentiation (adjusted *p *< 0.05) (Figure 7A). Abx loops exhibited enrichment of stress-response pathways (e.g., K18206, K18574, K05770 [translocator protein], K12297, K01580 [glutathione metabolism], K10715 [oxidative stress response]) and genes related to carbohydrate metabolism (K01625, K01181), energy/redox metabolism (K00347, K00275), and motility (K02557), while core metabolic orthologs (K02970 [small subunit ribosomal protein L14], K03892 [ArsR family transcriptional regulator], K01595 [carbohydrate metabolism], K01232 [protein degradation], K01652 [amino acid biosynthesis], K00783 [protein synthesis]) were depleted. This indicates a systematic metabolic shift from biosynthesis to stress-response systems, consistent with the microbial dysbiosis observed in Abx loops. Random Forest-based functional classification highlighted K05770 and K01580 as the most important markers distinguishing Abx loops from NI, reinforcing the functional consequences of microbiota disruption (Supplementary Figures 6A and 7A).

Transcriptomic analysis revealed that antibiotic treatment (Abx) caused extensive transcriptional dysregulation compared to the normal ileum (NI), with a total of 596 differentially expressed genes (DEGs) (Figure 5A and B). PCA demonstrated distinct clustering (PC1: 63.18%, PC2: 27.77%) (Figure 7D), while a volcano plot for Abx versus NI showed a dense cluster of upregulated DEGs (log2FC between 2 to 10, −log10 (Q value) > 20), fewer downregulated DEGs, and a substantial number of non-significant DEGs, indicating significant perturbation of host gene expression following microbial depletion (Figure 6C). Hierarchical clustering further emphasized the divergence between groups. A Venn diagram highlighted that the Abx group has 79 unique DEGs, shares 496 DEGs with the Abx + HFM + PH group—suggesting overlapping transcriptional signatures driven by antibiotic effects—and 20 DEGs across all groups (Figure 6B). KEGG pathway enrichment analysis delineated the functional consequences: downregulated pathways in Abx loops included metabolism of xenobiotics by cytochrome P450, drug metabolism, steroid hormone biosynthesis, glutathione metabolism, fat digestion/absorption, and cytokine-cytokine receptor interactions, reflecting suppressed metabolic and immune functions, while upregulated pathways included focal adhesion, ECM-receptor interaction, regulation of actin cytoskeleton, PI3K-Akt signaling, and MAPK signaling, suggesting enhanced activation of pathways related to cell adhesion, extracellular matrix interactions, and intracellular signaling as a compensatory response to microbiota depletion, potentially influencing epithelial barrier remodeling and immune signaling (Figure 7E and F).

**Figure 8.**
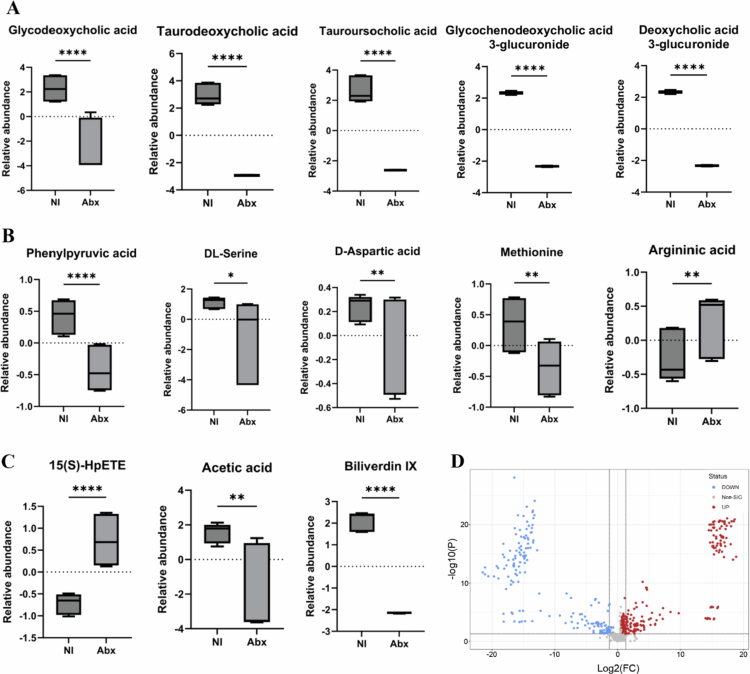
Relative abundance of differentially enriched metabolites in the microbiota-depleted loop compared to anastomosed normal ileum: (A) relative abundance of bile acid and associated metabolites. (B) Relative abundance of amino acids and associated metabolites. (C) Relative abundance of metabolites associated with inflammation, bile pigment, and microbial fermentation in microbiota-depleted loop (Abx) compared to anastomosed normal ileum (NI), with statistical significance indicated as follows: *p < 0.05; **p < 0.01; ***p < 0.001; ****p < 0.0001. (D) Volcano plot depicts differentially enriched metabolites (*p* < 0.05) comparing Abx and NI, with upregulated and downregulated metabolites marked in red and blue, respectively.

Metabolomic profiling, using four complementary chromatography modes (normal phase [NC], polar compound [PC], normal hydrophilic interaction [NHILIC], polar hydrophilic interaction [PHILIC]), revealed distinct metabolic pathway alterations in Abx loops compared to NI. PCoA, volcano plot, and hierarchical clustering showed significant metabolite changes (Figures 7 and 8), with heatmaps across all modes confirming antibiotic-induced metabolic reprogramming (Supplementary Figures 8 and 9). Biliverdin IX was significantly depleted in Abx loops, consistent with their anatomical detachment from biliary flow. Bile acid metabolism was profoundly affected, with reductions in primary and secondary bile acids (glycodeoxycholic acid, taurodeoxycholic acid, tauoursocholic acid) and their conjugated forms (glycochenodeoxycholic acid 3-glucuronide, deoxycholic acid 3-glucuronide), indicating disruption of both bile flow and microbiota-mediated bile acid transformation. Amino acid metabolism was altered, with reduced phenylpyruvic acid (reflecting disrupted phenylalanine catabolism), D-aspartic acid, DL-serine, and methionine levels, and increased argininic acid, suggesting impaired amino acid homeostasis and compensatory metabolic adaptation. Lipid metabolism showed elevated 15(S)-HpETE (an arachidonic acid oxidation product, indicating enhanced lipid peroxidation) and reduced acetic acid (a major microbially-derived SCFA), accompanied by alterations in inflammation- and oxidative stress-related metabolites. Pathway enrichment analysis confirmed significant disruptions in pyrimidine metabolism, arachidonic acid metabolism, bile acid biosynthesis, inositol metabolism, phenylalanine/tyrosine metabolism, arginine/proline metabolism, cysteine metabolism, glycolysis, and pentose phosphate pathways, reflecting widespread metabolic reprogramming (Figure 8A–C).

**Figure 9.**
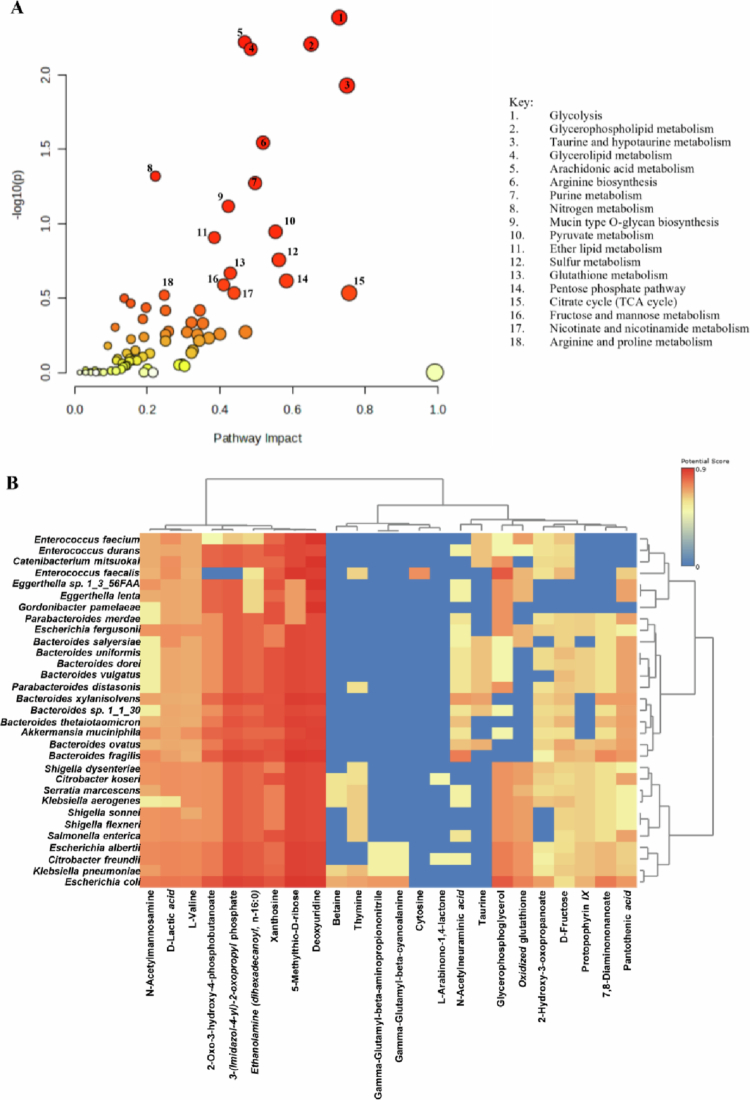
Integrated transcriptome-metabolome and microbiome-metabolome changes associated with antibiotic-induced microbiota depletion compared to anastomosed normal ileum: (A) joint pathway analysis depicts transcriptome-metabolome joint pathway analysis of microbiota-depleted loop (Abx) vs. anastomosed normal ileum (NI), where the bubble plot depicts the impact and significance of various pathways, with the size of the bubble indicating pathway impact and the color representing the −log10 (*p*) value, reflecting the significance level of the pathways. (B) Microbiome-metabolome correlation heatmap between Abx and NI displays correlations between differentially enriched key bacterial species (*p *< 0.05) and differentially enriched metabolites (*p *< 0.05), with the heatmap's color gradients illustrating the correlation coefficient between each bacterial species and metabolites.

Integrated transcriptomic-metabolomic analysis revealed coordinated dysregulation in Abx loops versus NI (Figure 9A). Pathways including glycolysis, glycerophospholipid metabolism, taurine/hypotaurine metabolism, glycerolipid metabolism, arachidonic acid metabolism, arginine biosynthesis, arginine/proline metabolism, sulfur metabolism, mucin-type O-glycan biosynthesis, pyruvate metabolism, citrate cycle (TCA cycle), and ether lipid metabolism were significantly altered, highlighting broad disruptions in central carbon, amino acid, lipid, and mucus-related biosynthesis pathways, with potential consequences for epithelial barrier function, nutrient processing, and host-microbe interactions. Shared pathways such as arginine biosynthesis and taurine/hypotaurine metabolism were altered in both transcriptomic and metabolomic datasets, indicating coordinated regulation at the host-microbiota interface (Figure 9A).

#### Transcriptomic recovery despite partial microbiota restoration following HFM transplantation

HFM transplantation (Abx + HFM) partially restored microbial communities in antibiotic-treated ileal loops compared to Abx loops alone, reintroducing key commensal taxa critical for gut health. Core microbiome analysis revealed increased relative abundance of *Akkermansia muciniphila*, *Bifidobacterium bifidum*, and *Streptococcus* species (*S. suis*-like species, *S. plurextorum*, *S. sanguinis*) in Abx + HFM loops, indicating a functional shift toward epithelial restoration and carbohydrate metabolism, with *A. muciniphila* supporting mucin degradation and *B. bifidum* aiding microbial homeostasis (Figure 3C). Functional profiling revealed enriched KEGG orthologs in Abx + HFM loops, including those related to antibiotic resistance (K07694), carbohydrate metabolism (K16785, K16787), protein synthesis (K02876), and nutrient transport (K03429), contrasting with Abx loops, which showed higher stress-response (K05770) and motility (K02557) pathways (Supplementary Figure 6B). These findings indicate HFM reintroduced microbial metabolic processes, reduced stress responses associated with dysbiosis and supported microbial competition and recolonization.

**Figure 10.**
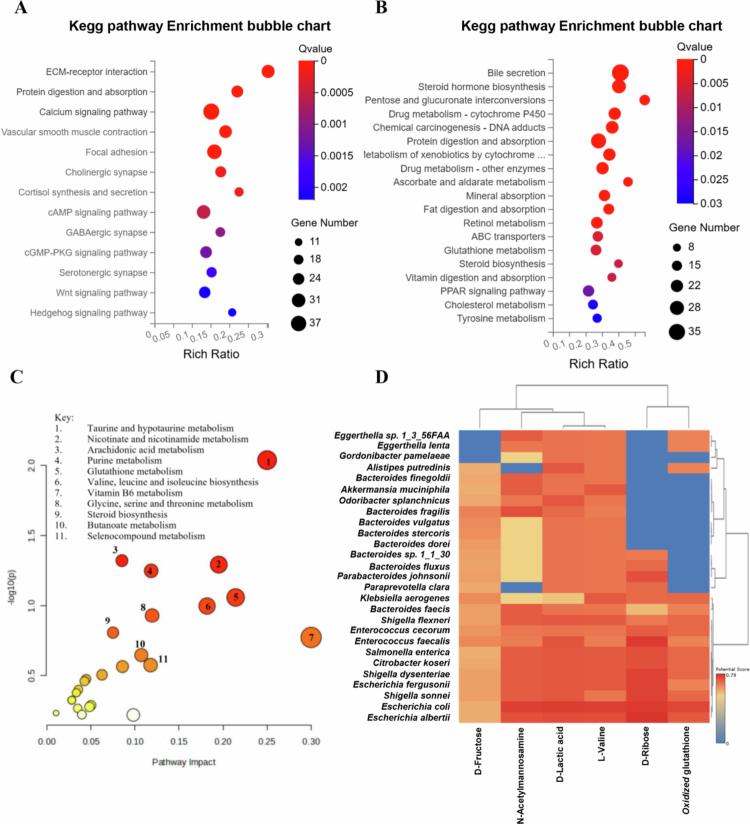
Transcriptomic changes and multi-omics correlations associated with introducing human fecal microbiota to microbiota-depleted loops: (A) Downregulated KEGG pathways in the microbiota-depleted loop with human fecal microbiota (Abx + HFM) compared to microbiota-depleted loop (Abx). (B) Upregulated KEGG pathways in Abx + HFM compared to Abx. (C) Joint pathway analysis identifies key metabolic and signaling pathways affected by HFM supplementation. (D) Microbiome-metabolome heatmap highlights correlations between differentially enriched (*p *< 0.05) bacterial species and differentially enriched (*p *< 0.05) key metabolites in Abx + HFM compared to Abx.

**Figure 11.**
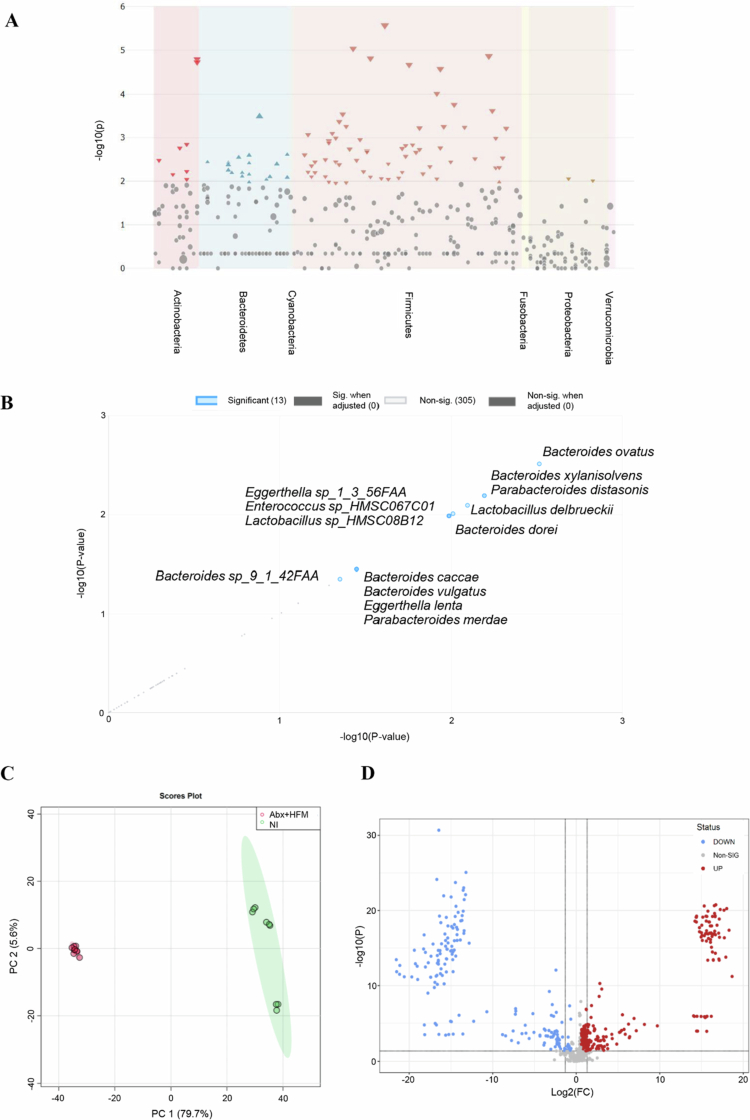
Distinct taxonomic and metabolomic divergence between humanized IsoLoops and anastomosed normal ileum: (A) single factor analysis plot depicting differentially abundant species across bacterial phyla in microbiota-depleted loop with human fecal microbiota (Abx + HFM) vs. anastomosed normal ileum (NI), where each point represents one taxon, plotted by its phylum (shaded background) along the x-axis and its –log₁₀ (*p*) value on the y-axis, with the point size reflecting the logarithmic abundance of each feature across the samples, and upward-facing triangles representing positive fold changes, whereas downward-facing triangles indicate negative fold changes. (B) Multiple regression analysis of taxonomic features at the species level associated with Abx + HFM vs. NI, where blue circles denote significantly enriched species in Abx + HFM vs. NI, gray dots are non-significant species, and dashed lines mark the unadjusted and adjusted *p* = 0.05 thresholds (−log₁₀ = 1.3). (C) Principal component analysis (PCA) reveals distinct clustering of metabolic profiles in Abx + HFM compared to NI. (D) Volcano plot depicts significantly enriched (*p *< 0.05) metabolites in Abx + HFM compared to NI.

Remarkably, despite partial microbiota taxonomic restoration, significant host transcriptomic recovery was achieved (Figure 6A, B and C). As described previously, transcriptomic analysis showed that antibiotic treatment (Abx) caused extensive transcriptional dysregulation compared to the normal ileum (NI), with 596 DEGs, mostly upregulated, and 79 unique DEGs, sharing 496 with Abx + HFM + PH and 20 across all groups, indicating significant host gene expression changes. In contrast, human fecal microbiota (HFM) transplantation (Abx + HFM) induced strong transcriptional normalization relative to NI, with only 24 DEGs identified. A volcano plot for Abx + HFM versus NI shows a sparse distribution, with few upregulated DEGs above the significance threshold, 2–3 downregulated DEGs, and mostly non-significant DEGs, while the Venn diagram reveals no unique DEGs in Abx + HFM, sharing 1 DEG with Abx and 3 with Abx + HFM + PH (Figure 6A and B). This indicates that HFM restoration closely reestablishes a normal ileum-like transcriptomic state, distinct from the antibiotic-induced profile, despite obvious taxonomic and metabolomic divergence from the normal ileum with innate swine microbiota (Figure 11). Further analysis confirmed significant gene expression changes in Abx + HFM loops compared to Abx loops, with DEG cluster analysis showing distinct transcriptomic remodeling following HFM restoration. KEGG pathway enrichment identified downregulated pathways in Abx + HFM loops, including ECM-receptor interaction, focal adhesion, and calcium signaling, suggesting reduced epithelial stress responses and remodeling, while upregulated pathways included bile secretion, steroid hormone biosynthesis, xenobiotic metabolism (cytochrome P450), pentose/glucuronate interconversions, glutathione-mediated antioxidant defense, ABC transporters, and fat/vitamin digestion/absorption, reflecting revitalized epithelial protection and nutrient processing capabilities (Figure 10A and B).

Metabolomic profiling of Abx + HFM versus Abx loops revealed limited and selective alterations, with PCA and hierarchical clustering showing overlapping clustering, indicating variable metabolic shifts (Supplementary Figure 10), while PCA comparing Abx + HFM to NI showed clear separation (PC1: 79.7%, PC2: 5.6%), highlighting persistent metabolomic differences despite transcriptomic convergence (Figure 11C). Volcano plot analysis of Abx + HFM versus Abx identified a few significantly altered metabolites, reflecting a targeted response (Supplementary Figure 10B), whereas Abx + HFM versus NI revealed reduced levels of bile acids (glycodeoxycholic acid, taurodeoxycholic acid, tauoursodeoxycholic acid, glycochenodeoxycholic acid 3-glucuronide, deoxycholic acid 3-glucuronide), biliverdin, acetic acid, *β*-D-lactose, and glucuronic acid, reflecting surgical isolation from biliary flow and diminished microbial fermentation, alongside elevated argininic acid and 15(S)-HpETE, suggesting shifts in nitrogen metabolism and inflammatory tone. Notably, aromatic amino acid metabolites like phenylpyruvic acid were depleted in Abx + HFM loops, potentially due to reduced microbial metabolism by taxa such as Bacteroides (Supplementary Figure 12). Pathway enrichment analysis indicated restored microbial contributions in Abx + HFM versus Abx through significant changes in pyruvate metabolism, pyruvaldehyde degradation, cysteine/glutathione metabolism, vitamin B6 metabolism, arginine/proline metabolism, steroid/sphingolipid biosynthesis, glycolysis, galactose metabolism, pentose phosphate pathway, and ammonia recycling (Supplementary Figure 10 C), but disruptions persisted in Abx + HFM versus NI in bile acid biosynthesis, glutathione metabolism, pyrimidine metabolism, and phenylalanine/tyrosine metabolism, underscoring incomplete recovery (Supplementary Figure 11B). The limited and selective metabolite recovery in Abx + HFM compared to Abx, alongside persistent differences from NI, suggests that transcriptomic convergence may be driven by specific metabolites or bacterial proteins rather than the full spectrum of gut metabolites, reflecting the lasting metabolic dysregulation from Abx-induced dysbiosis that delays complete restoration of host-microbiota metabolic interactions within the 5-d timepoint.

The integrated transcriptomic-metabolomic analysis confirmed coordinated recovery in Abx + HFM loops compared to Abx loops, with enriched pathways including taurine/hypotaurine metabolism, nicotinate/nicotinamide metabolism, arachidonic acid metabolism, glutathione metabolism, valine/leucine/isoleucine biosynthesis, glycine/serine/threonine metabolism, vitamin B6 metabolism, and selenocompound metabolism, reflecting renewed microbial functions in mucosal protection, nutrient processing, and cofactor production. Shared pathways such as arginine biosynthesis and taurine/hypotaurine metabolism were altered in both transcriptomic and metabolomic datasets, highlighting their dual regulation at the host-microbiota interface (Figure 10C).

These results (summarized as Table 1) underscore the IsoLoop model's value in exploring host-microbiota interactions, providing a controlled platform to investigate the introduction of specific exogenous microbial communities with metabolomic and functional significance and their translational potential.

#### Distinct host responses to P. hiranonis enrichment

*P. hiranonis* colonization (Abx + HFM + PH) further modulated host responses independent of bile influx. While Abx + HFM + PH loops exhibited lower taxonomic diversity compared to NI, their functional profiles showed greater similarity to NI (Figure 5B), particularly in metabolic pathways, suggesting partial microbial functional restoration despite taxonomic divergence. However, the introduction of *P. hiranonis* resulted in profound host transcriptional modulation compared to NI, despite detachment from the biliary circulation.

**Figure 12.**
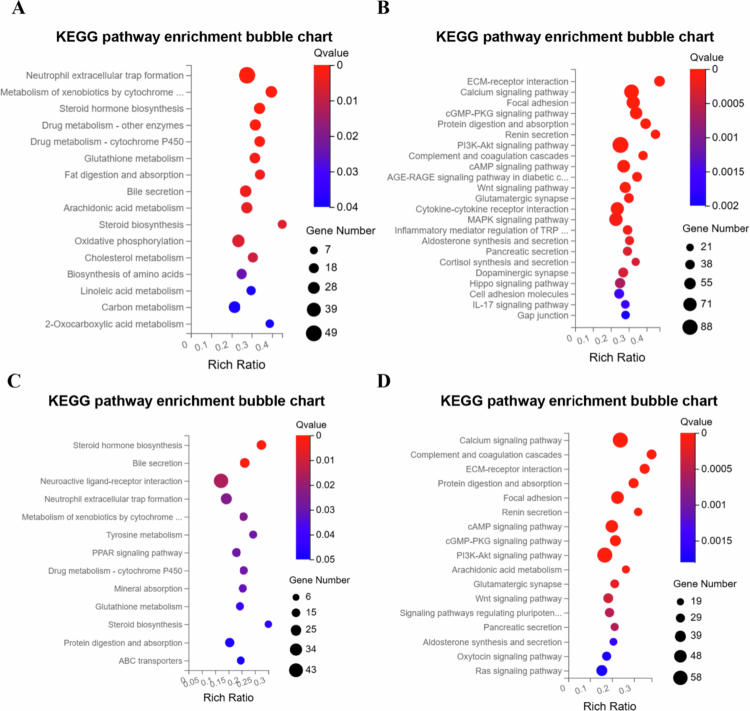
Differentially enriched transcriptomic pathways following *P. hiranonis* enrichment of humanized IsoLoops: (A) KEGG enrichment plot displays pathways downregulated in Abx + HFM + PH compared to anastomosed normal ileum (NI). (B) KEGG enrichment plot displays pathways upregulated in Abx + HFM + PH compared to NI. (C) KEGG enrichment plot displays pathways downregulated in microbiota-depleted loop with human fecal microbiota and *P. hiranonis* (Abx + HFM + PH) compared to microbiota-depleted loop with human fecal microbiota (Abx + HFM). (D) KEGG enrichment plot displays pathways upregulated in Abx + HFM + PH compared to Abx + HFM.

The introduction of *P. hiranonis* resulted in a substantial transcriptional response, with 1129 DEGs compared to NI, including 610 unique DEGs and 496 shared with Abx, indicating a distinct transcriptional program potentially linked to epithelial repair, immune modulation, or metabolic adaptation, despite taxonomic divergence and partial functional convergence in microbial pathways (Figure 6B). Compared to NI, Abx + HFM + PH loops showed pronounced suppression of bile secretion pathways, steroid hormone biosynthesis, xenobiotic metabolism, and glutathione metabolism, alongside greater upregulation of focal adhesion, ECM-receptor interaction, PI3K-Akt, MAPK signaling, cytokine signaling, chemokine pathways, and arginine biosynthesis, reflecting immunometabolic adaptation and tissue repair in response to microbial recolonization (Figure 12A and B). Similarly, transcriptomic analysis of Abx + HFM + PH versus Abx + HFM loops also showed significant downregulation of pathways related to bile secretion, steroid hormone biosynthesis, neuroactive ligand-receptor interaction, cytochrome P450-mediated xenobiotic metabolism, PPAR signaling, tyrosine metabolism, and glutathione metabolism, suggesting reduced metabolic and stress responses. Upregulated pathways included focal adhesion, ECM-receptor interaction, PI3K-Akt, cAMP, cGMP, calcium signaling, complement/coagulation cascades, and arachidonic acid metabolism, indicating enhanced epithelial barrier integrity, immune readiness, and mucosal repair (Figure 12C–D).

**Figure 13.**
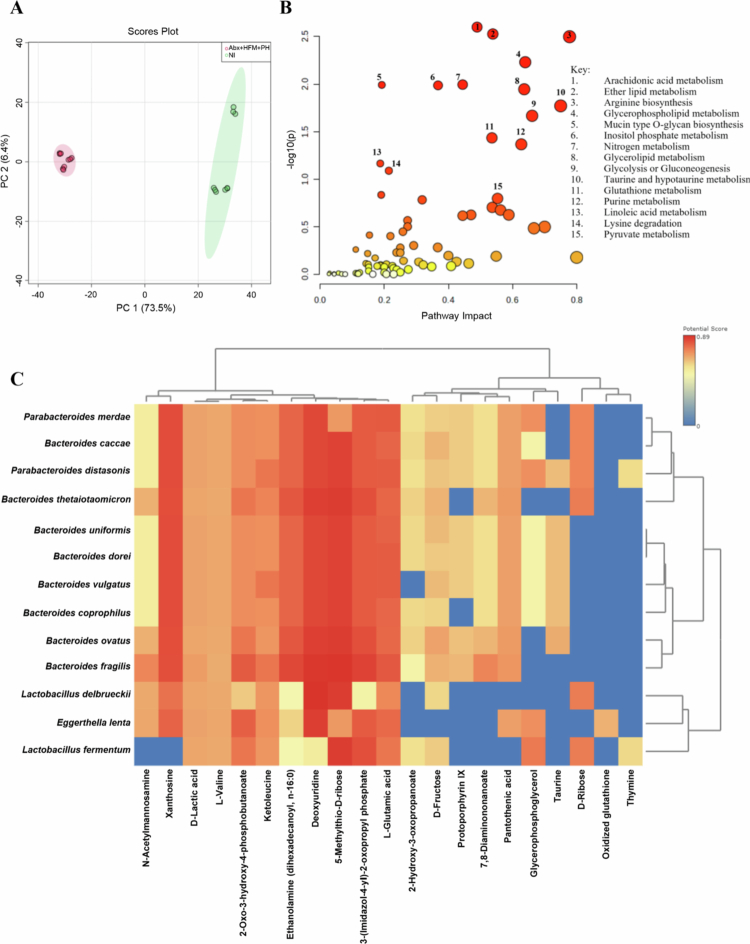
Transcriptome-metabolome and microbiome-metabolome joint pathway analysis of *P. hiranonis*-enriched humanized IsoLoops compared to anastomosed normal ileum: (A) metabolomic PCA plot shows group-wise separation between microbiota-depleted loop with human fecal microbiota and *P. hiranonis* (Abx + HFM + PH) and anastomosed normal ileum (NI). (B) Joint pathway analysis integrates transcriptomic and metabolomic data to identify significantly impacted pathways in Abx + HFM + PH vs. NI. (C) Microbiome-metabolome correlation heatmap highlights associations between dominant bacterial species and key metabolites in Abx + HFM + PH vs. NI.

**Figure 14.**
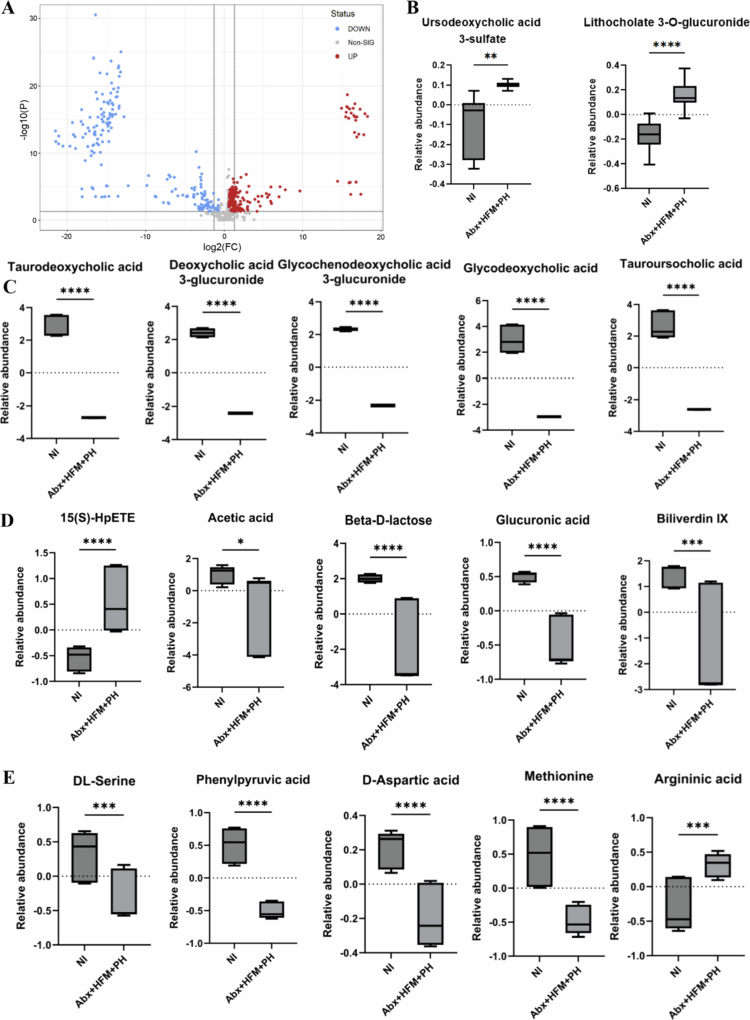
Relative abundance of selected ileal metabolites in humanized IsoLoops enriched with *P. hiranonis* compared to anastomosed normal ileum: (A) volcano plot depicts differentially enriched metabolites in microbiota-depleted loop with human fecal microbiota and *P. hiranonis* (Abx + HFM + PH) compared to anastomosed normal ileum (NI). (B) Relative abundance of bile acids and associated metabolites. (C) Relative abundance of additional bile acids and associated metabolites. (D) Relative abundance of selected metabolites associated with inflammatory signaling (15(S)-HpETE), microbial fermentation (acetic acid, *β*-D-lactose), bile pigment (biliverdin IX), and metabolite conjugation in Abx + HFM + PH compared to NI. (E) Relative abundance of amino acids and associated metabolites, with statistical significance indicated as follows: *p <;0.05; **p < 0.01; ***p < 0.001; ****p < 0.0001.

While the abundance of several primary and secondary bile acids was reduced, similar to other loops, an interesting finding in Abx + HFM + PH loops was the elevation of two secondary bile acids, lithocholate 3-O-glucuronide and ursodeoxycholic acid 3-sulfate, compared to NI despite the absence of bile influx (*p *< 0.05) (Figure 14B). No significant differences in these bile acid levels were observed between Abx and Abx + HFM, Abx + HFM and Abx + HFM + PH, or Abx + HFM and NI (*p *> 0.05). Lipid metabolites like 15(S)-HpETE and prostaglandin D2 (PGD2) were significantly elevated, indicating increased inflammatory tone from the host side, while acetic acid, *β*-D-lactose, glucuronic acid, D-aspartic acid, DL-serine, methionine, and phenylpyruvic acid were depleted, reflecting altered carbohydrate, amino acid, and microbial fermentation pathways (Figure 14C–E). Metabolomic profiling of Abx + HFM + PH versus Abx + HFM loops revealed limited global disruption, with volcano plot and heatmap analyzes showing modest differences in metabolite abundance (Supplementary Figure 13B). Pathway enrichment identified significant changes in amino sugar metabolism, pyruvaldehyde degradation, pyruvate metabolism, arachidonic acid metabolism, and bile acid biosynthesis (Supplementary Figure 13C). *N*-acetylmannosamine (a precursor in sialic acid biosynthesis) and pseudouridine (indicative of RNA turnover) were significantly downregulated in Abx + HFM + PH loops, suggesting reduced epithelial stress or RNA turnover, potentially reflecting a protective effect of *P. hiranonis* (Supplementary Figure 13D, E). Compared to NI (NC18-mode metabolomics), Abx + HFM + PH loops showed clear clustering (PC1: 73.5%, PC2: 6.4%), with pathway enrichment in bile acid biosynthesis, pyrimidine metabolism, inositol metabolism, starch/sucrose metabolism, and phenylalanine/tyrosine metabolism (Figure 13A, Supplementary Figure 14C).

Integrated transcriptomic-metabolomic analysis of Abx + HFM + PH versus Abx + HFM loops highlighted enriched pathways including mucin-type O-glycan biosynthesis, taurine/hypotaurine metabolism, glycerophospholipid metabolism, arginine biosynthesis, glutathione metabolism, arachidonic acid metabolism, linoleic acid metabolism, purine metabolism, and lysine degradation. Overlapping pathways such as arginine biosynthesis and taurine/hypotaurine metabolism were altered in both datasets, suggesting coordinated regulation at the host-microbiota interface (Supplementary Figure 15A). Compared to NI, Abx + HFM + PH loops showed additional enrichment in ether lipid metabolism, nitrogen metabolism, glycolysis/gluconeogenesis, pyruvate metabolism, inositol phosphate metabolism, and purine metabolism, with stronger mucin-type O-glycan biosynthesis and arginine biosynthesis compared to Abx + HFM loops, indicating *P. hiranonis*-specific effects on mucosal remodeling and nitrogen metabolism. Taurine/hypotaurine metabolism was consistently enriched, suggesting bile influx-independent microbial influence, potentially mediated by *P. hiranonis*'s metabolic capabilities (Figure 13B).

These findings (Table 1) highlight the IsoLoop model's utility in dissecting host-microbiota interactions, offering a controlled platform to study the introduction of a specific bacterium of metabolomic and functional interest and its translational potential.

#### Microbiome-metabolome correlations highlight specific functional reestablishment across treatment groups and metabolic modulation by P. hiranonis

Integrated microbiome-metabolome correlation analysis in Experiment 1 revealed significant associations between differentially enriched bacterial taxa and metabolites across treatment groups, highlighting the progressive functional reestablishment of microbial-metabolite networks. These correlations underscore how microbial interventions—antibiotic depletion, human fecal microbiota (HFM) transplantation, and *P. hiranonis* enrichment—reshape metabolic niches at the host-microbiota interface, aligning with taxonomic shifts and transcriptomic profiles reported in the IsoLoop model.

In antibiotic-treated (Abx) loops compared to normal ileum (NI), *Bacteroides* species (*B. salyersiae*, *B. xylanisolvens*, *B. ovatus*, *B. vulgatus*, *B. dorei*, *B. uniformis*, *B. fragilis*, *B. thetaiotaomicron*) and *Enterobacteriaceae* members exhibited strong positive correlations with metabolites linked to carbohydrate metabolism and amino acid turnover, including *N*-acetylmannosamine, D-lactic acid, 2-oxo−3-hydroxy−4-phosphobutanoate, 3-(imidazol−4-yl)-2-oxopropyl phosphate, ethanolamine (dihexadecanoyl, *n*-16:0), 5-methylthio-D-ribose, deoxyuridine, xanthosine, and L-valine. These associations suggest enhanced fermentation and nutrient scavenging by residual mucosa-associated taxa, consistent with the reduced microbial diversity and loss of commensals such as *Lactobacillus* and *Bifidobacterium* reported earlier. Notably, opportunistic or pathogenic bacteria (*Shigella* spp. [*S. dysenteriae*, *S. sonnei*, *S. flexneri*], *Klebsiella* spp. [*K. aerogenes*, *K. pneumoniae*], *Citrobacter koseri*, *Salmonella enterica*, *E. coli*) and *Enterococcus faecalis* correlated with oxidized glutathione and glycerophosphoglycerol, indicating heightened oxidative stress and membrane lipid degradation—key features of a dysbiotic gut environment. These extensive correlations reflect a profoundly disrupted metabolic landscape, aligning with transcriptomic evidence of upregulated stress-response pathways in Abx loops (Figure 9B).

HFM transplantation in Abx + HFM loops partially restored microbial-metabolite interactions, with fewer significant correlations compared to the Abx vs. NI comparison, reflecting incomplete functional recovery. This reduction in correlations is significant, as it suggests that, while HFM reintroduces microbial diversity, it does not fully reestablish the complex metabolic interplay of a healthy gut. *Bacteroides* species (*B. finegoldii*, *B. dorei*, *B. stercoris*, *B. fluxus*) correlated with D-fructose, *N*-acetylmannosamine, and L-valine, indicating renewed microbial processing of dietary carbohydrates and amino acids introduced via HFM. Fermentation-associated metabolites, such as lactic acid and D-ribose, correlated with *E. coli*, *E. faecalis*, and *S. flexneri*, pointing to partial recovery of energy-generating pathways (Figure 10D). Compared to NI, Abx + HFM loops showed *Bacteroides* species, *Parabacteroides distasonis*, *Bifidobacterium bifidum*, *Akkermansia muciniphila*, and *Enterobacteriaceae* correlating with L-glutamic acid, D-fructose, 5-methylthio-D-ribose, deoxyuridine, xanthosine, and L-valine, suggesting active amino acid and carbohydrate metabolism. However, opportunistic pathogens (*Shigella* spp., *K. aerogenes*, *C. koseri*, *S. enterica*, *E. coli*) remained linked to oxidized glutathione, protoporphyrin IX, D-ribose, and glycerophosphoglycerol, indicating persistent redox imbalance (Supplementary Figure 16B). These extensive correlations, similar to Abx vs. NI, highlight ongoing dysbiosis, consistent with incomplete microbial recovery noted in taxonomic analyzes.

The addition of *P. hiranonis* in Abx + HFM + PH loops further modulated microbial-metabolite networks, with fewer but more targeted correlations compared to Abx + HFM vs. Abx, suggesting a shift toward functional stabilization. This streamlined correlation profile underscores *P. hiranonis*'s role in refining microbial-metabolite interactions toward a balanced state. *Bacteroides* species, *Enterococcus*, and *Enterobacteriaceae* maintained correlations with D-lactic acid, and glycolic acid indicating continued fermentative activity. Interestingly, *A. muciniphila* and *E. coli* correlated strongly with *N*-acetylmannosamine, a glycan intermediate, suggesting active mucin degradation and epithelial remodeling, which aligns with transcriptomic evidence of upregulated mucosal repair pathways (Supplementary Figure 15B).

In Abx + HFM + PH loops compared to NI, *Bacteroides* species, *L. delbrueckii*, and *L. fermentum* correlated with metabolites supporting redox homeostasis (taurine, glutamic acid), amino acid biosynthesis (L-valine), and glycan metabolism (5-methylthio-D-ribose). Most strikingly, *Lactobacillus delbrueckii* and *L. fermentum*—absent in Abx and Abx + HFM comparisons—emerged with significant correlations to amino acids (L-valine, glutamic acid) and carbohydrates (D-lactic acid, 5-methylthio-D-ribose) in Abx + HFM + PH vs. NI. These unique, beneficial *Lactobacillus* correlations indicate a *P. hiranonis*-mediated shift favoring commensals critical for fermentation, nutrient utilization, and gut barrier integrity, contrasting with the dysbiotic profiles of earlier groups (Figure 13C). The increased abundance of *baiE* and *baiF* genes in Abx + HFM + PH loops, driven by *P. hiranonis*, likely contributed to these correlations by enhancing secondary bile acid transformation (e.g., lithocholate 3-O-glucuronide, ursodeoxycholic acid 3-sulfate), shifting the metabolic environment to favor taxa linked to beneficial metabolites such as taurine and short-chain fatty acids (SCFAs) (Figure 14B).

A remarkable observation was the absence of correlations between opportunistic *Enterobacteriaceae* and oxidized glutathione in this comparison, unlike the extensive correlations in Abx vs. NI and Abx + HFM vs. NI, suggesting *P. hiranonis* mitigates oxidative stress associated with dysbiosis. This aligns with transcriptomic evidence of enhanced glutathione metabolism and reduced inflammation, highlighting *P. hiranonis*'s protective role. The fewer differences in functional correlations between Abx + HFM + PH and NI, compared to the dramatic differences in Abx vs. NI and Abx + HFM vs. NI, indicate greater metabolic normalization with *P. hiranonis*, reflecting a convergence toward NI-like microbial-metabolite networks.

The establishment of microbial-metabolite correlations—from the extensive dysbiotic associations in Abx loops to partial recovery with HFM and near-normalization with *P. hiranonis*—demonstrates the IsoLoop model's precision in dissecting functional host-microbe-metabolite networks. Key insights include *N*-acetylmannosamine's consistent linkage to *Bacteroides* and *A. muciniphila* for glycan metabolism, critical for epithelial integrity, and the reduction of oxidative stress through *P. hiranonis*-mediated metabolic restructuring, as evidenced by the unique *Lactobacillus* correlations and diminished Enterobacteriaceae-oxidized glutathione associations. These findings (Table 1), enabled by the model's ability to isolate microbial interactions in a controlled, bile influx-independent setting, offer translational insights into therapeutic strategies for dysbiosis-driven conditions, such as IBD and CDI, by elucidating how targeted microbial interventions restore gut homeostasis.

#### Tissue responses to C. difficile infection in IsoLoops reflect early CDI pathology

To further validate the IsoLoop model as a platform for studying gut pathogenesis, we assessed host responses to antibiotic-induced *C. difficile* infection (CDI) in a separate experiment, focusing on ileal loops inoculated with *C. difficile* compared to uninfected antibiotic-exposed control loops (Abx loops), simulating established antibiotic-induced CDI models in rodents.^60^^,^^61^ This experiment aimed to characterize the model's ability to replicate early CDI lesions and inflammatory responses, leveraging the controlled environment of the ileal loops to isolate infection dynamics. On day 5 post-surgery and inoculation, the animals were euthanized, and *C. difficile* infection in the IsoLoops was confirmed via toxin A/B-targeting ELISA using an in-house protocol.^48^^,^^49^

**Figure 15.**
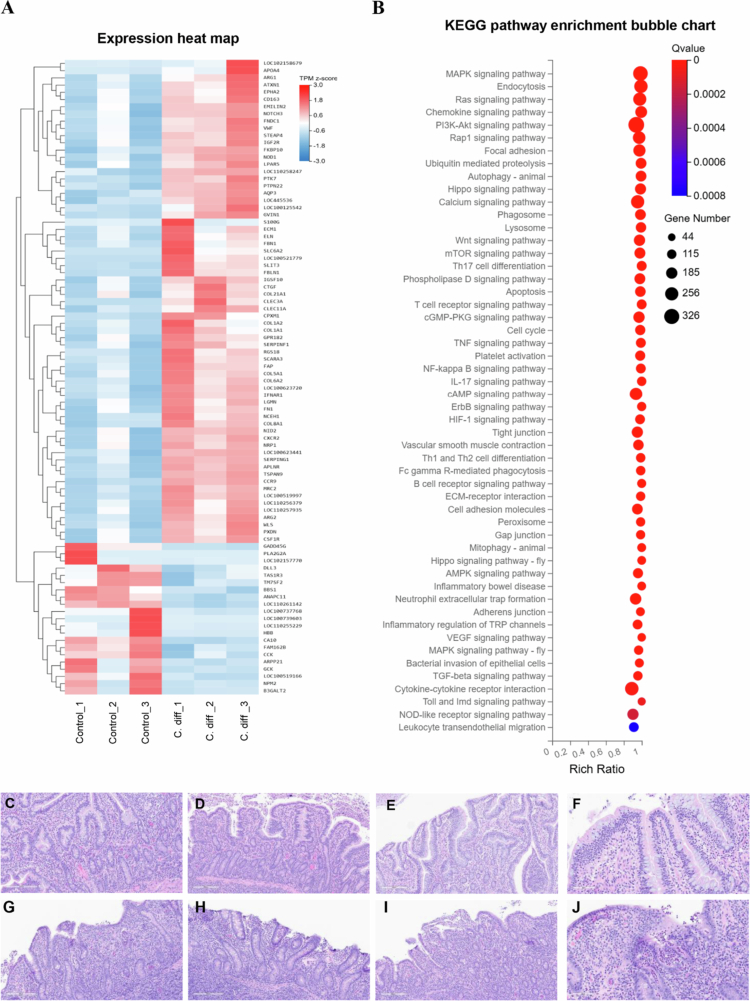
Transcriptomic and histological alterations in *C. difficile*-infected IsoLoops compared to control: (A) Hierarchical clustering heatmap depicts differentially expressed genes (DEGs) between *C. difficile*-infected loops (Abx + C. diff) and control (microbiota-depleted loop - Abx). (B) KEGG pathways show differentially enriched pathways in Abx + C. diff compared to Abx. (C-F) Top histology panels show control loops (Abx) with respective identical (in scale) microscopic fields displaying histologically normal surface epithelium, lamina propria, and submucosa for comparison with the bottom panels showing *C. difficile* infected loops (G–J; Abx + C. diff). (G) Histology image Abx + C.diff loop villi that are attenuated and blunted, with the crypt lumen occasionally containing necrotic debris with numerous viable and degenerate neutrophils (crypt necrosis and abscesses) extending into the lamina propria. (H) Ulceration and loss of villi with extensive infiltration of neutrophils extend to a mildly edematous lamina propria containing capillaries lined by plump and reactive endothelium. (I) Superficial epithelial and glandular neutrophilic infiltration is shown. (J) Superficial epithelial and glandular neutrophilic infiltration is shown at higher magnification.

RNA sequencing analysis of ileal loops inoculated with *C. difficile* (Abx + C. diff loops) compared to uninfected control loops (Abx loops) revealed significant alterations in host gene expression profiles at 5 d post-inoculation (Figure 15A). KEGG pathway enrichment analysis revealed significant upregulation of inflammatory and immune response pathways in Abx + C. diff loops, including MAPK signaling, TNF signaling, PI3K-Akt signaling, chemokine signaling, and cytokine-cytokine receptor interactions (adjusted *p* (Q) < 0.05) (Figure 15B). These pathways reflect a robust inflammatory response to *C. difficile* infection, consistent with CDI-induced mucosal pathogenesis.^62^

Histopathological analysis of Abx + C. diff loops revealed characteristic CDI lesions, including surface epithelial and crypt necrosis with erosion and ulceration, and crypt abscess formation, accompanied by neutrophil infiltration of the lamina propria, mucosal edema, and loss of villi, consistent with early CDI pathology.^63^ In contrast, Abx loops exhibited preserved mucosal architecture with absent inflammation (Figure 15C–J). These results demonstrate that the IsoLoop model in swine effectively captures the early stages of *C. difficile* infection, providing a controlled platform to study pathogen-induced mucosal responses and host–pathogen interactions. The ability to isolate infection dynamics within individual loops, coupled with detailed transcriptomic and histopathological analyzes, underscores the model’s utility for studying infectious diseases with high translational relevance.

## Discussion

The IsoLoop model demonstrates a practical and effective approach to studying host-microbiota interactions, offering a controlled, ethical platform that aligns with the 3 R principles while delivering detailed insights into gut ecology, microbial therapeutics, and infectious disease dynamics.^23^ Several *in vivo* surgical loop models have been established in large animals.^24-27^ In lambs, surgically isolated, microbiota-free intestinal segments sustain mucosal immune responses and enable independent loop-level investigations of immunomodulators and early host–pathogen interactions. This neonatal lamb gut-loop model evaluated yeast cell wall fractions and showed a modest reduction in early *Cryptosporidium parvum* development, providing a platform for neonatal enteric-disease studies.^26^ Jejunal loop models in pigs have been used to study mucosal responses to *Salmonella enterica*, demonstrating loop-level mRNA expression changes (cytokines, chemokines, TLRs, defensins) 24 h after *S.* Typhimurium challenge.^27^ More recent work used washed, closed jejunal loops in pigs to investigate host–pathogen interactions in amebiasis and separately assessed hepatic abscess formation after portal-vein or intrahepatic inoculation, enabling study of both intestinal and extraintestinal disease manifestations.^24^ By contrast, the IsoLoop provides a translational swine platform that directly interrogates the host–microbiota–metabolite interface through controlled implantation of exogenous (including human) microbiota into microbiota-depleted ileal loops with intra-animal controls and integrated multi-omics profiling—a combination that distinguishes this study from prior loop models.

By enabling intra-animal, multi-treatment comparisons, the model reduced animal use by 75% by using four loops instead of four pigs for a four-treatment comparison, effectively addressing the “Reduction” principle while maintaining experimental rigor. The surgical design preserved mucosal integrity and host physiology, as evidenced by rapid recovery (feeding within 24 h), minimal distress, and preserved tissue architecture across all loops, aligning with the “Refinement” principle by minimizing postoperative suffering. Additionally, by detaching the loops from the digestive tract, the model bypasses direct animal suffering related to digestion and associated systemic effects, further enhancing refinement by isolating experimental interventions from normal gastrointestinal function. Furthermore, the model's design allows for creating more than four loops, as demonstrated by the 35 cm ileal segment used in our experiments, which could accommodate additional treatments or further reduce animal use depending on the experimental design. This flexibility extends to modifications such as cannulation of loops to the exterior or pexying loops to the abdominal wall, enabling laparoscopic or cannulated inoculations and sample collections for multiple temporal studies, thus enhancing the model's utility for longitudinal microbiome research. Although not a full “Replacement,” the model's ability to replicate human-relevant gut conditions in a swine host offers a translational bridge between *in vitro* systems and whole-animal models, supporting the broader goals of the 3R framework.^7^^,^^23^

In Experiment 1, we investigated the dynamics of host-microbiota interactions by comparing the individual and integrated omics features of the IsoLoops across various treatments and with those of the anastomosed normal ileum, aiming to evaluate the host-microbiota responses to microbial manipulations and to assess how closely these IsoLoops resemble or diverge from the normal ileum of the host pig, thus determining the translational fidelity of the model. A notable finding from Experiment 1 is the re-establishment of microbial diversity in Abx loops by day 5, despite localized antibiotic treatment, thorough pre- and post-antibiotic luminal washing with no culturable bacteria detected in the final wash, and a closed-loop design preventing incoming microbiota. The Shannon and Simpson indices in Abx loops were not significantly different from those in NI, while the Chao1 index remained significantly lower, indicating reduced species richness but largely restored community evenness and diversity, reflecting gut microbiota resilience to antibiotics.^64^ This supports the hypothesis that mucosa-associated microbiota, protected by the mucus layer, may survive antibiotic treatment through physical protection and inherent resistance, proliferating to re-establish a balanced community.^65^^,^^66^ This microbiota resilience highlights the IsoLoop model's potential to study the protective mechanisms of the gut mucosal barrier and its associated microbial communities under controlled conditions. This finding also provides insights into the limitations of oral antibiotic-induced microbiota depletion and HFM inoculation commonly used in whole-animal humanization experiments, which is comparatively less targeted and controlled than the IsoLoop model.^10^^,^^67^ Evidence from human studies showing improved engraftment efficiency with endoscopically delivered intestinal microbiota compared to oral administration supports this argument.^11^ The IsoLoop model's precision in locally isolating and delivering microbial interventions provides a more reliable platform than traditional methods for studying microbiome dynamics and host responses in a controlled environment. Additionally, it supports future methods to improve colonization by targeting region-specific mucosa-associated microbiota. However, while compelling, further comparative studies are needed to quantify these advantages over conventional models.

In Experiment 1, the model effectively demonstrated microbial reconstitution dynamics following antibiotic depletion and HFM transplantation. The diversity trends from Abx to Abx + HFM to Abx + HFM + PH groups, coupled with the restoration of commensal taxa such as *Akkermansia* and *Bifidobacterium*, highlights the model’s capability to capture niche-specific microbial engraftment and ecological shifts.^30^ Functional profiling further revealed partially restored metabolic capabilities, particularly in carbohydrate metabolism, and with *P. hiranonis* enhancing bile acid transformation through enrichment of *bai* genes, even in the absence of bile influx.

A key finding from Experiment 1 is the remarkable transcriptomic recovery in Abx + HFM loops, which achieved a near-normal ileum-like state despite only partial taxonomic and bacterial functional restoration. Transcriptomic analysis revealed that Abx + HFM loops exhibited strong normalization relative to NI, with only 24 DEGs compared to the 596 DEGs observed in Abx loops, with no unique DEGs, indicating a significant reduction in host gene expression dysregulation following HFM transplantation. This recovery was marked by the downregulation of stress-related pathways such as ECM-receptor interaction, focal adhesion, and calcium signaling, suggesting reduced epithelial stress responses and remodeling, while upregulated pathways included bile secretion, steroid hormone biosynthesis, xenobiotic metabolism (cytochrome P450), pentose/glucuronate interconversions, glutathione-mediated antioxidant defense, ABC transporters, and fat/vitamin digestion/absorption, reflecting revitalized epithelial protection and nutrient processing capabilities.

Taxonomically, Abx + HFM loops partially restored microbial diversity, showing no significant differences in richness or evenness compared to Abx loops (Chao1: *p* = 0.08; Shannon: *p* = 0.400; Simpson: *p* = 0.626), but remained distinct from NI with reduced Firmicutes (*p* = 7.31416 × 10⁻⁴). Core microbiome analysis revealed resurgence of beneficial taxa such as *Akkermansia muciniphila* and *Bifidobacterium bifidum*, supporting epithelial restoration, alongside human-colonic taxa (*Bacteroides vulgatus*, *Parabacteroides distasonis*, *Eggerthella lenta*), but dysbiotic taxa (*Enterococcus*, *Streptococcus*) persisted, and native *Lactobacillus* recovery was incomplete, indicating taxonomic restoration lagged behind transcriptomic normalization. Although microbial diversity was not fully restored in Abx + HFM loops, this apparent discrepancy with transcriptomic recovery can be explained by microbial functional redundancy. A previous study showed that germ-free mice colonized with either mouse or zebrafish microbiota exhibited highly similar ileal transcriptional responses despite markedly different microbial communities, highlighting that conserved microbial functions are sufficient to normalize host gene expression. Likewise, in our model, the transplanted community may have reconstituted essential functions needed for host transcriptomic recovery, even in the absence of complete taxonomic restoration.^68^ Bacterial functional profiling showed enriched KEGG orthologs in Abx + HFM loops for antibiotic resistance (K07694), carbohydrate metabolism (K16785, K16787), protein synthesis (K02876), and nutrient transport (K03429), contrasting with stress-response (K05770) and motility (K02557) pathways in Abx loops, reflecting a partial functional shift toward host recovery, though core metabolic functions (e.g., K02970, K03892) remained unrestored compared to NI.

Metabolically, Abx + HFM loops diverged from NI (PCA: PC1 79.7%, PC2 5.6%), with reduced bile acids (e.g., glycodeoxycholic acid), biliverdin, acetic acid, and glucuronic acid due to biliary isolation, and elevated argininic acid and 15(S)-HpETE, indicating nitrogen metabolism shifts and inflammatory tone. Pathway disruptions in bile acid biosynthesis, glutathione metabolism, and phenylalanine/tyrosine metabolism persisted in Abx + HFM versus NI, unlike restored pathways (e.g. pyruvate metabolism) versus Abx. This persistent metabolomic dysregulation suggests that transcriptomic recovery may be driven by specific metabolites or bacterial proteins rather than the full spectrum of gut metabolites, as the limited and selective metabolite recovery in Abx + HFM compared to Abx highlights the lasting impact of Abx-induced dysbiosis on microbial metabolic networks within the 5-d timeframe.

Microbiome-metabolome co-analysis showed fewer correlations in Abx + HFM loops than in Abx versus NI, with *Bacteroides* species (*B. finegoldii*, *B. dorei*) linked to D-fructose and L-valine, and fermentation metabolites (lactic acid, D-ribose) to *E. coli* and *E. faecalis*, but opportunistic pathogens (*Shigella* spp., *E. coli*) correlated with oxidized glutathione, indicating persistent dysbiosis. Collectively, the transcriptomic recovery in Abx + HFM loops, despite incomplete taxonomic, functional, and metabolic restoration, highlights the IsoLoop model's ability to identify specific taxa (*Akkermansia*, *Bifidobacterium*) and functions (carbohydrate metabolism, nutrient transport) as drivers of host recovery, offering insights into microbial therapeutic mechanisms and the model's translational potential.

The increased abundance of bile acid inducible operon genes in Abx + HFM + PH loops underscores *P. hiranonis*'s specificity in enriching secondary bile acid transformation, shaping microbial-metabolite interactions by altering the metabolic environment to favor taxa linked to beneficial metabolites such as taurine and short-chain fatty acids, potentially enhancing gut homeostasis,^36^^,^^38^^,^^40,^^69-71^ Notably, the detection of *P. hiranonis* in the NI group, likely reflecting its natural presence in the porcine gut, underscores the importance of baseline microbial profiling in such models to accurately interpret introduced microbial effects. Additionally, bile acid-converting bacteria possess unique bile-independent metabolic pathways, such as Stickland metabolism for amino acid fermentation (e.g., glycine, proline), which may further contribute to its metabolic versatility and influence on microbial-metabolite networks within the gut ecosystem.^37^ The general depletion of *Bifidobacterium* and *Lactobacillus* in Abx + HFM + PH loops may indicate competitive exclusion by *P. hiranonis* in a bile-depleted environment, as these bacteria share bile resistance linked to glycolysis activation in common.^72^

Despite taxonomic differences, particularly the persistent depletion of *Bifidobacterium* and *Lactobacillus* in Abx + HFM + PH loops, functional profiles of major pathways in these loops showed greater similarity to NI than Abx or Abx + HFM loops. A notable contrast emerged between transcriptional and metabolic resilience in the Abx + HFM + PH group. Although microbial communities remained structurally distinct from NI, with lower diversity and altered taxonomic composition, their functional profiles—particularly metabolic pathways such as glycolysis, arginine biosynthesis, and taurine/hypotaurine metabolism—converged toward NI-like states in major functional pathways. This suggests *P. hiranonis* contributes to a metabolically resilient ecosystem, independent of bile influx, highlighting the model's ability to dissect the interplay between microbial structure and function. This observation is reinforced by more balanced and less pronounced microbiome-metabolome correlation differences between Abx + HFM + PH and NI compared to the extensive differences between Abx versus NI and Abx + HFM versus NI, indicating greater metabolic readjustment with *P. hiranonis*. Additionally, despite the general depletion of *Bifidobacterium* and *Lactobacillus*, enriched correlations between beneficial *Lactobacillus* species (*L. delbrueckii*, *L. fermentum*) and metabolites such as L-valine, glutamic acid, and D-lactic acid in Abx + HFM + PH versus NI, absent in other comparisons, alongside the absence of Enterobacteriaceae-oxidized glutathione correlations, unlike in Abx and Abx + HFM loops, suggest *P. hiranonis* enhances beneficial metabolic networks while reducing virulence-associated pathways with opportunistic flora, further supporting its role in functional restoration.^73^^,^^74^ Moreover, upregulation of PI3K-Akt, MAPK, and mucin-type O-glycan biosynthesis, and ECM-receptor interaction pathways in Abx + HFM + PH loops indicates epithelial adaptation to a stress-tolerant but metabolically diverse microbial community.^75^ These pathways, supporting cell adhesion, migration, and tissue remodeling, likely reflect a compensatory response to the altered microbial landscape, enabling the host to maintain barrier integrity despite taxonomic shifts.^76^ This adaptation underscores *P. hiranonis*'s role in promoting epithelial resilience in a dysbiotic context despite a bile-limited environment.

The significant increase in two conjugated secondary bile acids—lithocholate 3-O-glucuronide and ursodeoxycholic acid 3-sulfate—in Abx + HFM + PH loops versus NI (*p* < 0.05), despite disconnection from biliary inflow, is likely due to microbial transformation/recycling of available substrates. In this setting, *P. hiranonis*—which harbors bile-salt hydrolase (BSH) and the bai operon—can deconjugate and 7α-dehydroxylate residual host-derived primary bile acids retained at the time of segment isolation (despite thorough washing), and may also act on trace taurocholic acid carried with the *P. hiranonis* inoculum, yielding LCA/DCA; members of the HFM provide 7β-HSDH activity that can epimerize CDCA to UDCA.^31^ The resulting microbial products are likely and subsequently conjugated by the intestinal epithelium (e.g., LCA−3-O-glucuronide, UDCA−3-sulfate), matching the metabolites detected here.^77^^,^^78^ While we cannot exclude that sterol-derived intermediates from epithelial turnover or microbial metabolism might provide limited alternative precursors under bile-deficient conditions, classical *de novo* bile-acid biosynthesis by bacteria is unlikely. Notably, these shifts were detectable despite incomplete microbial colonization, underscoring the IsoLoop model’s sensitivity and spatial precision for resolving loop-localized bile-acid metabolism and for testing targeted microbiome–bile-acid interventions.

In Experiment 2, the IsoLoop model effectively replicated CDI dynamics in group 2a, with infected loops showing epithelial damage, crypt necrosis, ulceration, and abscess formation, alongside robust upregulation of inflammatory pathways, including MAPK, TNF, PI3K-Akt, and chemokine signaling, recapitulating *C. difficile* toxin-mediated pathology.^62^ These findings validate the utility of swine IsoLoop model for studying infectious diseases such as CDI, where host bile acid dynamics are crucial, overcoming limitations of existing models for CDI pathology and microbiota interactions.^79-83^ For example, mice provide an established model for antibiotic-induced CDI; however, their bile acid profile confounds the translational relevance of *C. difficile*-bile acid interplay in humans. The primary bile acid, muricholic acid, in mice inhibits *C. difficile*, whereas cholic acid, its human counterpart, facilitates *C. difficile* spore germination and colonization.^42^ Similarly, pigs have hyocholic acid as their primary bile acid, unlike humans, and the IsoLoop model’s isolation of native biliary inflow enhances its translational advantage.^44^^,^^84^ Tissue-level pathology studies using *ex vivo* and *in vivo* ligated ileal loops are limited by ultrashort experimental duration, animal welfare concerns, high animal numbers *in vivo*, lack of systemic and neurovascular components *ex vivo*, and confounding host-specific bile acid profiles, as described in the introduction.^21^^,^^22^ Therefore, the IsoLoop model overcomes these limitations by enabling precise, region-specific microbial interventions with controlled bile acid dynamics, reducing animal use through intra-animal comparisons, and maintaining systemic host responses, thus providing a robust platform for translational CDI research.

Despite its strengths, the IsoLoop model has limitations. The secondary complications observed in the initial stages of Experiment 1 (HFM inoculation) highlight potential risks associated with microbial introductions, with three pigs developing septic peritonitis and one experiencing jejunal infarction, likely due to procedural factors involving the introduction of extraneous microbiota to a surgical site, necessitating meticulous care during inoculation. Experiment 2 groups 2a, 2b, and 2c demonstrated improved surgical reliability (100% success, no complications). The short-term nature of the experiments (5 d) limits insights into long-term microbial engraftment and host adaptation; however, animals were stable on day 5, and extending the duration is feasible since the digestive tract is isolated from the experiment, and the loops remain stable. Additionally, while the model reduces animal use, the surgical complexity requires skilled personnel and careful perioperative management to ensure animal welfare, aligning with the Refinement principle. These challenges can be overcome by optimizing procedural techniques for microbial inoculation and extending experimental timelines to further strengthen the model’s utility for comprehensive gut host-microbiota studies.

In conclusion, the IsoLoop model provides a robust, ethical platform for studying gut-microbiota interactions, offering precise control over microbial interventions and translational insights into gut metabolite interactions, microbial therapeutics, and enteric infections. Its ability to advance the 3 R principles while delivering detailed multi-omics data positions it as a valuable tool for future research into gut health and disease, particularly for exploring the therapeutic potential of bacteria such as *P. hiranonis* in managing dysbiosis-related conditions.

**Table 1.** Multi-omic characteristics of IsoLoops and normal ileum: this table summarizes key microbiota, transcriptomic, and metabolomic differences across the anastomosed normal ileum (NI) and IsoLoop model treatment groups: microbiota depleted (Abx), Abx with human fecal microbiota (Abx + HFM), and Abx with human fecal microbiota plus *P. hiranonis* (Abx + HFM + PH) at day 5 post-treatment. Data are derived from shotgun metagenomic sequencing, RNA-seq, untargeted metabolomics, and histopathological/immunohistochemical (IHC) analyzes. Microbial diversity is assessed using Chao1 (richness), Shannon, and Simpson (evenness) indices. Core microbiota highlights dominant taxa at the species level (>20% prevalence, >0.01% abundance). Functional pathways are based on KEGG orthologs. Transcriptomic profiles report differentially expressed genes (DEGs, Q < 0.05) and enriched KEGG pathways. Metabolomic profiles identify key metabolites and pathways (*p *< 0.05, |fold change| ≥ 1.5). Histopathology/IHC evaluates mucosal integrity and marker expression (E-cadherin, Ki−67, pIgR, lysozyme, *β*-catenin). Statistical significance is noted where applicable (*p* < 0.05).

Parameter	Normal ileum (NI)	Abx (microbiota-depleted)	Abx + HFM (human fecal microbiota)	Abx + HFM + PH (HFM + *P. hiranonis*)
Microbial diversity	High richness (Chao1), evenness (Shannon, Simpson); stable community structure.	Reduced richness (Chao1, *p* = 0.025), stable evenness; dysbiotic community re-established by day 5.	Partial restoration of diversity; no significant difference vs. Abx (Chao1: *p* = 0.08).	Lower richness than NI (Chao1, *p* < 0.05), stable evenness; selective suppression by *P. hiranonis.*
Core microbiota	Dominated by Firmicutes (*Lactobacillus* spp.), Actinobacteria (*Bifidobacterium* spp.).	Loss of *Lactobacillus*, *Bifidobacterium*; enriched *Bacteroides* spp., opportunistic pathogens (e.g., *Shigella*, *E. coli*).	Hybrid community: human taxa (*Akkermansia, Bifidobacterium, Bacteroides*), residual porcine commensals.	Bacteroides dominance, enriched *Akkermansia*; suppressed *Lactobacillus, Bifidobacterium*.
Microbial functional pathways	Enriched in nutrient processing, lactose permease), fermentation.	Stress-response, glutathione metabolism), carbohydrate metabolism; depleted core metabolic functions.	Antibiotic resistance, carbohydrate metabolism, mucolytic pathways; partial restoration.	Biofilm formation, nucleotide salvage; NI-like metabolic convergence.
Transcriptomic profile	Baseline; metabolic and immune homeostasis (e.g., cytochrome P450, cytokine signaling).	596 total DEGs (79 unique); upregulated focal adhesion, MAPK; downregulated metabolic, immune pathways.	24 total DEGs (0 unique); near-normalization, upregulated bile secretion, antioxidant defense, nutrient transport.	1129 total DEGs (610 unique); dramatic transcriptomic shift, upregulated PI3K-Akt, mucin-type O-glycan biosynthesis, ECM-receptor interaction; downregulated bile secretion.
Metabolomic profile	Balanced bile acids, SCFAs (e.g., acetic acid), biliverdin, glucuronic acid.	Reduced bile acids, SCFAs; elevated argininic acid, 15(S)-HpETE (inflammatory).	Reduced bile acids, biliverdin; elevated argininic acid, 15(S)-HpETE; partial pyruvate metabolism recovery.	Elevated specific secondary bile acids (lithocholate 3-O-glucuronide, ursodeoxycholic acid 3-sulfate); reduced inflammation.
Key correlations	*Lactobacillus, Bifidobacterium* correlated with SCFAs, gut barrier function.	*Bacteroides* correlated with oxidized glutathione, dysbiotic metabolites.	*Bacteroides, Akkermansia* correlated with D-fructose, L-valine; persistent dysbiotic correlations.	*P. hiranonis* drives metabolic shift, *Lactobacillus* correlated with L-valine, glutamic acid; reduced dysbiotic correlations.
Histopathology/IHC	Intact mucosa, normal E-cadherin, Ki-67, pIgR, lysozyme, *β*-catenin expression.	Preserved mucosa, trend toward reduced E-cadherin, Ki-67; increased *β*-catenin.	Preserved mucosa, trends towards restored E-cadherin, Ki-67, and increased *β*-catenin.	Preserved mucosa, trend toward restored E-cadherin, Ki-67; enhanced epithelial repair pathways.

## Supplementary Material

Supplementary material Table S1. Taxonomic composition of human fecal microbiota mixture (HFM). This table provides a detailed overview of the taxonomic composition of the HFM used for transplantation in the IsoLoop model experiment 1. The table lists the 20 most abundant bacterial families, their top six genera, and the 10 most abundant species, expressed as relative abundance percentages. Data were derived from shotgun metagenomic sequencing of fecal samples collected from six healthy adult donors, processed under anaerobic conditions, and analyzed using a curated reference database (Diversigen pipeline). Families are ordered by abundance, with genera and species ranked within each family. “Other” denotes aggregated taxa below the specified threshold or unclassified at the respective taxonomic level.
Figure S1. Immunohistochemical analysis of epithelial and immune markers in IsoLoops and normal ileum: immunohistochemical staining of ileal sections from anastomosed normal ileum (NI), microbiota-depleted loop (Abx), microbiota-depleted loop with human fecal microbiota (Abx+HFM), and microbiota-depleted loop with human fecal microbiota and *P. hiranonis* (Abx+HFM+PH) using validated antibodies for β-catenin, E-cadherin, cytokeratin peptide 18 (CK18), polymeric immunoglobulin receptor (pIgR), Ki-67, and lysozyme.
Figure S2. Additional taxonomic compositional differences in IsoLoops comparing Abx vs. NI and Abx+HFM vs NI: Boxplots display log-transformed read counts of (A) key bacterial families in Abx vs. NI and their p-values and (B) key bacterial phyla Abx+HFM vs. NI with their p-values.
Figure S3. Species-level pair-wise microbial correlation patterns across IsoLoop comparisons: (A–E) Pattern search analysis identifies the top 25 microbial species correlated with each comparison: (A) Abx vs. NI, (B) Abx+HFM vs. Abx, (C) Abx+HFM vs. NI, (D) Abx+HFM+PH vs. Abx+HFM, and (E) Abx+HFM+PH vs. NI. Heatmaps show correlation strengths, highlighting species driving microbial community differences.
Figure S4. Differentially abundant taxa in microbiota-depleted IsoLoops versus normal ileum: (A) Single-factor analysis plot depicting differentially abundant species across bacterial phyla in Abx vs. NI, with each point representing a taxon, plotted by phylum (shaded background) on the x-axis and −log₁₀(*p*) on the y-axis. Point size reflects logarithmic abundance, upward triangles indicate positive fold changes, and downward triangles indicate negative fold changes. (B) Multiple regression analysis of taxonomic features at the species level associated with Abx vs. NI, with blue circles marking five significantly enriched species (*Bacteroides ovatus*,*B. xylanisolvens*, *Lactobacillus delbrueckii*, *Lactobacillus sp.* HMSC08B12, *B. dorei*; adjusted *p *< 0.05) and grey dots indicating non-significant species. Dashed lines denote *p *= 0.05 thresholds (−log₁₀ = 1.3).
Figure S5. Random forest classification of IsoLoops by bacterial genera: (A–E) Random forest analysis identifies key bacterial genera distinguishing specific treatment comparisons: (A) Abx vs. NI, (B) Abx+HFM vs. Abx, (C) Abx+HFM vs. NI, (D) Abx+HFM+PH vs. Abx+HFM, and (E) Abx+HFM+PH vs. NI. Genera are ranked by Mean Decrease Accuracy (MDA) scores, with bar plots showing their discriminatory power.
Figure S6. Functional KEGG ortholog patterns in IsoLoops: (A–E) Pattern search analysis of KEGG ortholog abundance identifies functional shifts in: (A) Abx vs. NI, (B) Abx+HFM vs. Abx, (C) Abx+HFM vs. NI, (D) Abx+HFM+PH vs. Abx+HFM, and (E) Abx+HFM+PH vs. NI.
Figure S7. Random forest classification of KEGG orthologs in IsoLoop comparisons: (A–E) Random forest analysis identifies key KEGG orthologs (KOs) distinguishing: (A) Abx vs. NI, (B) Abx+HFM vs. Abx, (C) Abx+HFM vs. NI, (D) Abx+HFM+PH vs. Abx+HFM, and (E) Abx+HFM+PH vs. NI. Heatmaps show relative KO abundance, ranked by Mean Decrease Accuracy (MDA) scores.
Figure S8. Metabolomic changes in microbiota-depleted IsoLoops versus normal ileum (nc and pc modes): (A) Heatmap displays top differentially abundant metabolites in Abx vs. NI (nc mode), highlighting distinct metabolic signatures. (B) Heatmap shows metabolite differences in Abx vs. NI (pc mode).
Figure S10. Metabolomic profiles of humanized IsoLoops versus microbiota-depleted loops (nc mode): **(**A) Principal component analysis (PCA) plot illustrates group-specific clustering. (B) Volcano plot depicts a smaller number of metabolites with significant fold changes (red: upregulated, blue: downregulated; p < 0.05). (C) Pathway enrichment analysis identifies metabolic pathways altered by HFM introduction.
Figure S11. Metabolomic remodeling in humanized IsoLoops versus normal ileum (nc mode): (A) Heatmap shows clustering of differentially abundant metabolites in Abx+HFM vs. NI. (B) Pathway enrichment analysis identifies metabolic pathways altered between Abx+HFM and NI.
Figure S12. Relative abundance of metabolites in humanized IsoLoops versus normal ileum: (A) Bar plots show relative abundance of bile acid-related metabolites in Abx+HFM vs. NI. (B) Bar plots display amino acid-associated metabolites. (C) Bar plots illustrate metabolites linked to inflammatory signaling (15(S)-HpETE), microbial fermentation (acetic acid, β-D-lactose), antioxidant activity (biliverdin IX), and detoxification (glucuronic acid), with statistical significance indicated: * *p* < 0.05; ** *p* < 0.01; *** *p* < 0.001; **** *p* < 0.0001.
Figure S13. Metabolomic profiling of P. hiranonis-enriched IsoLoops versus humanized loops (nc mode): (A) PCA plot depicting lack of robust clustering between Abx+HFM+PH vs. Abx+HFM loops. (B) Volcano plot depicts a small number of metabolites with significant fold changes (red: upregulated, blue: downregulated; p < 0.05). (C) Enrichment pathway plot identifies metabolic pathways impacted by P. hiranonis. (D–E) Bar plots show the relative abundance of two key metabolites altered by P. hiranonis.
Figure S14. Metabolomic profiling of P. hiranonis-enriched IsoLoops versus normal ileum (nc mode): (A) Clustering heatmap shows metabolite distribution in Abx+HFM+PH vs. NI. (B) Volcano plot depicts metabolites with significant fold changes (red: upregulated, blue: downregulated; p < 0.05). (C) Enrichment pathway plot identifies metabolic pathways altered in Abx+HFM+PH vs. NI.
Figure S15. Integrated multi-omics analysis of P. hiranonis-enriched IsoLoops versus humanized loops: (A) Joint pathway analysis integrates transcriptomic and metabolomic data to identify significantly impacted pathways in Abx+HFM+PH vs. Abx+HFM. (B) Microbiome-metabolome correlation heatmap highlights associations between dominant bacterial species and key metabolites in Abx+HFM+PH vs. Abx+HFM.
Figure S16. Integrated multi-omics analysis of humanized IsoLoops versus normal ileum: (A) Joint pathway analysis integrates transcriptomic and metabolomic data to identify significantly impacted pathways in Abx+HFM vs. NI. (B) Microbiome-metabolome correlation heatmap highlights associations between dominant bacterial species and key metabolites in Abx+HFM vs. NI.

## Data Availability

The data supporting the findings of this study are openly available on Zenodo at DOI: 10.5281/zenodo.15547660. Sequence data are available in the NCBI GenBank under BioProject ID: PRJNA1269114.
