# Supplementary material for: The swine IsoLoop model of the gut host-microbiota interface enables intra-animal treatment comparisons to advance 3R principles
Source: Gut Microbes. 2025 Oct 25;17(1):2568706. doi: 10.1080/19490976.2025.2568706 (PMC12562777; doi:10.1080/19490976.2025.2568706)
Supplement: Supplementary material — Table S1. Taxonomic composition of human fecal microbiota mixture (HFM). This table provides a detailed overview of the taxonomic composition of the HFM used for transplantation in the IsoLoop model experiment 1. The table lists the 20 most abundant bacterial families, their top six genera, and the 10 most abundant species, expressed as relative abundance percentages. Data were derived from shotgun metagenomic sequencing of fecal samples collected from six healthy adult donors, processed under anaerobic conditions, and analyzed using a curated reference database (Diversigen pipeline). Families are ordered by abundance, with genera and species ranked within each family. “Other” denotes aggregated taxa below the specified threshold or unclassified at the respective taxonomic level. Figure S1. Immunohistochemical analysis of epithelial and immune markers in IsoLoops and normal ileum: immunohistochemical staining of ileal sections from anastomosed normal ileum (NI), microbiota-depleted loop (Abx), microbiota-depleted loop with human fecal microbiota (Abx+HFM), and microbiota-depleted loop with human fecal microbiota and P. hiranonis (Abx+HFM+PH) using validated antibodies for β-catenin, E-cadherin, cytokeratin peptide 18 (CK18), polymeric immunoglobulin receptor (pIgR), Ki-67, and lysozyme. Figure S2. Additional taxonomic compositional differences in IsoLoops comparing Abx vs. NI and Abx+HFM vs NI: Boxplots display log-transformed read counts of (A) key bacterial families in Abx vs. NI and their p-values and (B) key bacterial phyla Abx+HFM vs. NI with their p-values. Figure S3. Species-level pair-wise microbial correlation patterns across IsoLoop comparisons: (A–E) Pattern search analysis identifies the top 25 microbial species correlated with each comparison: (A) Abx vs. NI, (B) Abx+HFM vs. Abx, (C) Abx+HFM vs. NI, (D) Abx+HFM+PH vs. Abx+HFM, and (E) Abx+HFM+PH vs. NI. Heatmaps show correlation strengths, highlighting species driving microbial community differences. Fi [file KGMI_A_2568706_SM5576.docx]

**Supplementary Table Legend**

**Table S1: Taxonomic composition of human fecal microbiota mixture (HFM)**: This table provides a detailed overview of the taxonomic composition of the HFM used for transplantation in the IsoLoop Model experiment 1. The table lists the 20 most abundant bacterial families, their top six genera, and the 10 most abundant species, expressed as relative abundance percentages. Data were derived from shotgun metagenomic sequencing of fecal samples collected from six healthy adult donors, processed under anaerobic conditions, and analyzed using a curated reference database (Diversigen pipeline). Families are ordered by abundance, with genera and species ranked within each family. "Other" denotes aggregated taxa below the specified threshold or unclassified at the respective taxonomic level.

**Supplementary Figure Legends**

**Figure S1. Immunohistochemical analysis of epithelial and immune markers in IsoLoops and normal ileum:** Immunohistochemical staining of ileal sections from anastomosed normal ileum (NI), microbiota-depleted loop (Abx), microbiota-depleted loop with human fecal microbiota (Abx+HFM), and microbiota-depleted loop with human fecal microbiota and *P. hiranonis* (Abx+HFM+PH) using validated antibodies for β-catenin, E-cadherin, cytokeratin peptide 18 (CK18), polymeric immunoglobulin receptor (pIgR), Ki-67, and lysozyme.

**Figure S2. Additional taxonomic compositional differences in IsoLoops comparing Abx vs. NI and Abx+HFM vs NI:** Boxplots display log-transformed read counts of (A) key bacterial families in Abx vs. NI and their p-values and (B) key bacterial phyla Abx+HFM vs. NI with their p-values.

**Figure S3. Species-level pair-wise microbial correlation patterns across IsoLoop comparisons:** (A–E) Pattern search analysis identifies the top 25 microbial species correlated with each comparison: (A) Abx vs. NI, (B) Abx+HFM vs. Abx, (C) Abx+HFM vs. NI, (D) Abx+HFM+PH vs. Abx+HFM, and (E) Abx+HFM+PH vs. NI. Heatmaps show correlation strengths, highlighting species driving microbial community differences.

**Figure S4. Differentially abundant taxa in microbiota-depleted IsoLoops versus normal ileum:** (A) Single-factor analysis plot depicting differentially abundant species across bacterial phyla in Abx vs. NI, with each point representing a taxon, plotted by phylum (shaded background) on the x-axis and –log₁₀(p) on the y-axis. Point size reflects logarithmic abundance, upward triangles indicate positive fold changes, and downward triangles indicate negative fold changes. (B) Multiple regression analysis of taxonomic features at the species level associated with Abx vs. NI, with blue circles marking five significantly enriched species (*Bacteroides ovatus*, *B. xylanisolvens*, *Lactobacillus delbrueckii*, *Lactobacillus* sp. HMSC08B12, *B. dorei*; adjusted p < 0.05) and grey dots indicating non-significant species. Dashed lines denote p = 0.05 thresholds (–log₁₀ = 1.3).

**Figure S5. Random forest classification of IsoLoops by bacterial genera:** (A–E) Random forest analysis identifies key bacterial genera distinguishing specific treatment comparisons: (A) Abx vs. NI, (B) Abx+HFM vs. Abx, (C) Abx+HFM vs. NI, (D) Abx+HFM+PH vs. Abx+HFM, and (E) Abx+HFM+PH vs. NI. Genera are ranked by Mean Decrease Accuracy (MDA) scores, with bar plots showing their discriminatory power.

**Figure S6. Functional KEGG ortholog patterns in IsoLoops:** (A–E) Pattern search analysis of KEGG ortholog abundance identifies functional shifts in: (A) Abx vs. NI, (B) Abx+HFM vs. Abx, (C) Abx+HFM vs. NI, (D) Abx+HFM+PH vs. Abx+HFM, and (E) Abx+HFM+PH vs. NI.

**Figure S7. Random forest classification of KEGG orthologs in IsoLoop comparisons:** (A–E) Random forest analysis identifies key KEGG orthologs (KOs) distinguishing: (A) Abx vs. NI, (B) Abx+HFM vs. Abx, (C) Abx+HFM vs. NI, (D) Abx+HFM+PH vs. Abx+HFM, and (E) Abx+HFM+PH vs. NI. Heatmaps show relative KO abundance, ranked by Mean Decrease Accuracy (MDA) scores.

**Figure S8. Metabolomic changes in microbiota-depleted IsoLoops versus normal ileum (nc and pc modes):** (A) Heatmap displays top differentially abundant metabolites in Abx vs. NI (nc mode), highlighting distinct metabolic signatures. (B) Heatmap shows metabolite differences in Abx vs. NI (pc mode).

**Figure S9. Metabolomic changes in microbiota-depleted IsoLoops versus normal ileum (nhilic and philic modes):** (A) Heatmap displays top differentially abundant metabolites in Abx vs. NI (nhilic mode), highlighting metabolic shifts. (B) Heatmap displays top differentially abundant metabolites in Abx vs. NI (philic mode).

**Figure S10. Metabolomic profiles of humanized IsoLoops versus microbiota-depleted loops (nc mode): (**A) Principal component analysis (PCA) plot illustrates group-specific clustering. (B) Volcano plot depicts a smaller number of metabolites with significant fold changes (red: upregulated, blue: downregulated; p < 0.05). (C) Pathway enrichment analysis identifies metabolic pathways altered by HFM introduction.

**Figure S11. Metabolomic remodeling in humanized IsoLoops versus normal ileum (nc mode):** (A) Heatmap shows clustering of differentially abundant metabolites in Abx+HFM vs. NI. (B) Pathway enrichment analysis identifies metabolic pathways altered between Abx+HFM and NI.

**Figure S12. Relative abundance of metabolites in humanized IsoLoops versus normal ileum:** (A) Bar plots show relative abundance of bile acid-related metabolites in Abx+HFM vs. NI. (B) Bar plots display amino acid-associated metabolites. (C) Bar plots illustrate metabolites linked to inflammatory signaling (15(S)-HpETE), microbial fermentation (acetic acid, β-D-lactose), antioxidant activity (biliverdin IX), and detoxification (glucuronic acid), with statistical significance indicated: * p < 0.05; ** p < 0.01; *** p < 0.001; **** p < 0.0001.

**Figure S13. Metabolomic profiling of *P. hiranonis*-enriched IsoLoops versus humanized loops (nc mode):** (A) PCA plot depicting lack of robust clustering between Abx+HFM+PH vs. Abx+HFM loops. (B) Volcano plot depicts a small number of metabolites with significant fold changes (red: upregulated, blue: downregulated; p < 0.05). (C) Enrichment pathway plot identifies metabolic pathways impacted by *P. hiranonis*. (D–E) Bar plots show the relative abundance of two key metabolites altered by *P. hiranonis*.

**Figure S14. Metabolomic profiling of *P. hiranonis*-enriched IsoLoops versus normal ileum (nc mode):** (A) Clustering heatmap shows metabolite distribution in Abx+HFM+PH vs. NI. (B) Volcano plot depicts metabolites with significant fold changes (red: upregulated, blue: downregulated; p < 0.05). (C) Enrichment pathway plot identifies metabolic pathways altered in Abx+HFM+PH vs. NI.

**Figure S15. Integrated multi-omics analysis of *P. hiranonis*-enriched IsoLoops versus humanized loops:** (A) Joint pathway analysis integrates transcriptomic and metabolomic data to identify significantly impacted pathways in Abx+HFM+PH vs. Abx+HFM. (B) Microbiome-metabolome correlation heatmap highlights associations between dominant bacterial species and key metabolites in Abx+HFM+PH vs. Abx+HFM.

**Figure S16. Integrated multi-omics analysis of humanized IsoLoops versus normal ileum:** (A) Joint pathway analysis integrates transcriptomic and metabolomic data to identify significantly impacted pathways in Abx+HFM vs. NI. (B) Microbiome-metabolome correlation heatmap highlights associations between dominant bacterial species and key metabolites in Abx+HFM vs. NI.

**Table S1**

| **Family**  **(20 most abundant (*%*))** | **Top Genera**  **(6 most abundant (*%*))** | **Top Species**  **(10 most abundant (*%*))** |
| --- | --- | --- |
| Lachnospiraceae (*29.25%*) | *Blautia* (*11.98%*)*, Other* (*7.70%*)*, Fusicatenibacter* (*2.61%*)*, Dorea* (*2.14%*)*, Roseburia* (*1.69%*)*, Coprococcus* (*1.38%*) | *Other* (*8.03%*)*, [Eubacterium] rectale* (*6.34%*)*, Fusicatenibacter saccharivorans* (*2.61%*)*, Blautia obeum* (*2.02%*)*, Dorea longicatena* (*1.87%*)*, [Ruminococcus] torques* (*1.28%*)*, Blautia wexlerae* (*1.24%*)*, Roseburia faecis* (*1.02%*)*, Coprococcus eutactus* (*0.94%*)*, Anaerostipes hadrus* (*0.86%*) |
| Prevotellaceae (*15.05%*) | *Prevotella* (*13.51%*)*, Prevotellamassilia* (*1.34%*)*, Other* (*0.12%*)*, Paraprevotella* (*0.06%*)*, Metaprevotella* (*< 0.01%*)*, Alloprevotella* (*< 0.01%*) | *Prevotella copri* (*12.22%*)*, Prevotellamassilia timonensis* (*1.34%*)*, Prevotella sp. Marseille-P4119* (*0.38%*)*, Prevotella lascolaii* (*0.38%*)*, Other* (*0.29%*)*, Prevotella stercorea* (*0.20%*)*, Prevotella intermedia* (*0.05%*)*, Paraprevotella clara* (*0.04%*)*, Prevotella bivia* (*0.04%*)*, Paraprevotella xylaniphila* (*0.02%*) |
| Ruminococcaceae (*12.46%*) | *Faecalibacterium* (*8.84%*)*, Ruminiclostridium* (*1.74%*)*, Ruminococcus* (*1.11%*)*, Gemmiger* (*0.29%*)*, Other* (*0.19%*)*, Neglecta* (*0.10%*) | *Faecalibacterium prausnitzii* (*8.84%*)*, [Eubacterium] siraeum* (*1.59%*)*, Ruminococcus lactaris* (*0.52%*)*, Gemmiger formicilis* (*0.29%*)*, Ruminococcus faecis* (*0.21%*)*, Ruminococcus champanellensis* (*0.20%*)*, [Clostridium] leptum* (*0.14%*)*, Other* (*0.14%*)*, Ruminococcus callidus* (*0.14%*)*, Neglecta timonensis* (*0.10%*) |
| Bifidobacteriaceae (*10.82%*) | *Bifidobacterium* (*10.81%*)*, Scardovia* (*< 0.01%*) | *Bifidobacterium adolescentis* (*5.35%*)*, Bifidobacterium longum* (*2.46%*)*, Other* (*1.30%*)*, Bifidobacterium pseudocatenulatum* (*0.44%*)*, Bifidobacterium catenulatum* (*0.38%*)*, Bifidobacterium kashiwanohense* (*0.28%*)*, Bifidobacterium bifidum* (*0.28%*)*, Bifidobacterium angulatum* (*0.22%*)*, Bifidobacterium breve* (*0.05%*)*, Bifidobacterium sp. 12 1 47BFAA* (*0.03%*) |
| Other (*9.77%*) | *Other* (*9.32%*)*, Monoglobus* (*0.23%*)*, Intestinimonas* (*0.13%*)*, Flavonifractor* (*0.08%*)*, Pseudoflavonifractor* (*0.02%*)*, Levyella* (*< 0.01%*) | *Other* (*8.80%*)*, Clostridiales bacterium KLE1615* (*0.27%*)*, Monoglobus pectinilyticus* (*0.23%*)*, bacterium OL-1* (*0.17%*)*, Intestinimonas butyriciproducens* (*0.12%*)*, Flavonifractor plautii* (*0.08%*)*, Burkholderiales bacterium 1 1 47* (*0.03%*)*, bacterium LF-3* (*0.02%*)*, [Bacteroides] pectinophilus* (*0.01%*)*, Pseudoflavonifractor capillosus* (*0.01%*) |
| Bacteroidaceae (*6.82%*) | *Bacteroides* (*6.71%*)*, Other* (*0.11%*)*, Mediterranea* (*< 0.01%*) | *Other* (*4.78%*)*, Bacteroides uniformis* (*0.57%*)*, Bacteroides vulgatus* (*0.18%*)*, Bacteroides dorei* (*0.18%*)*, Bacteroides faecis* (*0.15%*)*, Bacteroides ovatus* (*0.11%*)*, Bacteroides thetaiotaomicron* (*0.10%*)*, Bacteroides stercoris* (*0.10%*)*, Bacteroides salyersiae* (*0.07%*)*, Bacteroides caccae* (*0.07%*) |
| Lactobacillaceae (*2.31%*) | *Lactobacillus* (*2.31%*)*, Sharpea* (*< 0.01%*)*, Other* (*< 0.01%*)*, Pediococcus* (*< 0.01%*) | *Lactobacillus ruminis* (*2.27%*)*, Lactobacillus rogosae* (*0.02%*)*, Lactobacillus mucosae* (*0.01%*)*, Other* (*< 0.01%*)*, Lactobacillus crispatus* (*< 0.01%*)*, Lactobacillus fermentum* (*< 0.01%*)*, Sharpea azabuensis* (*< 0.01%*)*, Lactobacillus aviarius* (*< 0.01%*)*, Lactobacillus delbrueckii* (*< 0.01%*)*, Lactobacillus agilis* (*< 0.01%*) |
| Eubacteriaceae (*1.77%*) | *Eubacterium* (*1.70%*)*, Other* (*0.06%*)*, Anaerofustis* (*< 0.01%*)*, Intestinibacillus* (*< 0.01%*) | *[Eubacterium] hallii* (*1.09%*)*, [Eubacterium] eligens* (*0.44%*)*, Eubacterium ramulus* (*0.07%*)*, Eubacteriaceae bacterium CHKCI004* (*0.06%*)*, Eubacterium ventriosum* (*0.05%*)*, Eubacterium sp. 3 1 31* (*0.05%*)*, Other* (*< 0.01%*)*, Eubacterium sp. SB2* (*< 0.01%*)*, Anaerofustis stercorihominis* (*< 0.01%*)*, Eubacterium coprostanoligenes* (*< 0.01%*) |
| Streptococcaceae (*1.68%*) | *Streptococcus* (*1.68%*)*, Other* (*< 0.01%*)*, Lactococcus* (*< 0.01%*) | *Other* (*0.70%*)*, Streptococcus thermophilus* (*0.68%*)*, Streptococcus salivarius* (*0.16%*)*, Streptococcus parasanguinis* (*0.02%*)*, Streptococcus sp. HMSC072D03* (*0.02%*)*, Streptococcus sp. HMSC064D12* (*0.01%*)*, Streptococcus sp. HMSC078H12* (*< 0.01%*)*, Streptococcus vestibularis* (*< 0.01%*)*, Streptococcus sp. HMSC10E12* (*< 0.01%*)*, Streptococcus gallolyticus* (*< 0.01%*) |
| Coriobacteriaceae (*1.37%*) | *Collinsella* (*1.32%*)*, Senegalimassilia* (*0.04%*)*, Enorma* (*0.01%*)*, Other* (*< 0.01%*) | *Collinsella aerofaciens* (*0.68%*)*, Other* (*0.36%*)*, Collinsella sp. TF06-26* (*0.16%*)*, Collinsella sp. 4 8 47FAA* (*0.08%*)*, Collinsella bouchesdurhonensis* (*0.04%*)*, Senegalimassilia anaerobia* (*0.04%*)*, Enorma massiliensis* (*0.01%*)*, Collinsella stercoris* (*< 0.01%*)*, Collinsella intestinalis* (*< 0.01%*)*, Collinsella ihuae* (*< 0.01%*) |
| Selenomonadaceae (1.23%) | *Megamonas* (*1.23%*)*, Selenomonas* (*< 0.01%*)*, Mitsuokella* (*< 0.01%*) | *Other* (*0.71%*)*, Megamonas funiformis* (*0.39%*)*, Megamonas rupellensis* (*0.10%*)*, Megamonas sp. Calf98-2* (*0.03%*)*, Megamonas hypermegale* (*< 0.01%*)*, Selenomonas bovis* (*< 0.01%*)*, Mitsuokella jalaludinii* (*< 0.01%*)*, Mitsuokella multacida* (*< 0.01%*) |
| Rikenellaceae (*1.04%*) | *Alistipes* (*1.00%*)*, Other* (*0.03%*)*, Rikenella* (*< 0.01%*)*, Tidjanibacter* (*< 0.01%*) | *Alistipes finegoldii* (*0.30%*)*, Alistipes putredinis* (*0.26%*)*, Alistipes shahii* (*0.21%*)*, Other* (*0.11%*)*, Alistipes obesi* (*0.07%*)*, Alistipes ihumii* (*0.04%*)*, Alistipes senegalensis* (*0.02%*)*, Alistipes indistinctus* (*0.01%*)*, Alistipes onderdonkii* (*< 0.01%*)*, Alistipes sp. AL-1* (*< 0.01%*) |
| Tannerellaceae (*1.03%*) | *Parabacteroides* (*1.03%*)*, Tannerella* (*< 0.01%*)*, Other* (*< 0.01%*) | *Parabacteroides merdae* (*0.64%*)*, Other* (*0.29%*)*, Parabacteroides distasonis* (*0.04%*)*, Parabacteroides johnsonii* (*0.03%*)*, Parabacteroides sp. SN4* (*< 0.01%*)*, Parabacteroides sp. D13* (*< 0.01%*)*, Parabacteroides sp. 20 3* (*< 0.01%*)*, Parabacteroides sp. D26* (*< 0.01%*)*, Parabacteroides gordonii* (*< 0.01%*)*, Parabacteroides sp. 2 1 7* (*< 0.01%*) |
| Veillonellaceae (*0.97%*) | *Dialister* (*0.64%*)*, Megasphaera* (*0.26%*)*, Allisonella* (*0.04%*)*, Veillonella* (*0.03%*)*, Caecibacter* (*< 0.01%*)*, Other* (*< 0.01%*) | *Dialister invisus* (*0.64%*)*, Megasphaera elsdenii* (*0.23%*)*, Allisonella histaminiformans* (*0.04%*)*, Megasphaera cerevisiae* (*0.02%*)*, Veillonella dispar* (*0.01%*)*, Other* (*0.01%*)*, Dialister succinatiphilus* (*< 0.01%*)*, Veillonella atypica* (*< 0.01%*)*, Veillonella parvula* (*< 0.01%*)*, Veillonella tobetsuensis* (*< 0.01%*) |
| Erysipelotrichaceae (*0.86%*) | *Holdemanella* (*0.40%*)*, Catenibacterium* (*0.22%*)*, Candidatus Stoquefichus* (*0.08%*)*, Other* (*0.06%*)*, Coprobacillus* (*0.02%*)*, Faecalitalea* (*0.02%*) | *Holdemanella biformis* (*0.40%*)*, Catenibacterium mitsuokai* (*0.22%*)*, Candidatus Stoquefichus sp. KLE1796* (*0.08%*)*, Other* (*0.05%*)*, Coprobacillus sp. 8 1 38FAA* (*0.01%*)*, Erysipelotrichaceae bacterium 6 1 45* (*0.01%*)*, Faecalitalea cylindroides* (*< 0.01%*)*, Turicibacter sanguinis* (*< 0.01%*)*, Holdemania filiformis* (*< 0.01%*)*, Faecalitalea sp. Marseille-P3755* (*< 0.01%*) |
| Clostridiaceae (*0.55%*) | *Clostridium* (*0.49%*)*, Hungatella* (*0.03%*)*, Mordavella* (*0.02%*)*, Massilioclostridium* (*0.01%*)*, Butyricicoccus* (*< 0.01%*)*, Lactonifactor* (*< 0.01%*) | *Clostridium sp. L2-50* (*0.19%*)*, Clostridium phoceensis* (*0.10%*)*, Clostridium sp. SS2/1* (*0.05%*)*, Clostridium sp. M62/1* (*0.03%*)*, Clostridium sp. AT4* (*0.03%*)*, Hungatella hathewayi* (*0.03%*)*, Clostridium sp. KLE 1755* (*0.02%*)*, Mordavella sp. Marseille-P3756* (*0.02%*)*, Clostridium sp. ATCC BAA-442* (*0.02%*)*, Massilioclostridium coli* (*0.01%*) |
| Akkermansiaceae (*0.44%*) | *Akkermansia* (*0.44%*) | *Akkermansia muciniphila* (*0.43%*)*, Other* (*< 0.01%*)*, Akkermansia glycaniphila* (*< 0.01%*) |
| Eggerthellaceae (*0.41%*) | *Eggerthella* (*0.23%*)*, Adlercreutzia* (*0.12%*)*, Gordonibacter* (*0.04%*)*, Raoultibacter* (*< 0.01%*)*, Other* (*< 0.01%*)*, Enterorhabdus* (*< 0.01%*) | *Other* (*0.15%*)*, Adlercreutzia equolifaciens* (*0.12%*)*, Eggerthella lenta* (*0.05%*)*, Eggerthella sp. 1 3 56FAA* (*0.04%*)*, Gordonibacter pamelaeae* (*0.03%*)*, Gordonibacter urolithinfaciens* (*< 0.01%*)*, Raoultibacter timonensis* (*< 0.01%*)*, Raoultibacter massiliensis* (*< 0.01%*)*, Enterorhabdus caecimuris* (*< 0.01%*)*, Enterorhabdus mucosicola* (*< 0.01%*) |
| Sutterellaceae (*0.38%*) | *Duodenibacillus* (*0.25%*)*, Sutterella* (*0.10%*)*, Parasutterella* (*0.03%*)*, Other* (*< 0.01%*) | *Duodenibacillus massiliensis* (*0.25%*)*, Sutterella wadsworthensis* (*0.09%*)*, Parasutterella excrementihominis* (*0.03%*)*, Other* (*< 0.01%*)*, Sutterella sp. KLE1602* (*< 0.01%*) |
| Atopobiaceae (*0.35%*) | *Olsenella* (*0.35%*)*, Libanicoccus* (*< 0.01%*) | *Olsenella umbonata* (*0.25%*)*, Olsenella sp. KH3B4* (*0.03%*)*, Olsenella scatoligenes* (*0.02%*)*, Olsenella sp. kh2p3* (*0.02%*)*, Other* (*0.02%*)*, Olsenella provencensis* (*< 0.01%*)*, Libanicoccus massiliensis* (*< 0.01%*)*, Olsenella sp. oral taxon 807* (*< 0.01%*) |

**Figure S1**

**
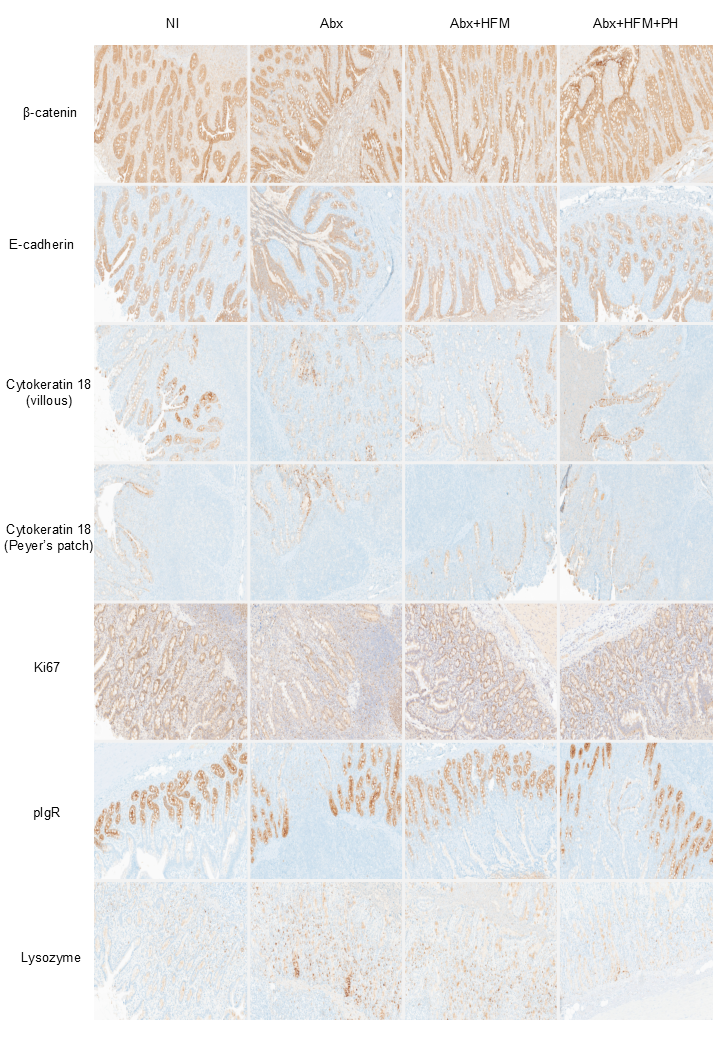
**

**Figure S2**

**
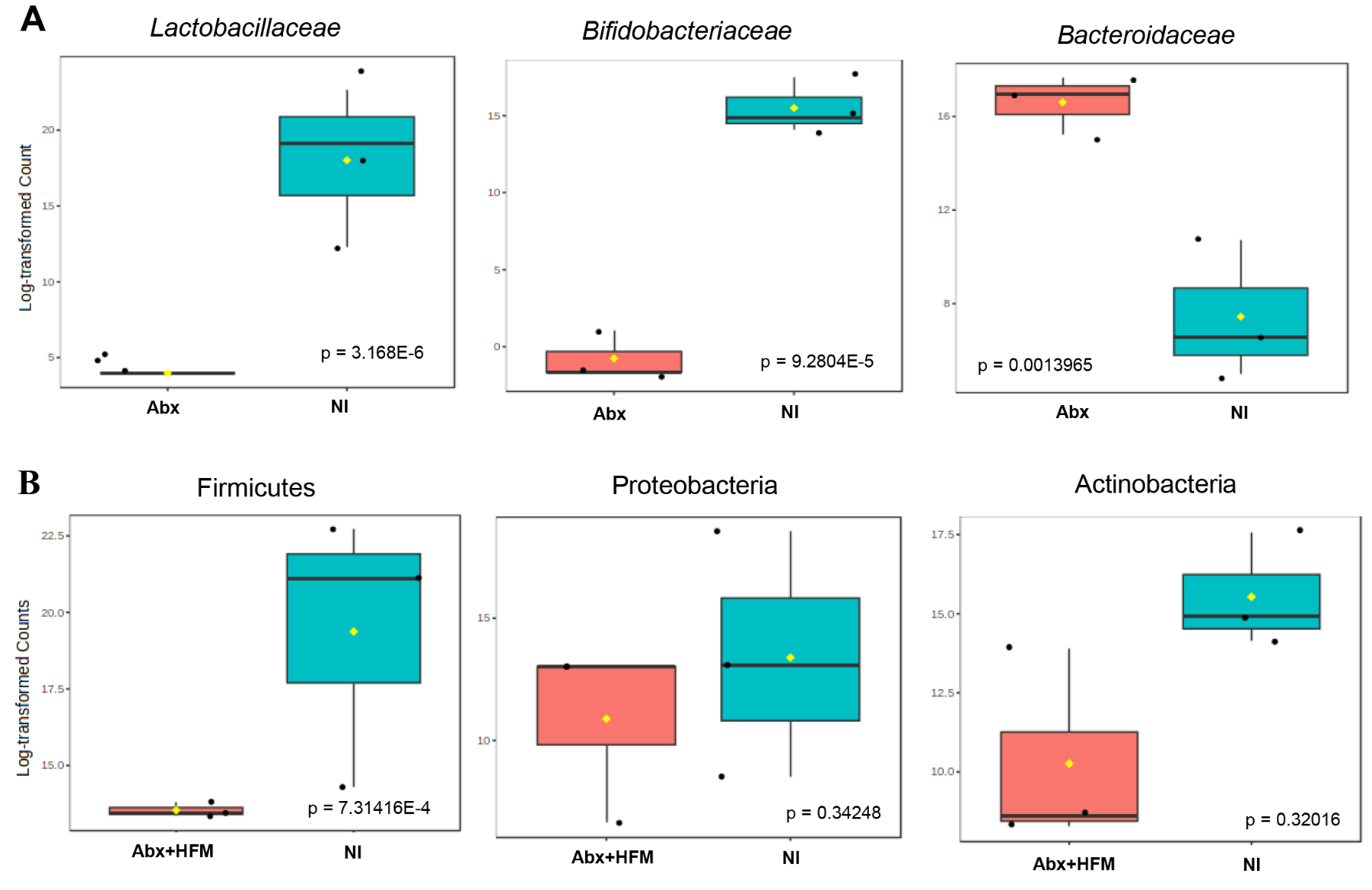
**

**Figure S3**

**
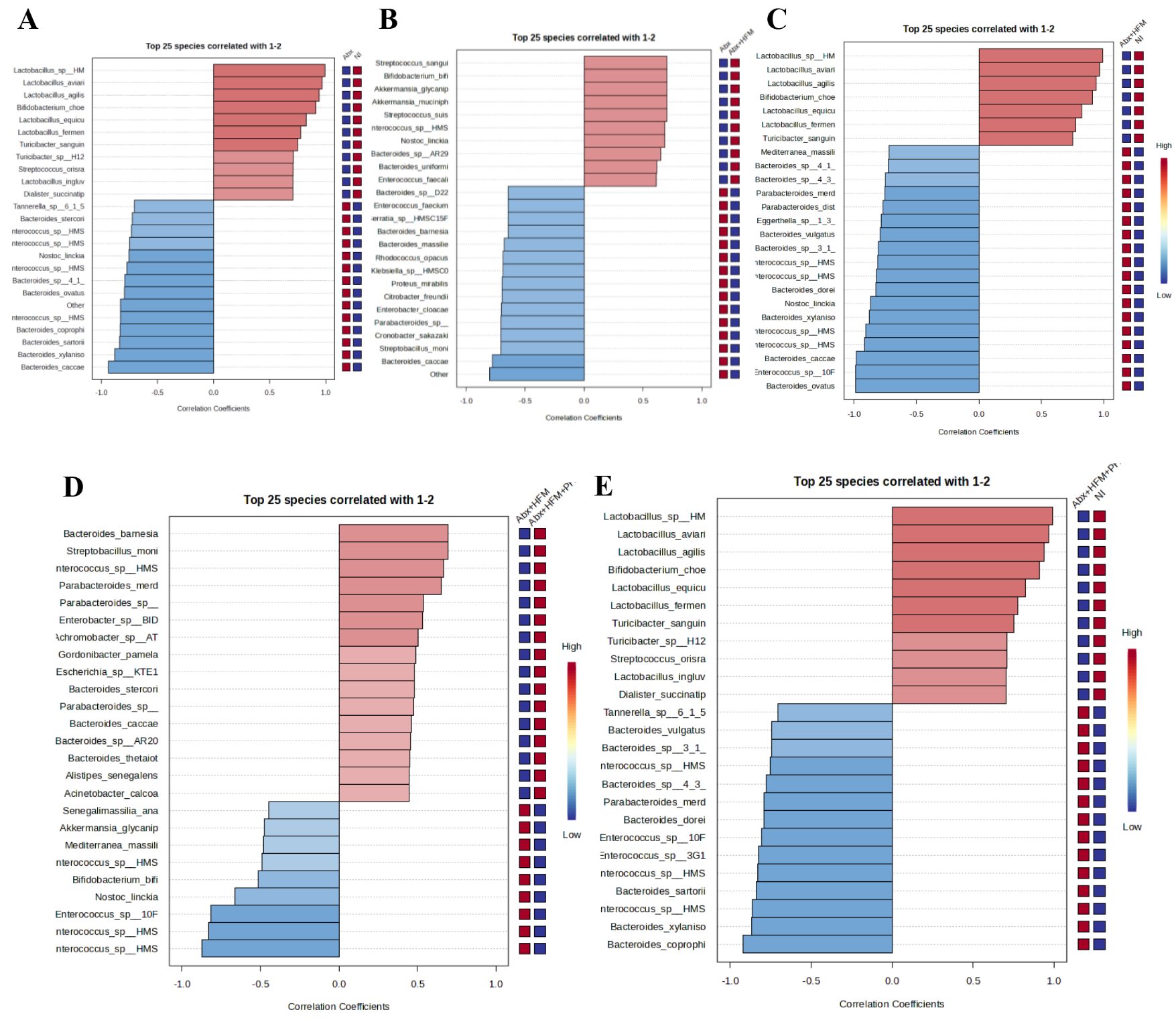
**

**Figure S4**

**
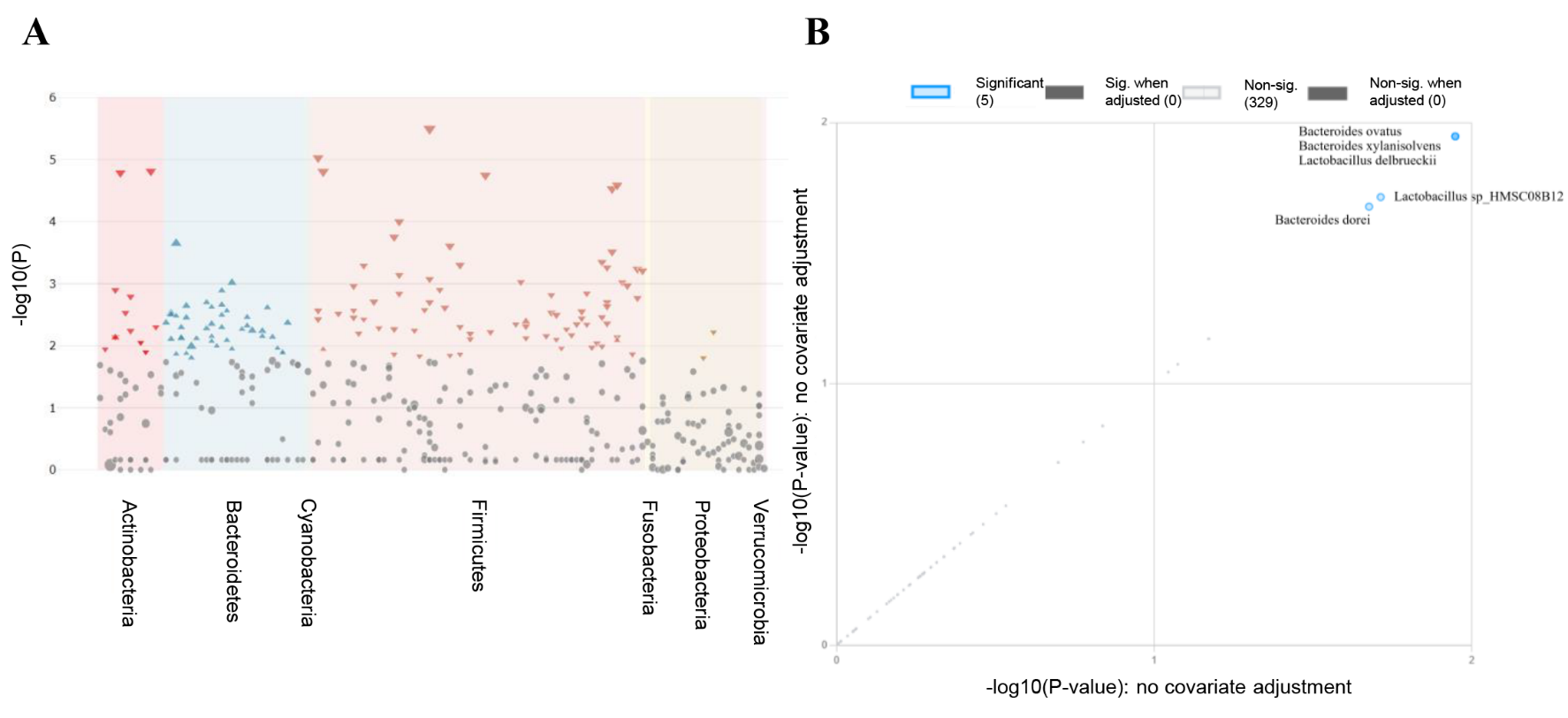
**

**Figure S5**

**
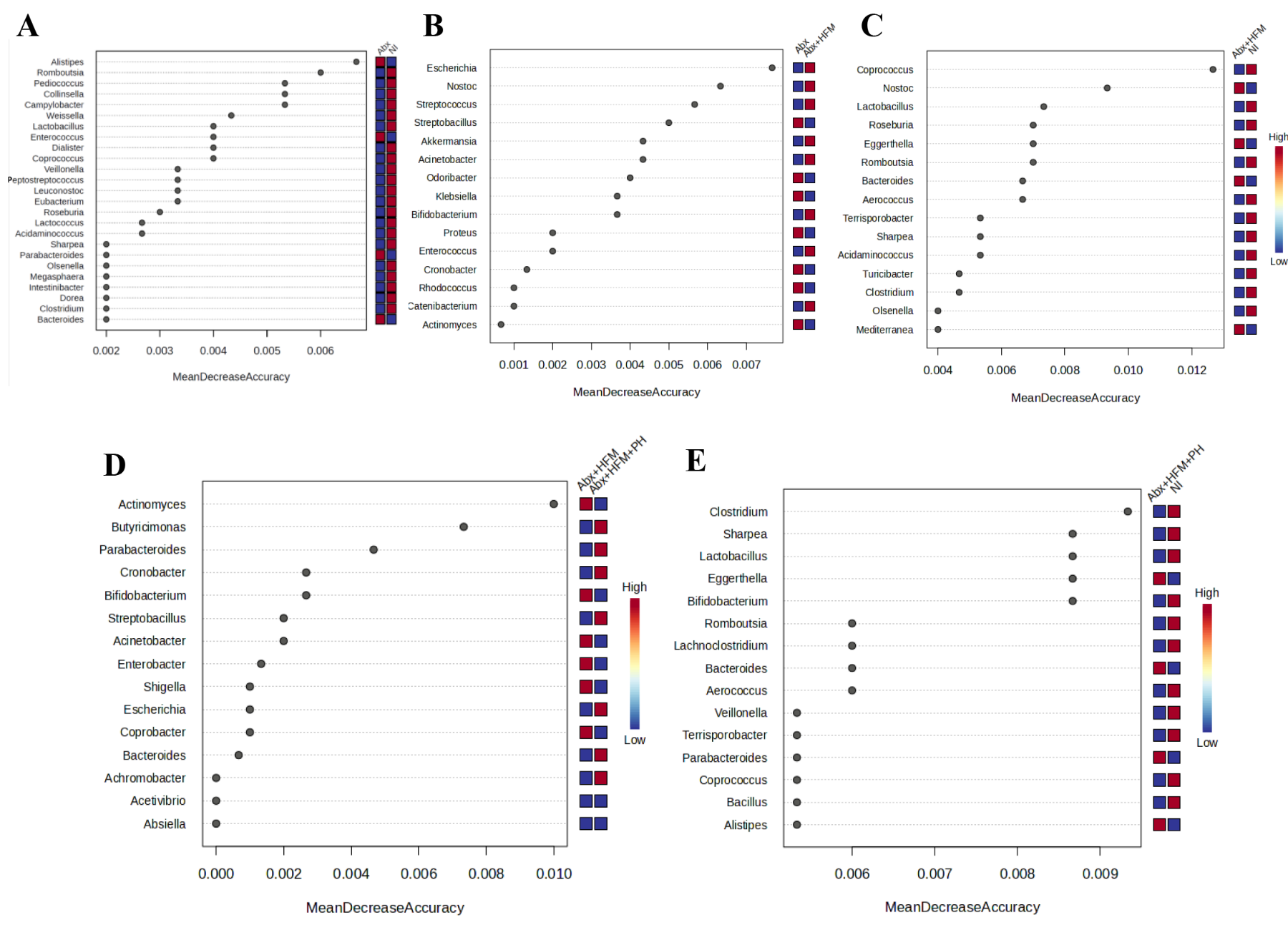
**

**Figure S6**

**
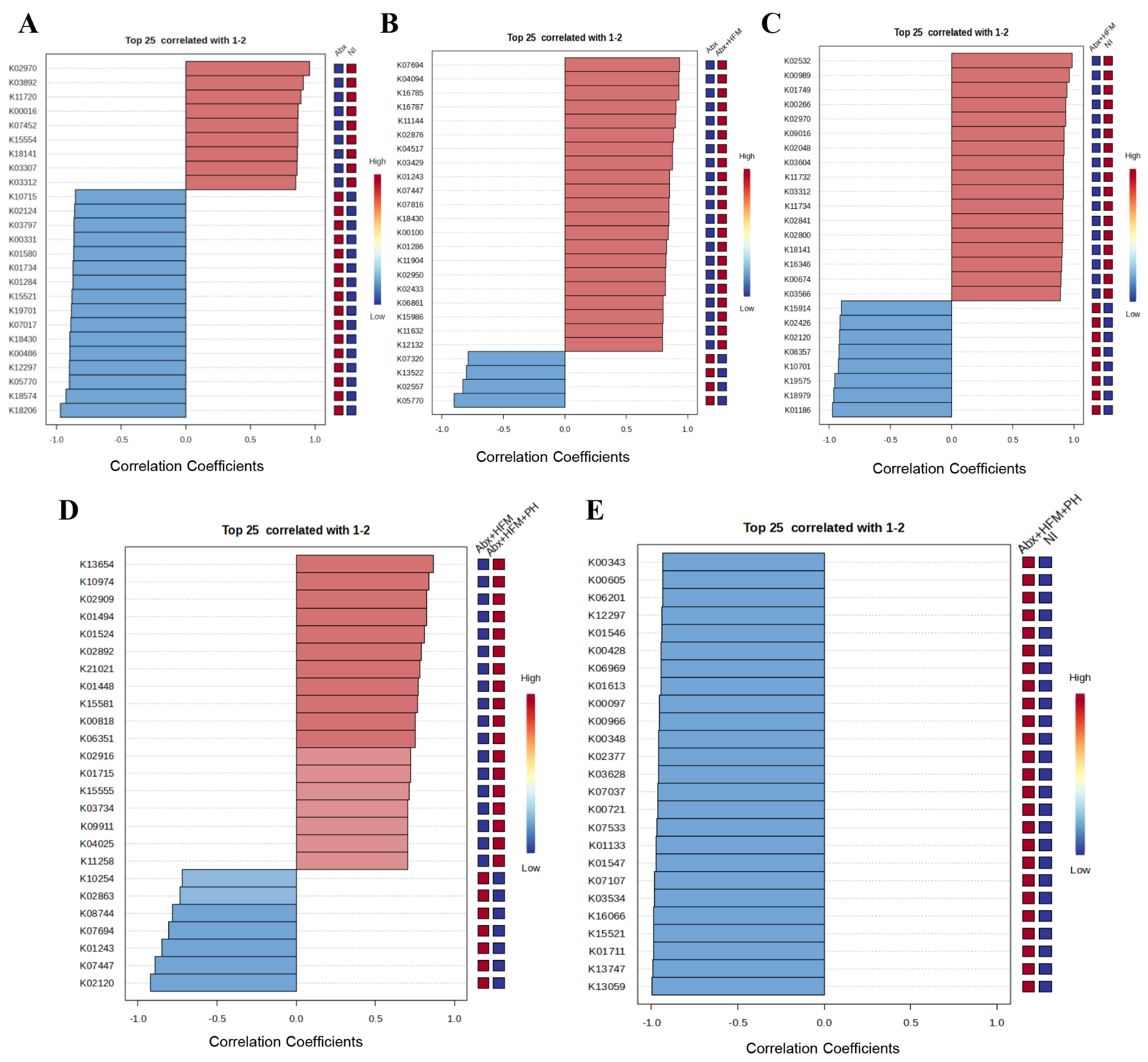
**

**Figure S7**

**
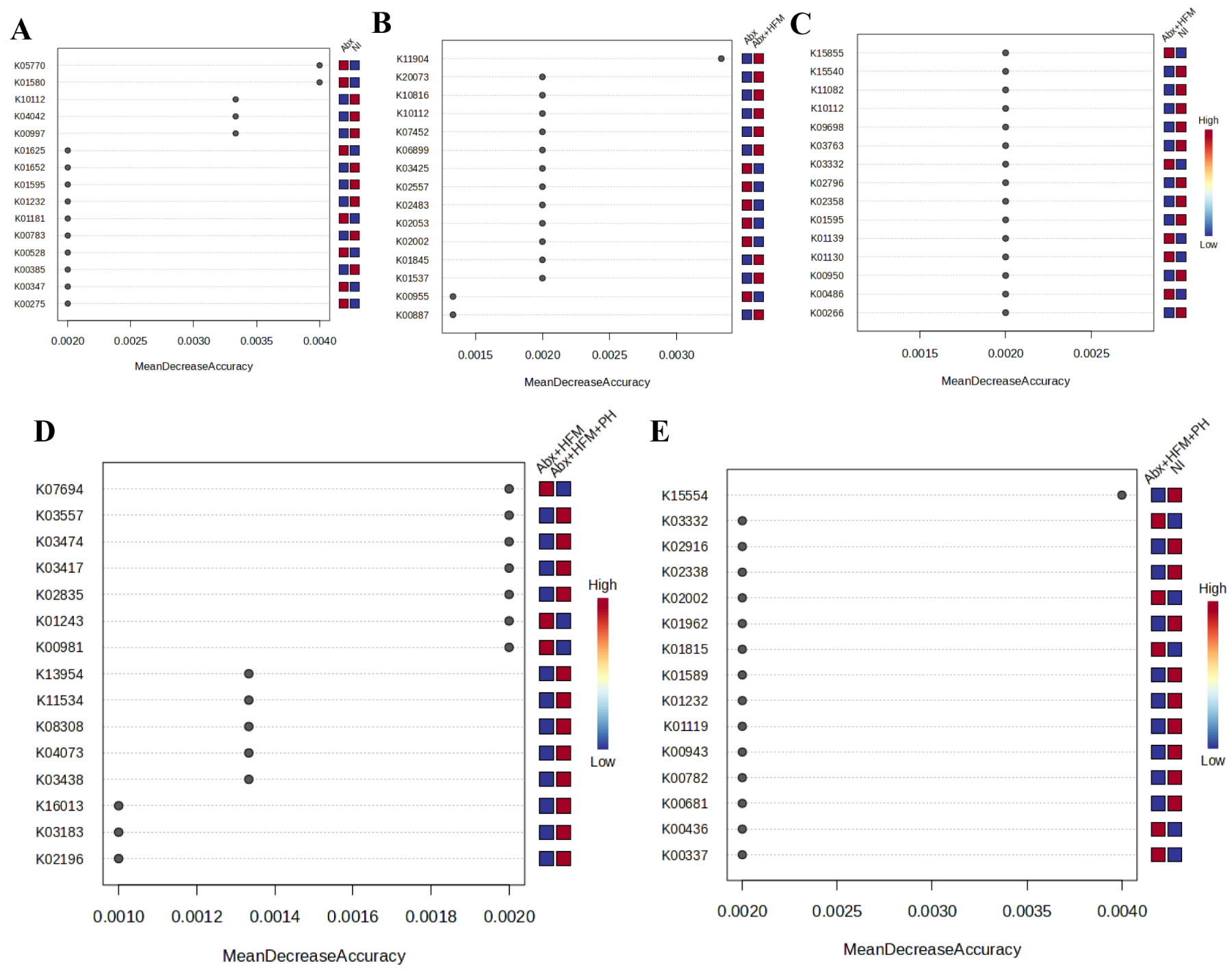
**

**Figure S8**

**
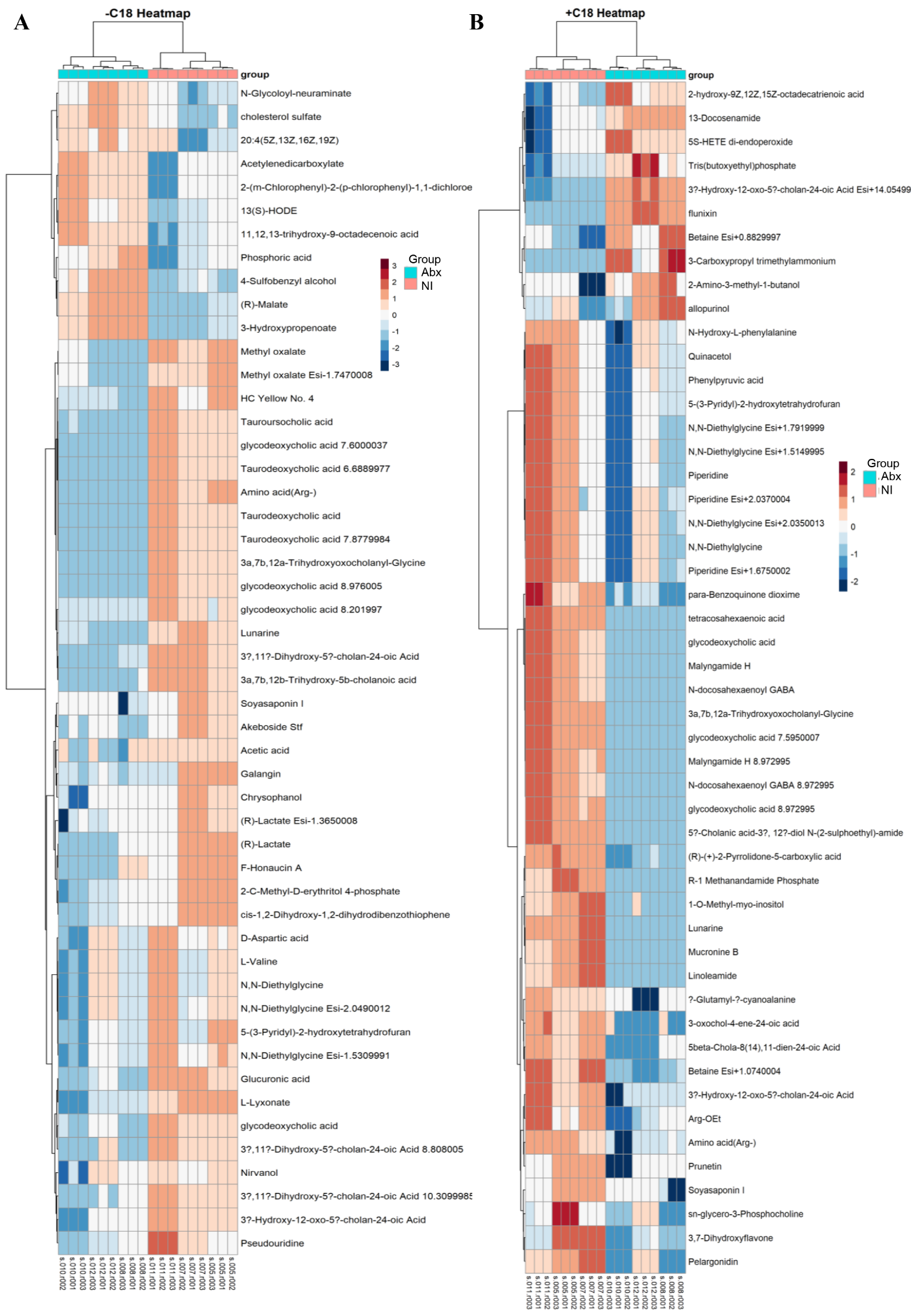
**

**Figure S9**

**
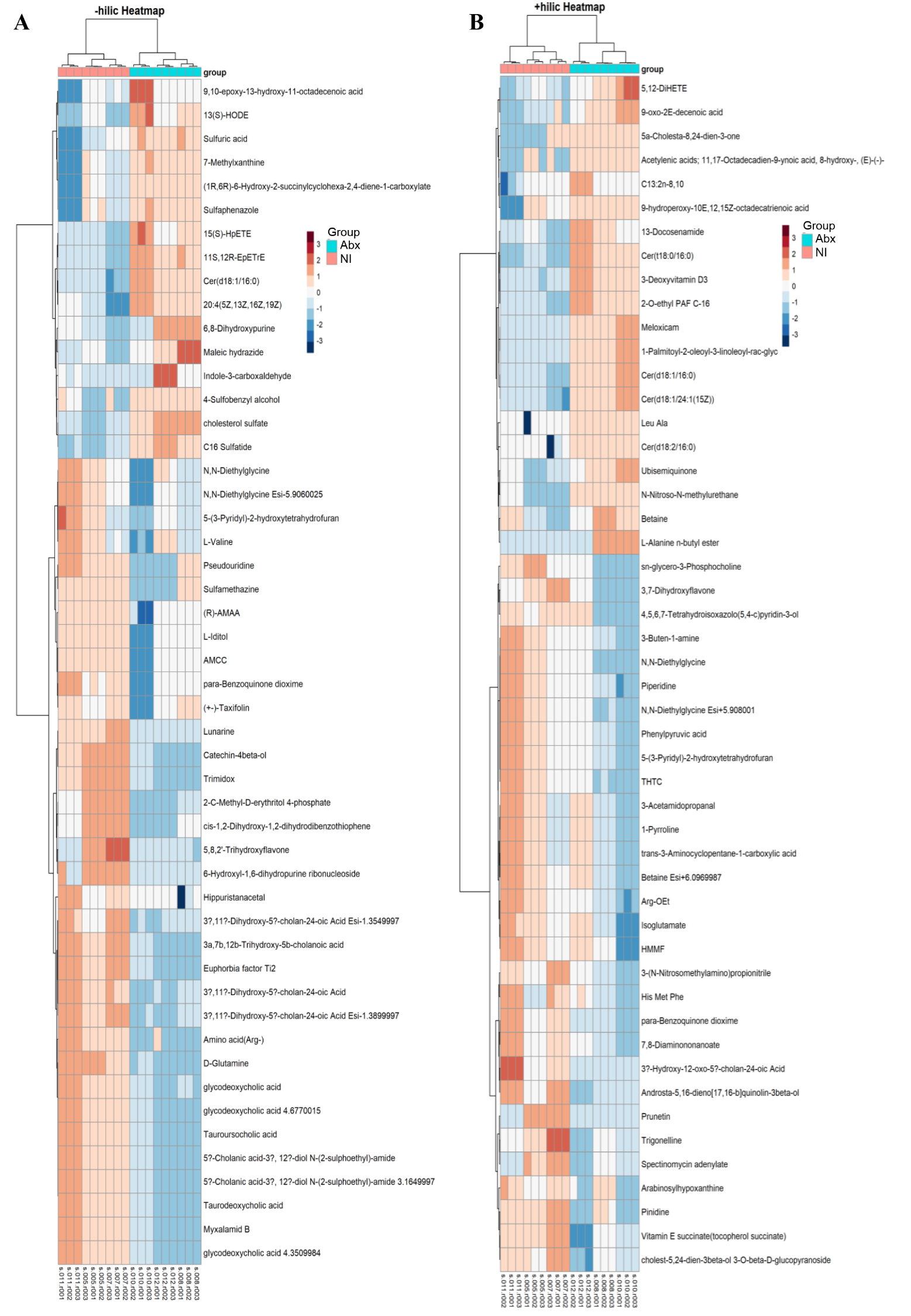
**

**Figure S10**

**
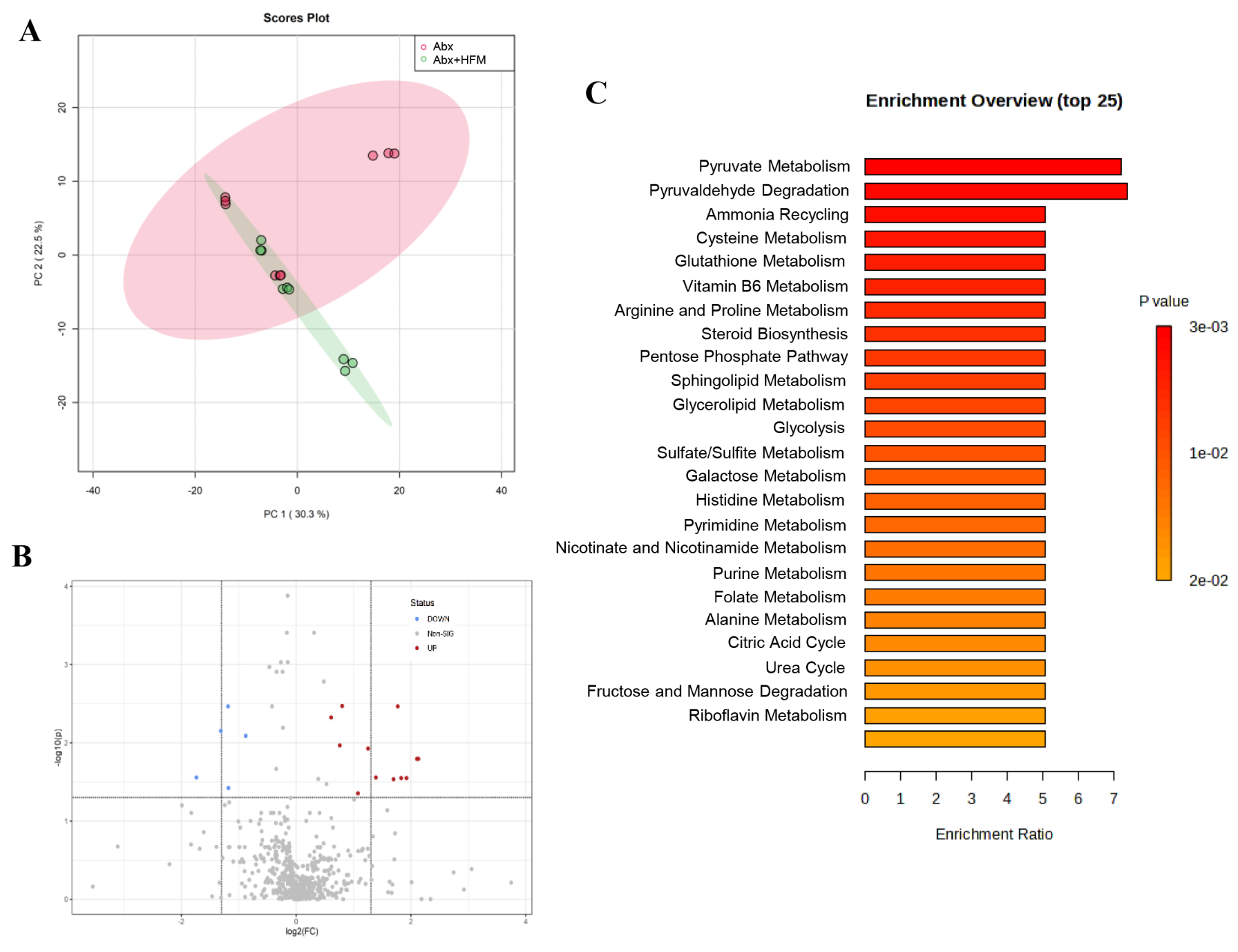
**

**Figure S11**

**
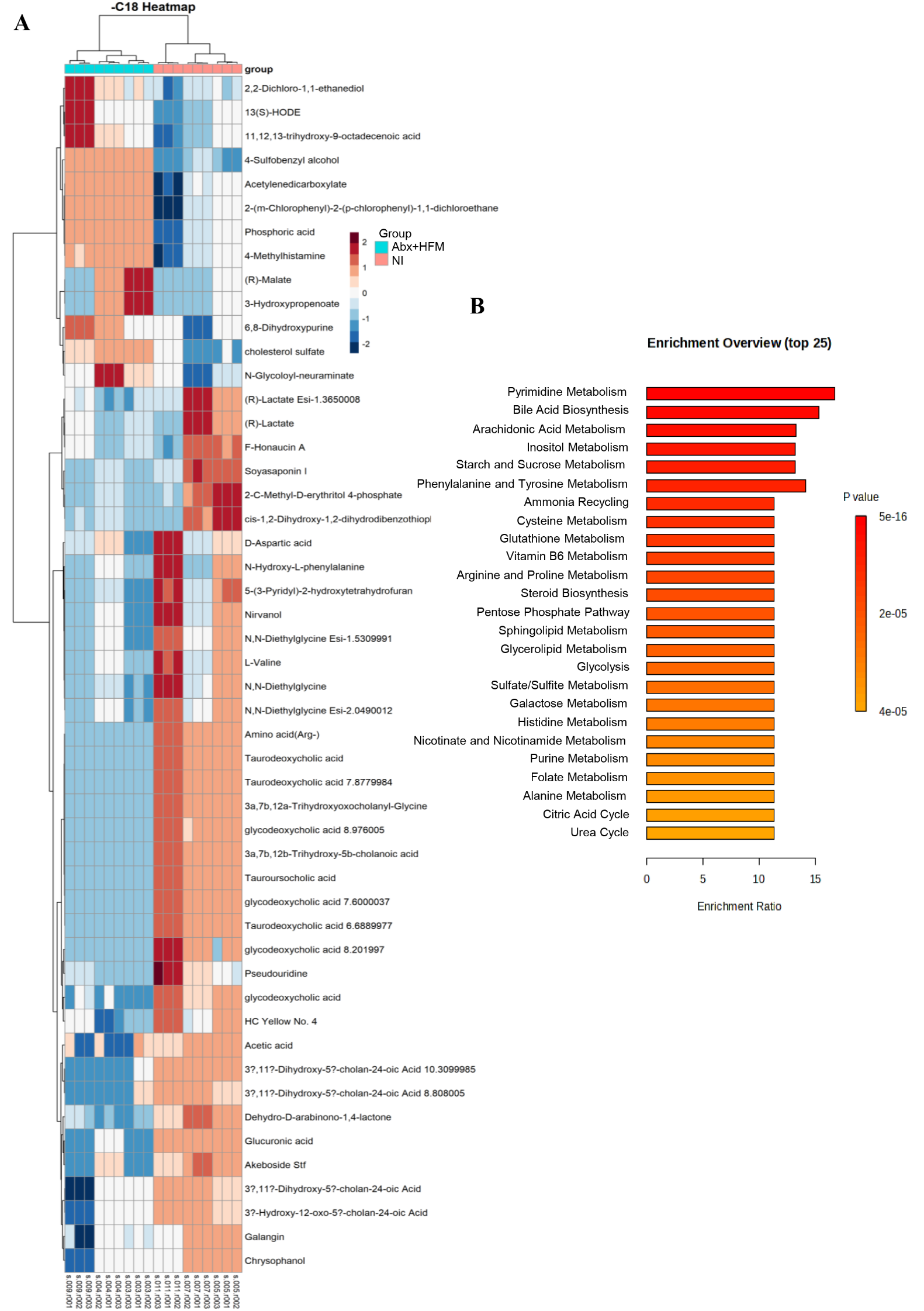
**

**Figure S12**

**
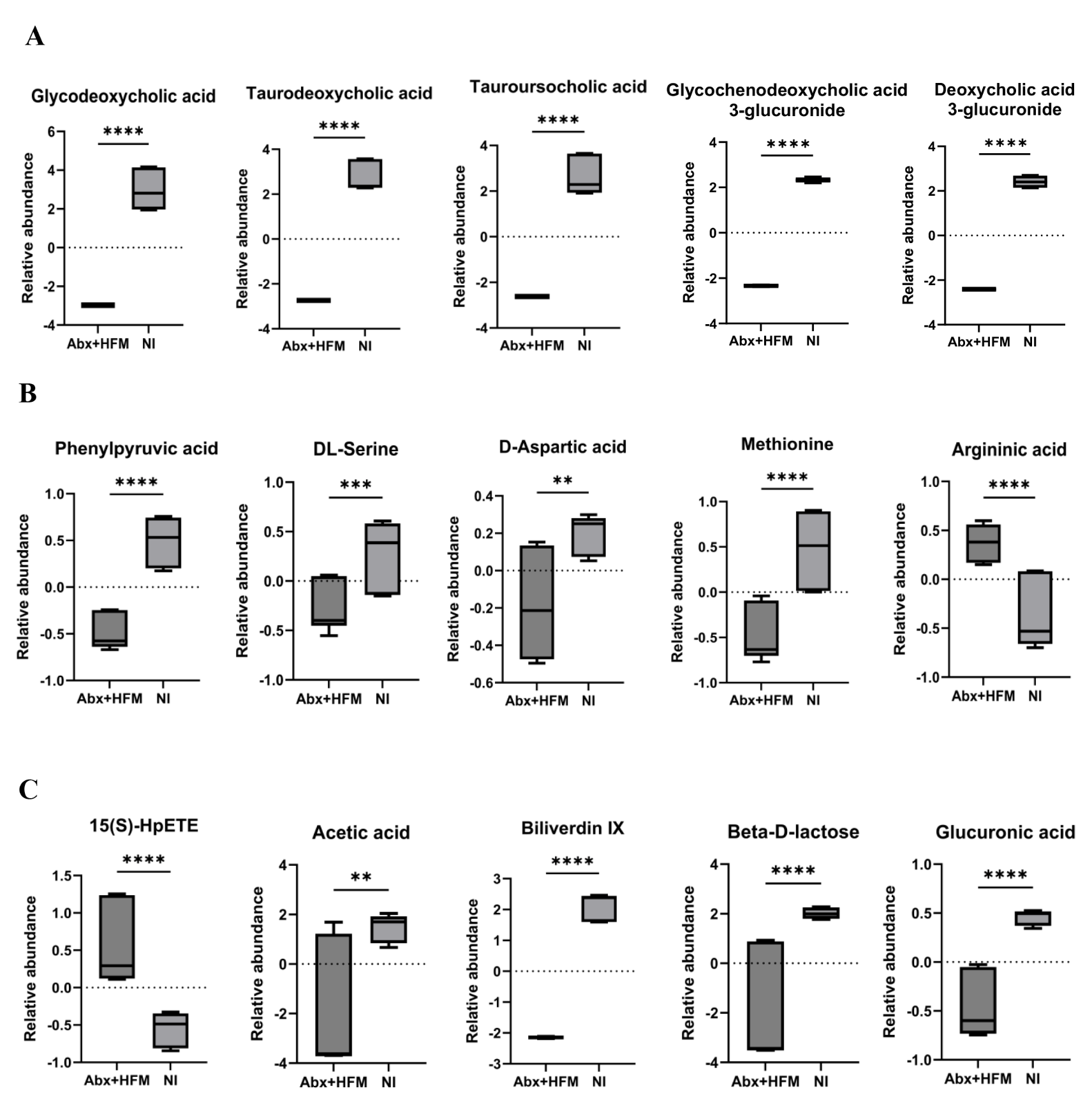
**

**Figure S13**

**
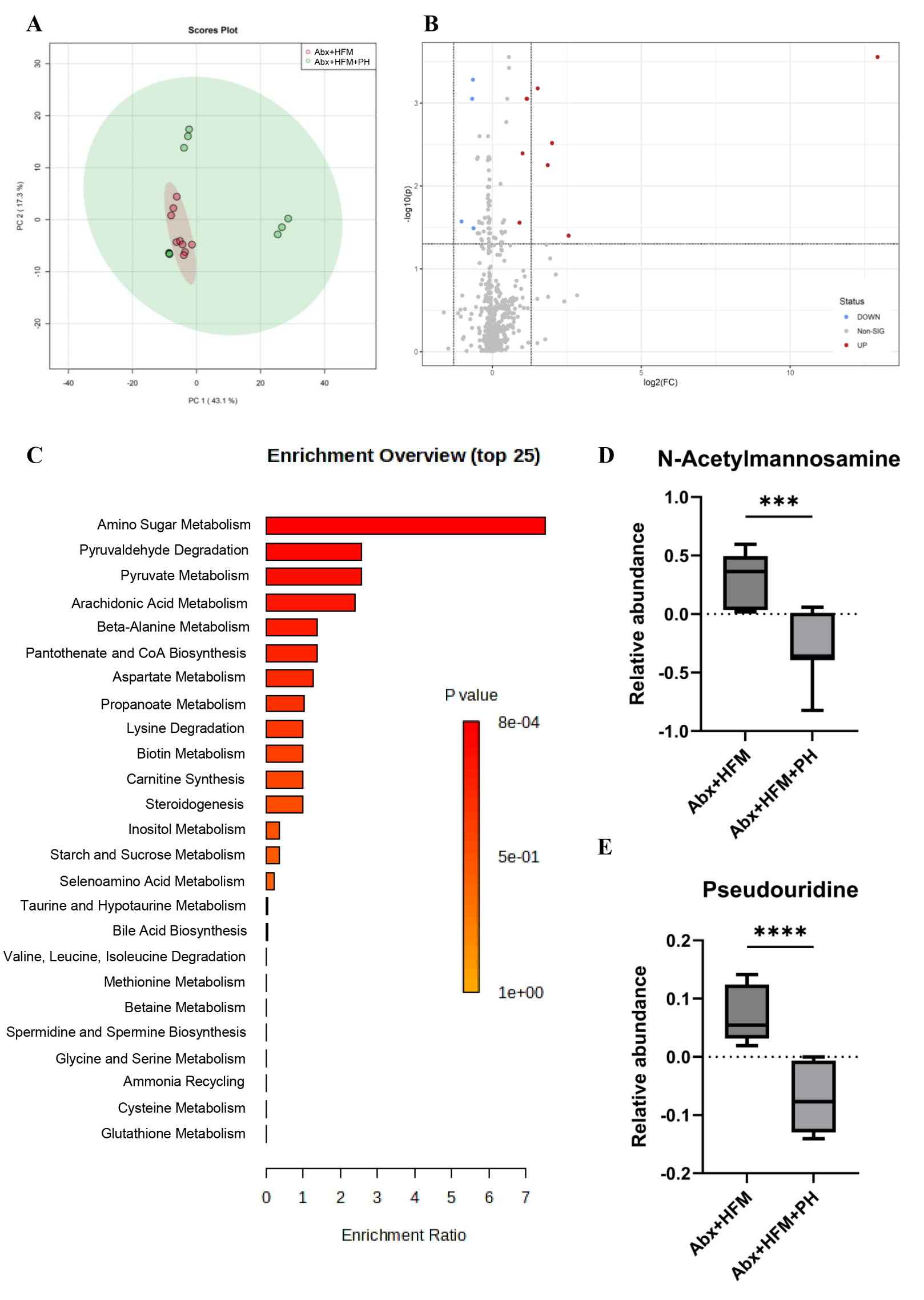
**

**Figure S14**

**
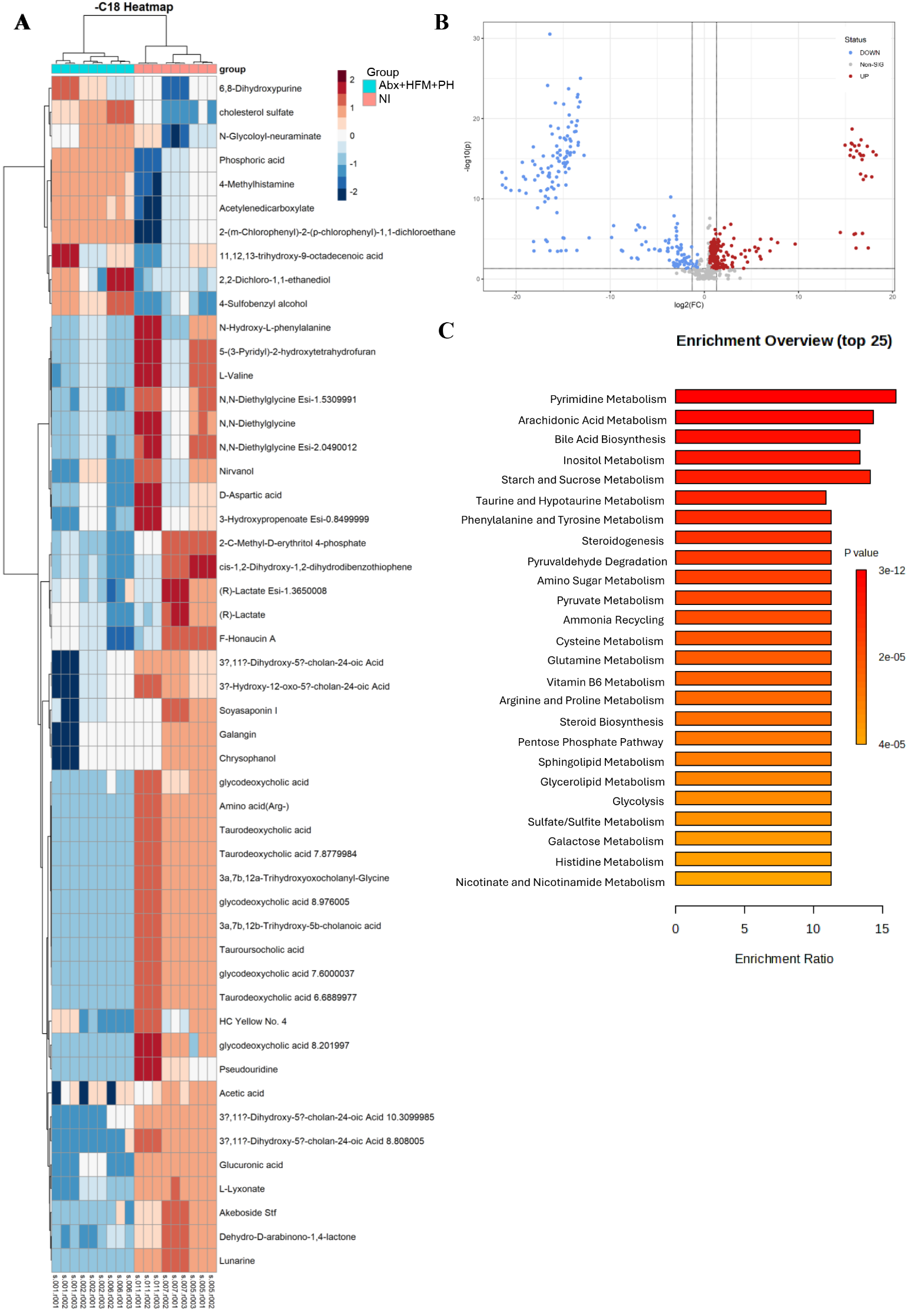
**

**Figure S15**

**
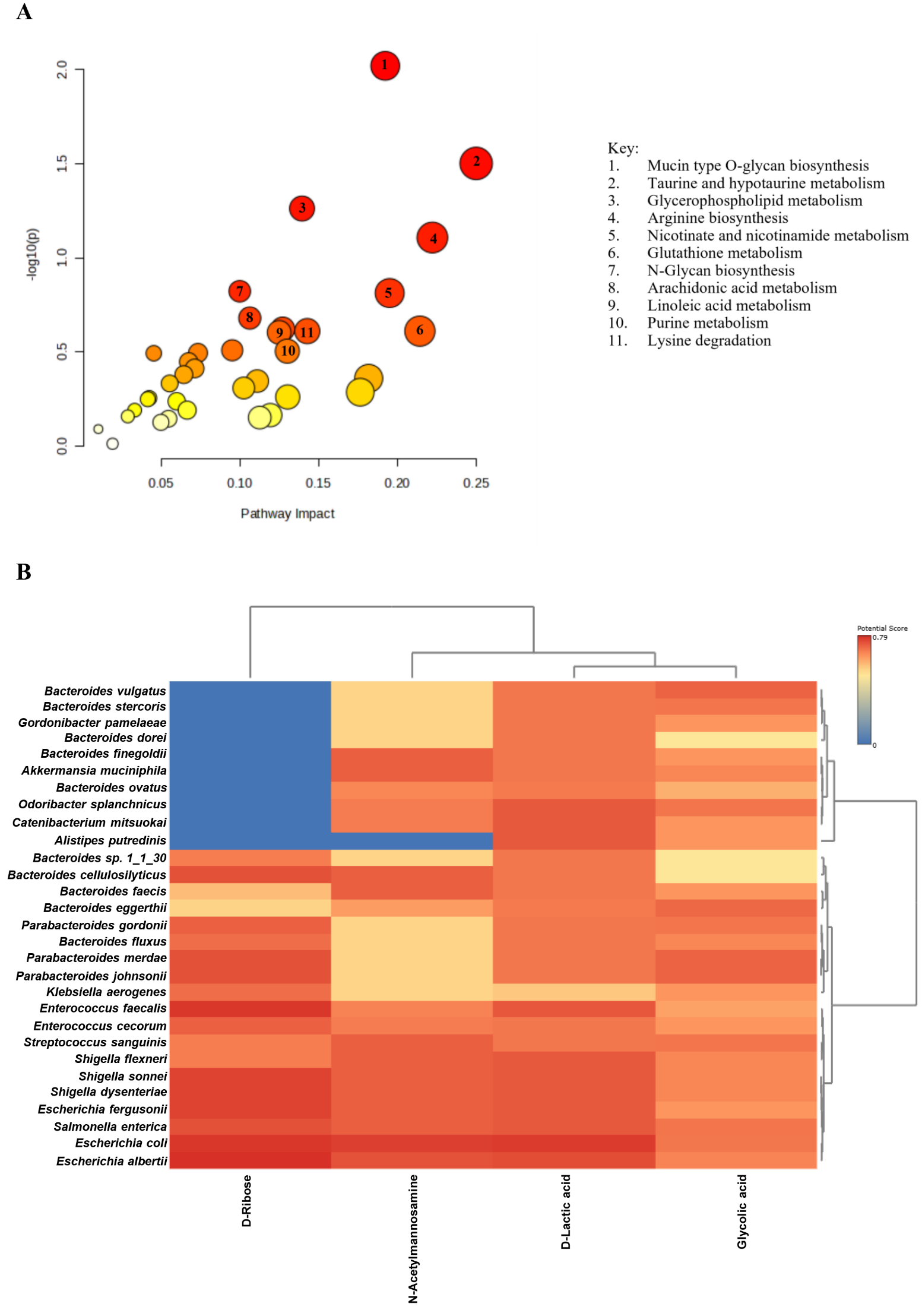
**

**Figure S16**

**
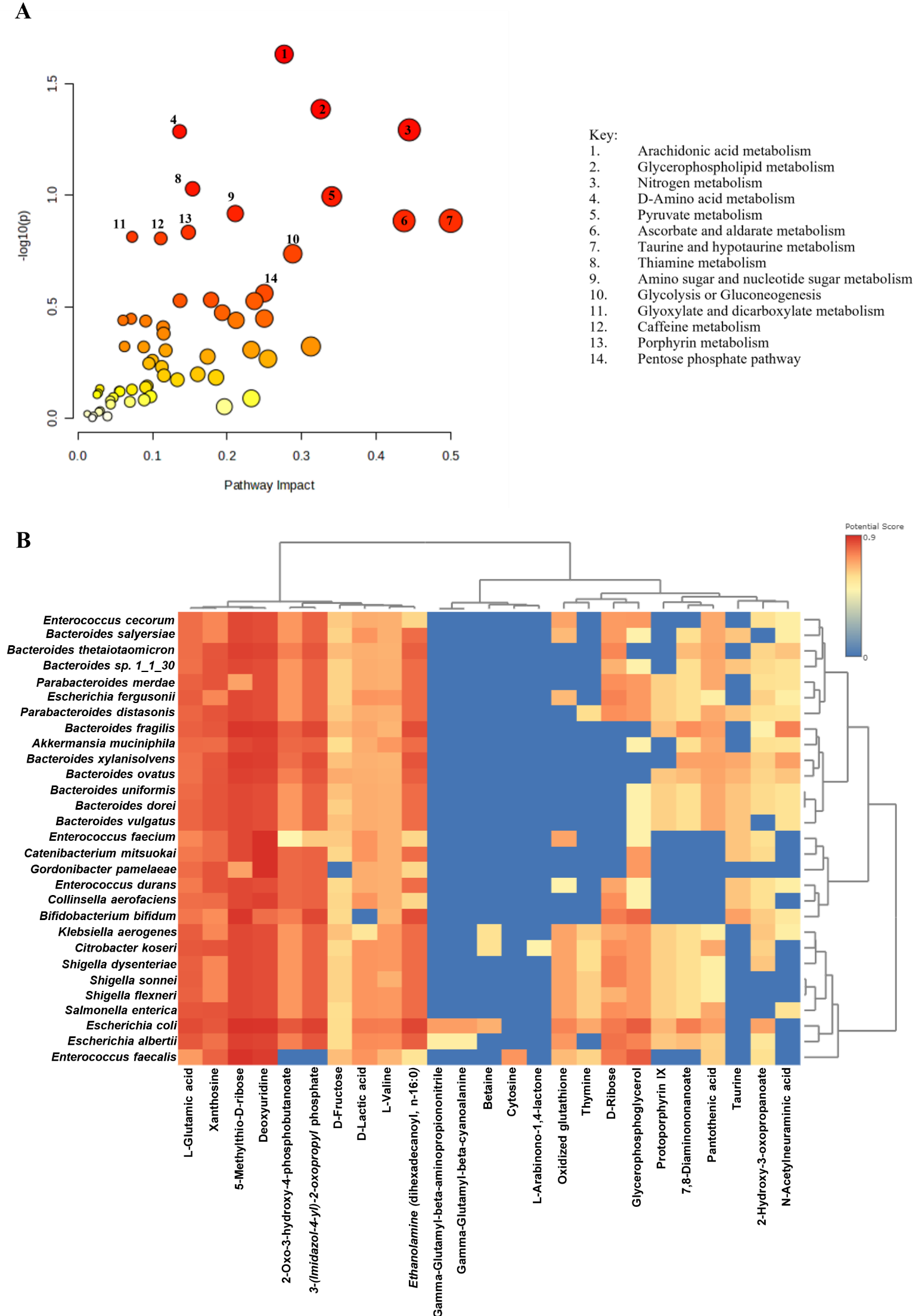
**
